# Mapping Small Effect Mutations in *Saccharomyces cerevisiae*: Impacts of Experimental Design and Mutational Properties

**DOI:** 10.1534/g3.114.011783

**Published:** 2014-04-29

**Authors:** Fabien Duveau, Brian P. H. Metzger, Jonathan D. Gruber, Katya Mack, Natasha Sood, Tiffany E. Brooks, Patricia J. Wittkopp

**Affiliations:** * Department of Ecology and Evolutionary Biology, University of Michigan, Ann Arbor, Michigan 48109-1048; † Department of Molecular, Cellular, & Developmental Biology, University of Michigan, Ann Arbor, Michigan 48109-1048; ‡ Center for Computational Medicine and Bioinformatics, University of Michigan Medical School, Ann Arbor, Michigan 48109-2218

**Keywords:** Bulk Segregant Analysis, Next Generation Sequencing, *TDH3*, Mutagenesis screen, *FASTER MT*

## Abstract

Genetic variants identified by mapping are biased toward large phenotypic effects because of methodologic challenges for detecting genetic variants with small phenotypic effects. Recently, bulk segregant analysis combined with next-generation sequencing (BSA-seq) was shown to be a powerful and cost-effective way to map small effect variants in natural populations. Here, we examine the power of BSA-seq for efficiently mapping small effect mutations isolated from a mutagenesis screen. Specifically, we determined the impact of segregant population size, intensity of phenotypic selection to collect segregants, number of mitotic generations between meiosis and sequencing, and average sequencing depth on power for mapping mutations with a range of effects on the phenotypic mean and standard deviation as well as relative fitness. We then used BSA-seq to map the mutations responsible for three ethyl methanesulfonate−induced mutant phenotypes in *Saccharomyces cerevisiae*. These mutants display small quantitative variation in the mean expression of a fluorescent reporter gene (−3%, +7%, and +10%). Using a genetic background with increased meiosis rate, a reliable mating type marker, and fluorescence-activated cell sorting to efficiently score large segregating populations and isolate cells with extreme phenotypes, we successfully mapped and functionally confirmed a single point mutation responsible for the mutant phenotype in all three cases. Our simulations and experimental data show that the effects of a causative site not only on the mean phenotype, but also on its standard deviation and relative fitness should be considered when mapping genetic variants in microorganisms such as yeast that require population growth steps for BSA-seq.

Characterizing the causal relationships between genotypes and phenotypes is a major goal of modern genetics. Bulk segregant analysis (BSA), in which two phenotypically distinct subpopulations (bulks) of recombinant progeny (segregants) are isolated from a genetic cross and genotyped, is one way to achieve this goal (Michelmore *et al.* 1991). With this method, regions of the genome contributing to the phenotypic difference between the two pools of segregants are identified because causative alleles (and linked loci) occur at different frequencies in the two bulks. BSA is a cost-effective approach to mapping because genotypes are determined only for the two bulk samples rather than each of the individual recombinants. The recent development of high-throughput sequencing, which can be used to determine allele frequencies for nearly all sites in the genome in each phenotypic pool simultaneously, has made BSA particularly effective for mapping polymorphisms in organisms with small genomes such as yeast (Ehrenreich *et al.* 2010; Pomraning *et al.* 2011; Liti and Louis 2012; Wilkening *et al.* 2013). Even small differences in allele frequency between bulks can be detected with this genotyping-by-sequencing approach (Parts *et al.* 2011), allowing detection of small effect variants. Because BSA requires sorting large numbers of individuals based on their phenotype, it is particularly well suited to the analysis of traits that can easily be selected or scored in the laboratory, such as growth in different environments (Wenger *et al.* 2010; Ehrenreich *et al.* 2012; Swinnen *et al.* 2012; Yang *et al.* 2013) or expression of a fluorescent reporter gene (Albert *et al.* 2014).

BSA can be used to identify sites contributing to natural variation (Parts *et al.* 2011; Granek *et al.* 2012; Van Leeuwen *et al.* 2012; Bastide *et al.* 2013) or mutant phenotypes isolated from genetic screens (Wicks *et al.* 2001; Brauer *et al.* 2006; Xia *et al.* 2010). Experimental design and statistical properties of BSA coupled with high-throughput sequencing (BSA-seq) for mapping quantitative trait loci (QTL) have been examined in detail (Magwene *et al.* 2011; Edwards and Gifford 2012); however, methods for mapping mutations using BSA-seq after a mutagenesis screen have received less theoretical attention (but see Birkeland *et al.* 2010). Compared with natural variation, the density of polymorphic sites is usually much lower after a mutagenesis screen, and the mutations are more likely to have effects on fitness. As a result, optimal experimental design and statistical power are expected to be different for BSA-seq when analyzing natural variation and mutant genotypes created by random mutagenesis in the laboratory. For example, sequencing information from linked segregating sites can be combined when mapping natural variation to increase the power of detection (Magwene *et al.* 2011; Edwards and Gifford 2012), but this is usually not possible with the lower genetic diversity present after mutagenesis. In such cases, sequencing coverage sufficient for statistical analysis must be recovered from the causative site itself.

Here, we examine the influence of experimental design and mutational properties on the mapping success of BSA-seq when the density of segregating sites is low, with the goal of providing a general framework for large-scale mapping of small effect mutations after a mutagenesis screen. We describe the effect on mapping sensitivity of four experimental parameters (population size, intensity of phenotypic selection, number of mitotic generations between meiosis and sequencing, and sequencing depth) as well as three mutation properties (effect on mean phenotype, effect on standard deviation of the phenotype, and effect on fitness) that can potentially bias genotype frequencies in the segregant bulks. Previous studies modeling BSA-seq for QTL mapping primarily considered the effects of a genetic variant on the mean phenotype for the trait of interest (Magwene *et al.* 2011; Parts *et al.* 2011).

We used the results from this computational modeling to design a bulk segregant mapping experiment suitable for identifying mutations in yeast causing small changes in expression of a yellow fluorescent protein (YFP) reporter controlled by the *Saccharomyces cerevisiae* *TDH3* promoter. These mutations were previously isolated from a low-dose mutagenesis screen in which each haploid mutant recovered was predicted to have, on average, 47 new mutations with only one affecting fluorescence of the reporter gene (Gruber *et al.* 2012). Our simulations indicated that isolating very large pools of haploid segregants (>10^5^ cells) with extreme fluorescence phenotypes was essential for mapping success given the biological properties of the mutant strains. To achieve this, we developed an experimental system for efficiently collecting phenotypically divergent cells from a population of haploid segregants that uses (i) a genetic background with a greater meiosis rate than the typical laboratory strain (Deutschbauer and Davis 2005), (ii) a robust and tractable mating type marker to efficiently isolate stable haploid bulks (Chin *et al.* 2012), and (iii) fluorescence-activated cell sorting (FACS) for high-throughput phenotyping and selection of individuals with extreme fluorescence levels. Genetic variants responsible for changes in mean YFP expression as small as 3% relative to the wild-type genotype were then successfully mapped despite their significant impact on fitness and confirmed using allele replacement, showing that BSA-seq is a powerful method for identifying small effect mutations after a mutagenesis screen.

## Materials and Methods

### Power analyses

To identify parameters that influenced and maximized power for BSA-seq, we modeled the effects of sequencing depth, phenotypic selection cutoff for choosing bulks, total population size, and generations of growth after meiosis as functions of a causal mutation’s effect on mean expression, standard deviation of expression, and fitness. Because of the low density of mutations expected in mutants isolated from mutagenesis screens, we assumed only one causal mutation influenced the phenotype of interest in each mutant. We also assumed that noncausal mutations were in linkage equilibrium with the causal site. Finally, for simplicity, we assumed that noncausal mutations did not affect fitness. Violating this final assumption should not affect allele frequencies for the causal site as long as it is not linked to these noncausal mutations.

Power analyses were performed in two steps. First, a deterministic model was used to calculate the expected mutant and reference allele frequencies for the causal site in both phenotypically high and phenotypically low bulks prior to sequencing (Supporting Information, Figure S1). Then, using these expected frequencies, sampling was used to account for variation introduced by library preparation, sequencing depth, and allele frequency from sequencing. For each set of parameters, we simulated 1000 sets of reference and mutant allele read counts for both the high and low bulks. These modeling and simulation steps were all performed in R (v 2.14.1; R Development Core Team 2013) and are described fully in File S1 with R code provided in File S2.

### Strains used for mapping

Haploid mutant strains from Gruber *et al.* (2012) with *trans*-regulatory effects on expression of a fluorescent reporter gene in *S. cerevisiae* were used in this study. These mutants were isolated from a low-dose ethyl methanesulfonate (EMS) mutagenesis of a BY4724 (*MAT***a** *lys2**Δ0* *ura3**Δ0*) derivative called YPW1 with a *P*_*TDH3*_*-YFP* reporter gene inserted on chromosome I at position 199,270 (Gruber *et al.* 2012). Based on Canavanine resistance assays, each strain was estimated to contain 47 ± 17 (99% confidence interval) EMS-induced point mutations, with exactly one mutation expected to affect YFP expression in 98.7% of the strains (Gruber *et al.* 2012). A red fluorescent protein (RFP) marker was inserted at the *MAT***a** locus in each of these mutant strains before crossing to the mapping strain described below to avoid diploid contamination when sorting haploid segregant progeny (FASTER MT approach; Chin *et al.* 2012). The genetic basis of altered fluorescence was mapped for the YPW89, YPW94, and YPW102 mutants from Gruber *et al.* (2012), which showed +10%, +7%, and −3% changes in mean fluorescence relative to the nonmutagenized reference strain, respectively (Table 1 and see Figure S2A). These mutants also reduced the standard deviation of fluorescence phenotypes for each strain (Table 1 and see Figure S2B). The mapping strain (*MATα met17Δ0 ura3Δ0 P*_*TDH3*_*-YFP RME1(ins-308A)*) that each of these mutants was crossed to was obtained from a series of crosses involving YPW1 (*MAT***a** *lys2Δ0 ura3Δ0 P*_*TDH3*_*-YFP*), BY4722 (*MATα leu2Δ0 ura3Δ0*), BY4730 (*MAT***a** *leu2Δ0 met17Δ0 ura3Δ0*), and a YAD373 derivative (*MAT***a** *leu2Δ0 ura3Δ0* *RME1**(ins-308A)* *TAO3**(E1493Q)* *MKT1**(D30G)*) from Deutschbauer and Davis (2005). The dominant *RME1**(ins-308A)* allele increased sporulation frequency of heterozygous diploids relative to the starting strain, which facilitated isolating large numbers of spores.

**Table 1:** Fluorescence phenotypes and selection coefficients for the three mutants analyzed

Mutant	Mean Effect			
	%*^a^*	SD*^b^*	SD*^c^*	Selection Coefficient*^d^*
YPW89	+10.45%	+1.38	−5.33%	0.127
YPW94	+7.21%	+0.95	−13.8%	0.130
YPW102	−3.25%	−0.43	−7.49%	0.009

a Mean expression of mutant relative to wild type expressed as a percentage of change in fluorescence phenotype relative to wild type.

b Mean expression of mutant relative to wild type expressed as a number of wild type standard deviation (SD) from wild type mean.

c Standard deviation (SD) of expression phenotype of the mutant strain relative to the reference strain.

d Selection coefficient was measured using competitive growth of each mutant against the control population, as described in the *Materials and Methods*.

### Obtaining bulk segregant populations

For each mutant, the segregating pools were obtained as follows. First, the mutant strain was crossed to the mapping strain on YPD plate and diploid colonies were isolated on SC-Lys-Met medium. A single diploid colony was then inoculated to 2 mL of GNA (5% D-glucose, 3% Difco nutrient broth, 1% yeast extract) and grown to saturation at 30°. Then, 0.2 mL of this culture was diluted into 1.8 mL of GNA and grown 4 hr to log-phase. Next, cells were washed twice in 1 mL of H_2_O, resuspended in 30 μL of H_2_O and spotted on KAc plate to induce sporulation. Sporulation plates were incubated at room temperature without Parafilm sealing to allow oxygenation. Sufficient sporulation (>5%) was usually observed after 4 d, at which point random spores were isolated.

For each strain, the whole yeast spot (about 5 × 10^7^ cells and tetrads) was resuspended in 1 mL of H_2_O in a microcentrifuge tube, washed once in 1 mL of H_2_O and incubated in 200 μL of zymolyase 20T (1 mg/mL) for 50 min on a rotor at room temperature. Once ascus walls were digested, samples were washed in 1 mL of H_2_O and resuspended in 100 μL of H_2_O. To enrich for spores relative to vegetative cells, each tube was vortexed vigorously for 2 min, which resulted in spores visibly sticking to the tube wall; diploid cells remained in suspension. The supernatant was then carefully aspirated off and the spores were washed once more with 1 mL of H_2_O. To release spores from tube walls, 1 mL of Triton-X (0.02%) was added to the empty tubes on ice and samples were briefly sonicated at low power (10 sec at power 3.5 using a Sonic Dismembrator Model 100 from Fisher Scientific). Spore suspensions with nonaggregated cells were observed under a microscope after this step, with less than 5% vegetative contaminants (diploid carryover). Spores suspension needed to be grown to log-phase to express the fluorescence phenotype. To avoid mating during growth, approximately 3 × 10^5^ *MATα* spores were sorted using a BD FACSAria II based on the absence of RFP expression. These cells were then centrifuged, resuspended in 2 mL of YPD (to clear off traces of Triton-X) and incubated at 30° for 14 hr.

Next, log-phase cultures (≈2 × 10^7^ cells/mL) were washed in water and resuspended in 3 mL of SC-Arg (media that has lower autofluorescence than YPD). Cells were acclimated to their medium for 3 hr at 30° before sorting through the FACSAria II instrument. Cytometric gating was set up using FACSDiva software to sort 2 × 10^5^ cells from both tails (<2−5 and >95−98 percentiles) of the YFP distribution in two separate tubes. Only events of intermediate size were sorted based on FSC.A, the area of forward scatter signal (10% tails of FSC.A distribution were discarded) and special care was made to keep the FSC.A median (a proxy for median cell size) the same in the two bulks of cells. Finally, low-fluorescent and high-fluorescent bulks were resuspended in 3 mL of YPD, grown to saturation at 30° and genomic DNA was extracted using a Gentra Puregene Yeast/Bact. Kit from QIAGEN. From this, genomic DNA libraries were prepared using a modified version of a previously described approach (Rohland and Reich 2012) as explained in File S1. Then, 100-bp paired-end sequencing was performed on Illumina HiSeq2000 platform at the University of Michigan Sequencing Core Facility.

### Analysis of Illumina sequencing data

For each sample, FASTQ files containing all paired-end reads data were generated with CASAVA v1.8.2 software. Before alignment, low-quality ends were trimmed from reads using *sickle* v1.2 (https://github.com/najoshi/sickle) with default settings (−q 20 –l 20). Trimmed reads were then aligned to the S288c reference genome (http://www.yeastgenome.org, R64 release from 03-Feb-2011) with *P*_*TDH3*_*-YFP* inserted on chromosome I using Bowtie2 v2.1.0 (bowtie2 −p 2 −x ref.fasta −1 SampleX.R1.fastq −2 SampleX.R2.fastq −I 0 -X 900 −S SampleX.sam –t; Langmead and Salzberg 2012). Next, bamUtil v1.0.9 was used to clip overlaps between mate reads that could bias our estimation of allele frequencies (*bam clipOverlap*–in SampleX.sam–out SampleX.clipped.sam–stats–readName). MPILEUP files containing base calls from overlapping reads at each genomic position were generated with SAMtools v0.1.19 (*samtools view* –q 10 –bS; *samtools sort*; *samtools mpileup* –BD –f; Li *et al.* 2009).

For each mutant, two different MPILEUP files were generated: one was used to call a set of high confidence single-nucleotide polymorphisms (SNPs) using VarScan v2.3.6 (Koboldt *et al.* 2009, 2012; http://varscan.sourceforge.net), and the other was used to estimate allele frequencies for a broader set of genomic positions using Popoolation2 v1.201 (Kofler *et al.* 2011). The first MPILEUP file was obtained from a BAM file containing sequencing data for the mapping strain and another BAM file merging reads for the low and high fluorescence bulks. SNPs were called using VarScan *somatic* and *somaticFilter* commands with the mapping strain considered as “normal” and the merged F_1_ segregant bulks as “tumor” (*somatic*–min-coverage 5–min-var-frequation 0.01; *somaticFilter*–min-coverage 5–min-reads2 3–min-strands2 2–min-var-frequation 0.05). Filtering out sites with strong strand bias was critical to remove false positive variants. In parallel, the second MPILEUP file was generated from two separate BAM files containing reads data for the low and high bulks. Allele frequencies at variable sites were computed using Popoolation2 (*mpileup2sync.jar*–min-qual 20–threads 2; s*np-frequency-diff.pl*–min-count 10–min-coverage 10–max-coverage 500).

Finally, G tests were computed for each variable sites using *likelihood.test()* function from the R package Deducer (Fellows 2012). A large fraction of variable sites identified through Popoolation2 were absent from the set of high confidence SNPs obtained with VarScan and were removed before plotting *P*-values of G-tests. Mutant alleles at these sites were considered as mapping errors as they usually occurred at low frequency, at the end of reads or only in one strand direction.

### Single-site mutagenesis

Targeted mutagenesis was performed using the *delitto perfetto* approach (Storici and Resnick 2006) to introduce each candidate mutation into the genetic background of the mutant ancestor (BY4724 with *P*_*TDH3*_*-YFP* inserted on chromosome I). The *CORE-UK* cassette (*CO*unterselectable *RE*porter *KlURA3* and *kanMX4*) was first inserted at the candidate mutation position in each target gene (*SSN2*, *TUP1*, or *ROX1*) by homologous recombination. Then, coding sequence harboring the candidate mutation was amplified by polymerase chain reaction from the mutant strain (YPW89, YPW94, or YPW102) and introduced in place of the *CORE-UK* cassette. Sanger sequencing of the target gene confirmed allele replacement.

### Expression level of fluorescent reporter for *P_TDH3_* activity

Expression level of the YFP fluorescent reporter protein was quantified in the wild-type, EMS-induced mutants, and single-site mutants using flow cytometry. Eight replicates of each strain and the nonfluorescent BY4724 were arrayed at random positions in a 96-well format on YPG agar rectangular plates (OmniTrays). In addition, 20 replicates of the wild-type fluorescent strain were arrayed at specific positions to control for plate position effects. After growth on YPG, arrayed strains were transferred into 0.5 mL of YPD in a 96 deep-well plate using a V&P Scientific Pin Tool and grown for 20 hr at 30° to saturation. Cells were maintained in suspension by the addition of a 3-mm glass bead in each well and constant shaking at 220 rpm. Immediately before flow cytometry, 20 μL of each culture was diluted into 0.5 mL of SC-Arg in another 96-well plate. Fluorescence was then quantified for an average of 10^4^ events per sample using a HyperCyt Autosampler (IntelliCyt Corp) coupled to a BD Accuri C6 Flow Cytometer (533/30nm optical filter used for YFP acquisition).

Flow cytometry data were analyzed with custom R scripts. First, a set of cytometric events considered as single fluorescing cells was filtered for each sample using Bioconductor *flowCore* and *flowClust* packages. An average of 5 × 10^3^ events per sample were retained after this step. Next, a fluorescence phenotype was calculated for each single event corresponding to log(FL1.A)^2^/log(FSC.A)^3^, which corrected for the correlation between fluorescence level and cell size. FL1.A and FSC.A are the area of the YFP fluorescence signal and forward scatter signal (proxy for cell size), respectively. The phenotype of a given sample corresponds to the median phenotype of all filtered events. Finally, we tested for plate position effects by fitting a linear model to the fluorescence data obtained from the 20 control samples. We included in this model the effects of each plate, row, column and half-plate (plates were scored one half at a time). A stepwise approach based on Akaike information criterion for model selection was conducted using the *step()* function in R and showed that a simple model including only the half-plate effect explained 74.9% of the fluorescence variation across the 20 control samples. Therefore, the effect of each half-plate was extracted from the linear model and subtracted from all samples occurring on the same half-plate. To calculate mean expression relative to wild type, the fluorescence phenotype of the reference strain YPW1 was subtracted from the fluorescence phenotype of each tested strain and this was divided by the difference in fluorescence phenotype between YPW1 and the non-fluorescent control BY4724.

### Fitness assay

Competition experiments were performed to estimate fitness of the parental strain YPW1; the EMS mutants YPW89, YPW94, and YPW102; and the three single-site mutants with mutation in *SSN2*, *TUP1*, and *ROX1*. Fitness was also measured for five mutants randomly chosen from the 179 *trans*-regulatory mutants described in Gruber *et al.* (2012) to estimate the range of selection coefficients for this set of mutants (see Figure S2C). In all cases, fitness was measured relative to a common reference strain expressing GFP (*MAT***a** *lys2Δ0 ura3Δ0 P_*TDH3*_-GFP*) as follows. Each strain was grown for 24 hr to saturation in 5 mL of YPD medium, and then cultures were diluted to 6 × 10^6^ cells/mL based on optical density measurement. A total of 500 μL of each YFP yeast culture was mixed thoroughly with 500 μL of GFP culture and 9 mL of YPD. Next, 500-μL samples of each mix were randomly arrayed in 8 wells of a first 96 deep-well plate and 10-μL samples were diluted to 10^4^ cells/mL into the same eight random wells of a second 96 deep-well plate containing 490 μL of YPD per well. The first plate was used to estimate the proportion of YFP and GFP cells at the beginning of the competition assay (T_0_) using flow cytometry. The second plate was grown for 24 hr at 30° with constant shaking (220 rpm with glass beads to keep cells in suspension). Then, 20 μL of each culture was diluted in 500 μL of YPD in a clean 96-well plate, and the proportion of YFP and GFP cells was estimated at the end of the competition assay (T_1_) using flow cytometry. Fluorescence was recorded for at least 2 × 10^4^ events per sample using a HyperCyt Autosampler (IntelliCyt Corp) coupled to a BD Accuri C6 Flow Cytometer (585/40 nm optical filter used for YFP acquisition and 533/30 nm optical filter used for GFP acquisition). Despite considerable overlap of the YFP and GFP signal detected through the 533/30 nm filter, control experiments showed that cells expressing YFP or GFP could be distinguished using this filter combination. Custom R scripts were used to filter out spurious events from flow data and to compute the proportion of YFP and GFP cells for each sample. A selection coefficient was then calculated for each replicate using the following formula:

$$s=\frac{\left(\text{ln}\left(\frac{YFP_{1}}{GFP_{1}}\right)-\text{ln}\left(\frac{YFP_{0}}{GFP_{0}}\right)\right)}{g},$$

where *YFP*_0_ and *GFP*_0_ are the observed number of cells expressing YFP and GFP at time T_0_ and *YFP*_1_ and *GFP*_1_ are analogous numbers of cells at time T1 for each sample. The experiment was started from an average density of 10^4^ cells/mL and stopped at 6 × 10^7^ cells/mL, yielding an approximate number of generations *g* = 12.55. The selection coefficient obtained by competing mutants expressing YFP against a GFP reference strain can be explained by the mutant genetic background or by YFP expression itself. The fitness effect of the YFP marker was quantified by competing the parental strain YPW1 to the GFP reference strain. From this, the selection coefficient associated with the mutant background was calculated as follows, assuming the effect of EMS-induced mutations and YFP on fitness were additive:

$$s_{mut}=\frac{s+1}{s_{YFP}1}-1,$$

where $s_{mut}$ is the selection coefficient for the mutant background, $s_{YFP}$ is the selection coefficient for the YPW1 reference strain expressing YFP measured in competition to the GFP strain and *s* is the selection coefficient for the mutant strain expressing YFP measured in competition with the same GFP reference strain.

## Results

### Optimizing experimental design for mapping mutations of small effect

**Figure 1:**
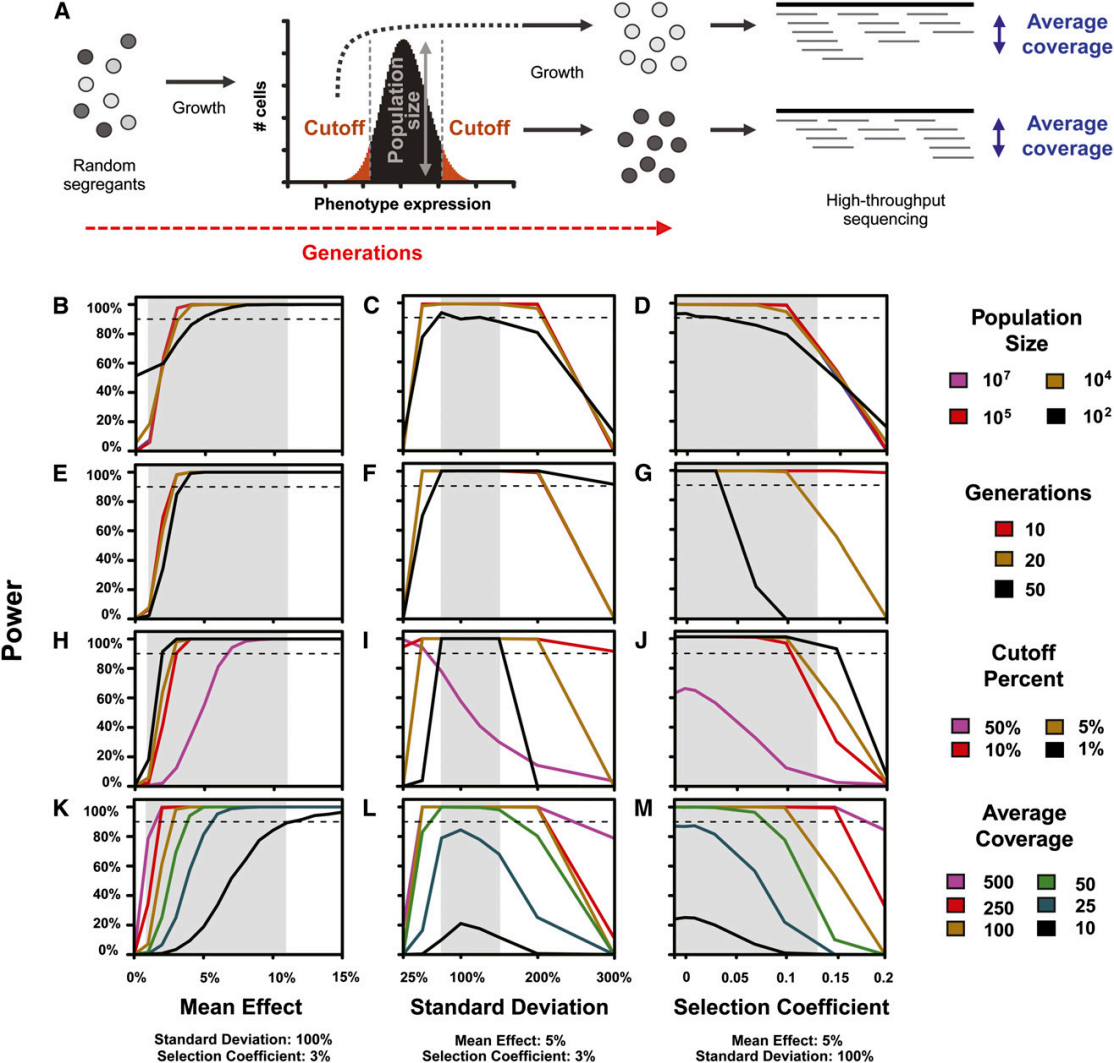
Experimentally controllable parameters affect statistical power for detecting a significant difference in the frequency of a causal mutation between bulks. (A) An overview of the modeled BSA-seq experiment is shown, with the four experimental parameters we allowed to vary (population size, generations of growth, cutoff for bulk selection, and average coverage of sequencing) indicated. Power is shown for various population sizes (B−D), generations of growth (E−G), bulk selection cutoffs (H−J), and average sequencing coverages (K−M), for a range of effects of the causal mutation on mean expression (B, E, H, K), standard deviation of expression (C, F, I, L), and fitness (measured in terms of the selection coefficient) (D, G, J, M). In all plots, the dashed line indicates 90% power. Gray shaded regions represent 90% confidence intervals of the mean effect and standard deviation of the fluorescence phenotypes observed in a recent set of *trans*-regulatory mutants (Gruber *et al.* 2012, see Figure S2, A and B). The 90% confidence interval for selection coefficients was inferred from fitness assays performed on 8 mutants (see *Materials and Methods* and Figure S2C). In all analyses, only the indicated parameters were allowed to vary; all other experimentally controllable parameters were fixed at values ultimately used in our mapping experiment (sequencing depth = 100, population size = 10^7^, cutoff percent = 5%, generations = 20), and mutational parameters were fixed at values representative of the mutants used for mapping (mean effect = 5%, standard deviation = 100%, selection coefficient = 0.03).

To determine how power for detecting different types of mutations varies with mutational properties and experimental parameters, we developed a flexible simulation that models mapping via BSA-seq computationally. This power analysis was parameterized for mapping mutations affecting fluorescence in *S. cerevisiae* that can be efficiently scored in large populations of recombinant cells using FACS but can be adapted to other biological systems with different genome sizes, mutation effects, or attainable population sizes. Variant discovery and allele-frequency estimation were modeled assuming whole genome sequencing of DNA extracted from bulks of recombinants with high- and low-fluorescence phenotypes. We included two phases of cell growth during which competition among genotypes can affect allele frequencies. The first growth phase between meiosis and bulk selection was required to express the fluorescence phenotype and the second between bulk selection and DNA extraction to increase the amount of genomic DNA for sequencing (Figure 1A). Experimentally controllable parameters included in our simulation were population size, intensity of phenotypic selection, sequencing depth, and number of generations between spore isolation and DNA extraction. Innate biological properties of a mutation included were the effects on the mean and standard deviation of the fluorescence phenotype as well as the effect on fitness, which changes the frequency of the mutation during growth. We used the range of biological properties observed for *trans*-regulatory mutants affecting fluorescence of a reporter gene isolated in Gruber *et al.* 2012 (see Figure S2) to identify experimental parameters that provide a high probability of detecting a causal mutation for a minimal cost.

We found that the size of the population from which the bulks were selected did not have a large impact on power as long as the population size was at least an order of magnitude higher than sequencing depth (Figure 1, B−D). When segregant pools were smaller than sequencing depth, a high rate of false-positive results was observed (see Figure S3). For the range of biological parameters observed among mutants isolated in Gruber *et al.* (2012) (shaded areas in Figure 1, B−M), we found that 20 generations or less of mitotic growth provided sufficient power to detect most causal mutations (Figure 1, E−G). Increasing this number to 50 generations allowed competition among genotypes to strongly bias allele frequencies, causing a loss of power to detect mutations with selection coefficients greater than 0.05 (Figure 1G). Our analyses also suggested that selecting cells from the 10% or smaller tails of the fluorescence distribution is sufficient to achieve high detection power under most conditions. Generally, sampling cells from more extreme tails by decreasing the fluorescence cutoff for selection increased power (Figure 1, H−J); however, mutations causing very large increase or decrease in the standard deviation of the fluorescence phenotype were found to require a particular range of cutoff percentages to maximize power (Figure 1I).

Unlike in our simulations, population size, generations of growth, and the fluorescence cutoff for bulk selection are inter-dependent in a real experiment. Starting with a larger population size, for instance, decreases the number of generations of growth required to obtain sufficient cells for analysis, which in turn decreases the impact of the selection coefficient of the mutation on mapping success. However, increasing the population size requires more time and money spent phenotyping individuals before bulk selection. Considering the ease of creating large populations of yeast and the high-throughput phenotyping possible when using FACS to measure fluorescence, we decided to fix the population size at 10^7^ cells and the cutoff for bulk selection at 2% for the rest of this study, resulting in 2 × 10^5^ cells in each bulk. This allowed us to keep the total number of generations to 20, with 10 generations of growth between spore isolation and bulk selection, and 10 generations between bulk selection and DNA extraction.

Using these conditions, we investigated the impact of sequencing depth on mapping power. As expected, increasing the average genome coverage always improved the power to detect causal variants (Figure 1, K−M), but this requires increased cost. Selecting the ideal sequencing coverage therefore depends on the properties of the mutation(s) that the researcher seeks to identify. For mutations similar to those isolated in Gruber *et al.* (2012), we deemed 100× genome coverage the best compromise between power and cost. We note, however, that mutations with a mean effect of 5% or larger can be reliably detected with sequencing coverage as low as 25× (Figure 1K) as long as the mutation does not have a large impact on the phenotypic standard deviation (Figure 1L) or relative fitness (Figure 1M). Mutations with mean phenotypic effects smaller than 2% or with selection coefficients larger than 0.15 would likely require >100× sequencing coverage in each bulk to be mapped.

**Figure 2:**
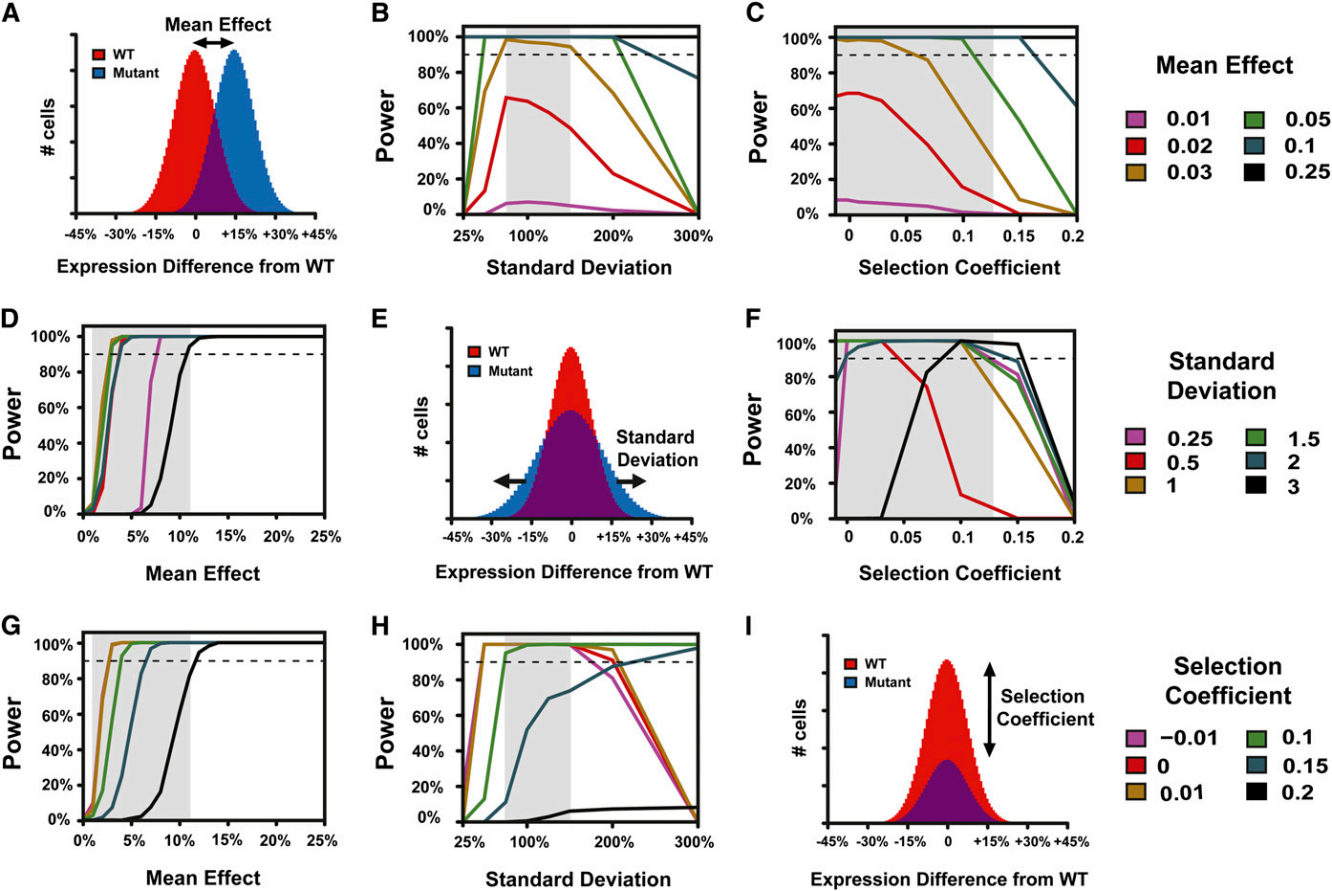
Inherent properties of mutations affect statistical power to detect a difference in the frequency of a causal mutation between bulks. Power is shown for various mutation effects on mean (B, C), standard deviation (D, F), and relative fitness (G, H). Comparisons of hypothetical wild-type (red) and mutant (blue) populations with effects of a mutation on mean expression (A), standard deviation of expression (E), and relative fitness (I) are also shown. In all plots, the dashed line indicates 90% power. Gray shaded regions represent values of the mean effect, standard deviation, or selection coefficient of causal mutations observed in a recent set of expression mutants (see Figure S2). In all analyses, only the indicated parameters were allowed to vary; all others were fixed. These fixed values were: mean effect = 5%, standard deviation = 100%, selection coefficient = 0.03, sequencing depth = 100, population size = 107, cutoff percent = 5%, generations = 20.

With the population size, generations of growth, intensity of phenotypic selection, and sequencing coverage fixed as described previously, we used our simulation to further investigate the relationship between power to detect a causative mutation and that mutation’s effects on mean expression (Figure 2A), standard deviation of expression (Figure 2E), and relative fitness (Figure 2I). We found that mutations that change the mean phenotype at least 3%, cause a phenotypic standard deviation ranging from 75 to 150% of the wild type, and have a selection coefficient less than 0.1 should be detected with a power greater than 90% with this experimental design (Figure 2). This combination of effects on mean and standard deviation includes >90% of the 179 *trans*-acting mutants isolated by Gruber *et al.* 2012 (see Figure S2, A and B). It also includes five of the eight mutants for which we measured relative fitness (see Figure S2C). Fitness measurements for mutants isolated from a mutagenesis screen are expected to overestimate the fitness effects of a causative mutation, however, because mutations that do not affect the phenotype of interest can also affect fitness. Therefore, using the relative fitness of a mutant strain to determine the best experimental design is expected to underestimate the true power for mapping the causative mutation in that strain. Increasing the mean effect of a mutation always improved detection power (Figure 2, B, C, D, and G), but a more complex relationship was observed between the effects of a mutation on the phenotypic standard deviation and selection coefficient. Specifically, when a mutation had a large effect on the standard deviation, more deleterious mutations could have greater detection power than less deleterious mutations under some conditions (Figure 2, F and H). This is because these parameters bias mutation frequency in the two bulks in opposite directions: increasing the selection coefficient lowers the mutation frequency in both bulks, while increasing the standard deviation raises it.

### Identifying single candidate mutations in *trans*-acting mutants

To empirically evaluate the BSA-seq approach using parameters selected based on the simulations described previously, we attempted to map mutations responsible for altered fluorescence in three *trans*-regulatory mutants carrying a YFP reporter protein under the control of the *S. cerevisiae* *TDH3* promoter. Assuming that a single causative mutation explains the phenotypic effects observed in each mutant (Table 1), a mapping power >97% is expected for bulks consisting of 2 × 10^5^ cells sorted from the 2% tails of the fluorescence distribution, with 20 generations of growth and an average sequencing coverage of at least 75× (see Figure S4).

**Figure 3:**
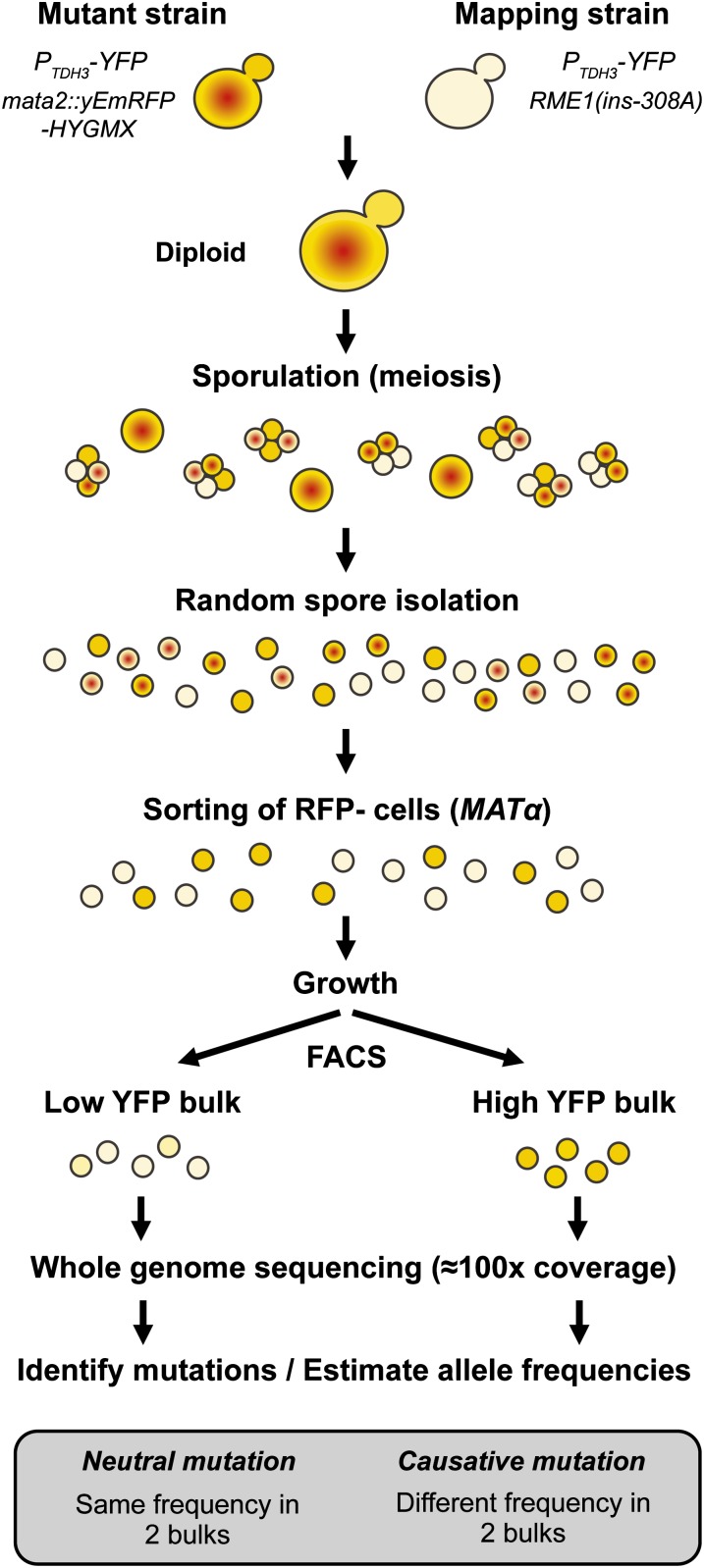
Overview of experimental design for mapping small effect mutations affecting the expression of a fluorescent reporter in EMS-induced mutants. This approach is based on the isolation of a large number of random F_1_ segregant haploid cells, followed by high-throughput phenotypic selection using FACS, and estimation of allele frequencies genome-wide using next generation sequencing. Note the selection of haploid *MATα* cells using expression of the RFP reporter linked to *MATα* locus that is indicated with a red dot. Quantitative differences in the level of YFP expression are indicated by differences in the intensity of yellow background.

To efficiently obtain such large and stringently selected bulks of pure haploid segregants, we followed the protocol shown in Figure 3. Each haploid mutant strain was mated to a common mapping strain and sporulation (meiosis) was induced in the resulting diploids. Including the *RME1(ins-308A)* allele (Deutschbauer and Davis 2005) in the mapping strain increased sporulation frequency from 2 to 20%, making it easier to isolate a large population of F_1_ haploid segregants. To prevent mating between *MAT***a** and *MATα* haploids in the population of segregants, we sorted ∼3 × 10^5^ cells lacking expression of a RFP reporter gene that we had inserted at the *Mata2* locus in each *MAT***a** parent. More than 99.6% of cells lacking expression of this *FASTER MT* marker (Chin *et al.* 2012) were confirmed to be *MATα* haploids. After 10 generations of growth to allow these *MATα* cells to robustly express their YFP fluorescence phenotype, high- and low-fluorescence bulks of ∼2 × 10^5^ cells each were isolated via FACS. Attention was paid during cell sorting to avoid introducing other phenotypic variation between the two bulks. For instance, no more than 1% variation in median cell size (FSC) was allowed between bulks (see Figure S5). After an additional 10 generations of growth, genomic DNA from the low- and high-fluorescing bulks was sequenced to at least 75× coverage (Table 2) using 100-bp paired-end Illumina sequencing.

**Table 2:** Average genome coverage and number of mutations detected from Illumina sequencing of the two segregant bulks for each of the three mutants

	Low Bulk		High Bulk		No.	
Mutant	Mean*^a^*	IQR	Mean*^a^*	IQR	Mutations	G:C ➔ A:T
YPW89	134.1	109−160	111.7	103−126	65	91.0%
YPW94	134.0	107−163	75.3	61−90	73	84.4%
YPW102	91.9	74−109	132.2	121−147	33	84.9%

IQR, interquartile range of genome coverage.

a Mean coverage obtained after read alignment using *genomecov* tool from BEDTools.

**Figure 4:**
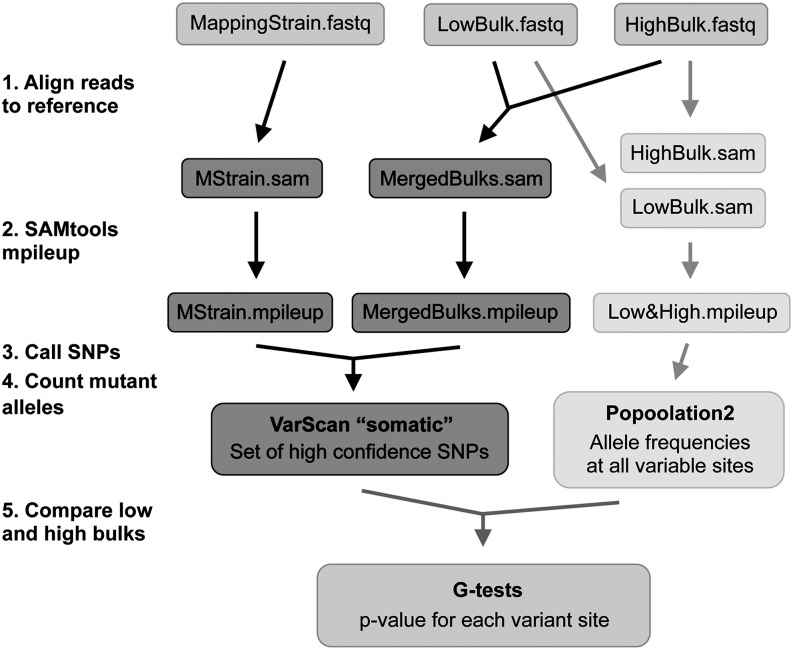
Analysis of Illumina sequencing data. A set of high confidence variants was called using the *somatic* command in VarScan (dark gray), with reads from the mapping strain treated as “normal” data and reads from merged bulks treated as “tumor” data. Allele frequencies were then estimated for these sites in the low fluorescence and high fluorescence bulks with Popoolation2 (light gray). Differences between these two bulks were assessed using G-tests.

Sequencing data were analyzed using a pipeline with two main parallel steps (Figure 4). First, a set of high confidence variants was called for each mutant with VarScan (Koboldt *et al.* 2012, 2009) based on genome sequencing data from the mapping strain and genomes of the segregant bulks, with reads from both bulks merged. The number of mutations identified in each mutant ranged from 33 to 77 (Table 2), which closely matches the number of mutations (30−64) predicted using Canavanine-resistance mutation rates in Gruber *et al.* (2012). Most of these mutations (85–94%) were G:C to A:T transitions, as expected for EMS-induced mutations (Table 2). Allele frequencies were then estimated at every variable site for each bulk using Popoolation2 (Kofler *et al.* 2011). These allele frequencies were strongly correlated (r = 0.983) with independent estimates determined by pyrosequencing (see Figure S6 and File S3). The number of reads containing reference and mutant alleles at high confidence polymorphic sites was compared between low and high fluorescence bulks using a two-sided G-test.

**Figure 5:**
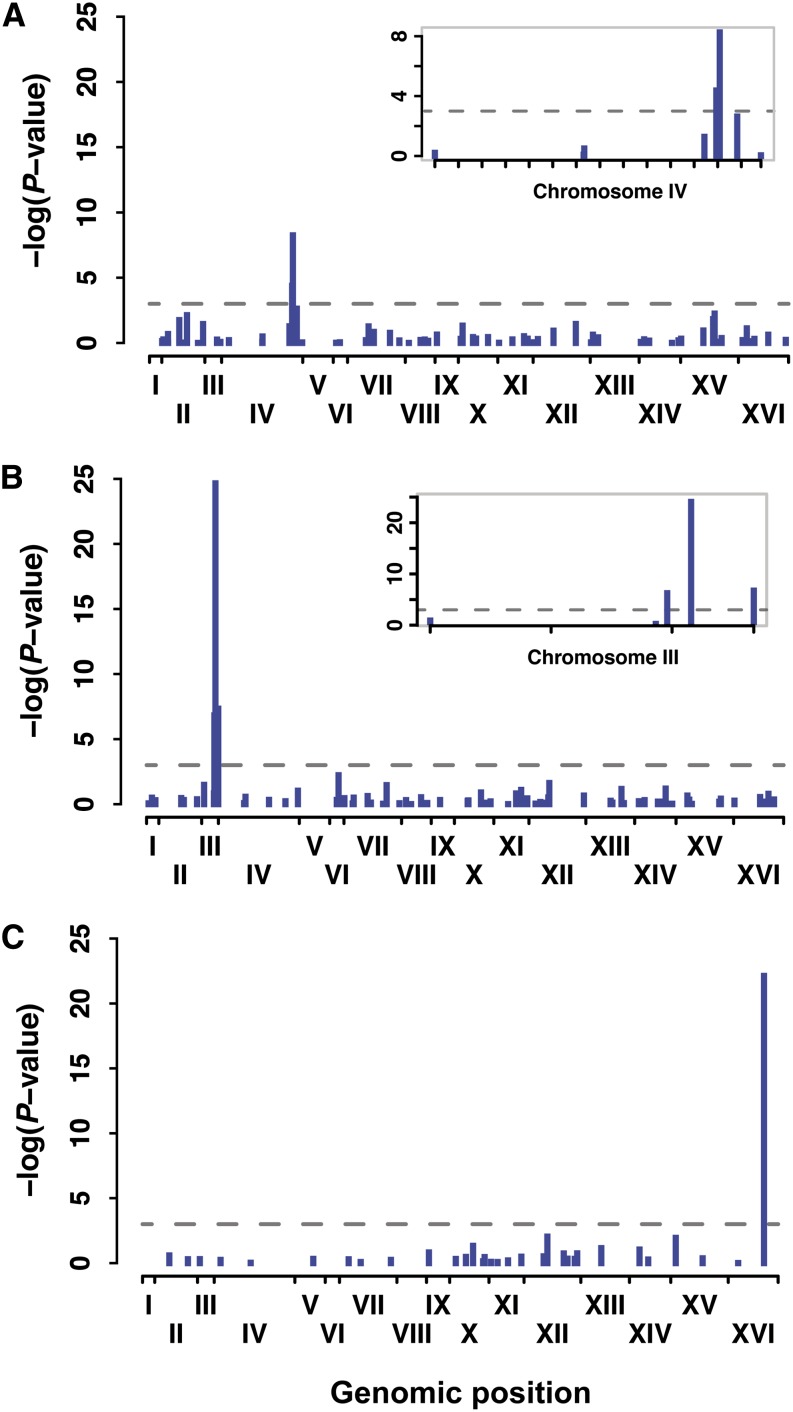
BSA-seq clearly identified a single causative site in three *trans*-regulatory mutants affecting fluorescence of a reporter gene. Significance of the difference in allele frequency between low fluorescence and high fluorescence bulks is shown as the negative of logarithm of *P*-value from G-test for mutants YPW89 (A), YPW94 (B), and YPW102 (C). Each bar shows significance for an individual EMS-induced mutation with its genomic position represented on *x*-axis. Roman numerals indicate each of the 16 *S. cerevisiae* chromosomes. Insets in (A) and (B) are magnifications of chromosomes harboring causative sites and show linked mutations with significant effects. Horizontal dotted lines represent a significance threshold of α = 0.001.

For each mutant, we observed a single, highly significant (*P* < 0.001) association with YFP fluorescence level (Figure 5); physically linked sites also showed significant associations with comparatively higher *P*-values (Figure 5). To determine the likelihood that similar associations would have been detected for other mutants, we examined the distribution of aligned sequence reads in more detail (see File S4). We found that ∼3% of the genome had little-to-no sequencing coverage in each mutant (see Figure S9) and that this was due to difficulty obtaining and/or aligning sequence reads from these regions rather than stochastic fluctuations in coverage due to sampling (see Figure S10, A and B). We also found that reducing sequencing depth would have caused us to miss the significantly associated site in YPW89—the mutant with the largest effect on mean fluorescence of the *P_TDH3_-YFP* reporter gene—because of its strong deleterious effect on fitness (see Figure S10, C and D).

The sites with the strongest statistical associations in YPW89 (Figure 5A), YPW94 (Figure 5B), and YPW102 (Figure 5C) were nonsynonymous substitutions affecting the *SSN2*, *TUP1*, and *ROX1* genes located on chromosomes IV, III, and XVI, respectively (Table 3). These three mutations are all coding substitutions, which is not surprising given that open reading frames constitute 73% of *S. cerevisiae* genome (*Saccharomyces* Genome Database). *TUP1**(G696D)* and *ROX1**(R12K)* are both missense mutations that change one amino acid, whereas *SSN2*(Q971*) introduces an early stop codon truncating 450 amino acids of the protein. All three mutations affect amino acids that are highly conserved across *Saccharomyces* species, with *TUP1**(G696D)* and *ROX1**(R12K)* substitutions predicted to be deleterious using SIFT (SIFT score = 0 for both; Kumar *et al.* 2009). The fitness estimates of each substitution are similar to the fitness consequences reported for deletion alleles of these genes (W = 0.859, 0.954, and 0.983 for single-site substitutions in *SSN2*, *TUP1*, and *ROX1*; W = 0.896, 0.921, and 0.971 for deletions of *SSN2*, *TUP1*, and *ROX1* [Breslow *et al.* 2008; Deutschbauer *et al.* 2005]), suggesting that the mutations observed severely impair the function of the corresponding proteins. Interestingly, *TUP1* appears to be a direct regulator of *TDH3* expression: *TUP1* protein is a general transcriptional repressor that was shown to directly bind *TDH3* promoter in ChIP-chip experiments (Hanlon *et al.* 2011). *ROX1* is a repressor of hypoxic genes that might indirectly affect *TDH3* expression through the regulation of Pdr1 transcription factor (Harbison *et al.* 2004; Larochelle *et al.* 2006). The Tup1-Ssn6 complex is also a well-established regulator of *ROX1* expression (Mennella *et al.* 2003), suggesting that Tup1 acts at multiple levels of the regulatory network controlling *TDH3* expression. Finally, *SSN2* encodes a facultative subunit of the RNA polymerase II holoenzyme (Song *et al.* 1996), which could potentially act on several components of the *TDH3* regulatory network.

**Table 3:** Properties of the three causative sites identified by BSA-seq and confirmed by single site mutagenesis

Mutant	Mutation Position		Mutation Type			Phenotypic Effect			Sequencing Depth*^d^*		Mutation Frequency	
	Chr.	Position	Gene	DNA	Protein	Mean*^a^*	Std Dev*^b^*	Sel Coef*^c^*	Low Bulk	High Bulk	Low Bulk	High Bulk
YPW89	IV	1347028	*SSN2*	C→T	Q971Stop	+10.16%	−6.66%	0.140	121	83	0	0.22
YPW94	III	260366	*TUP1*	G→A	G696D	+6.93%	−14.39%	0.045	152	73	0	0.55
YPW102	XVI	679727	*ROX1*	G→A	R12K	−4.05%	−8.89%	0.015	102	77	0.96	0.30

BSA-seq, bulk segregant analysis coupled with high-throughput sequencing; Chr., chromosome.

a Mean expression of single site mutant relative to wild type expressed as a percentage of change in fluorescence phenotype relative to wild type.

b Standard deviation of expression phenotype of the single site mutant strain relative to the reference strain.

c Selection coefficient was measured by using competitive growth of each single site mutant against the control population, as described in the *Materials and Methods*.

d Number of sequencing reads overlapping the variable site in each bulk.

### Validating bulk segregant mapping results

In parallel to the method described previously, we used a more traditional mapping approach involving tetrad dissection (Birkeland *et al.* 2010) to analyze the YPW89, YPW94, and YPW102 mutants. Each mutant was crossed to a common mapping strain (see File S1); the resulting diploids were sporulated, and a dozen tetrads were dissected. For each tetrad, the fluorescence phenotype of each spore was determined by measuring mean fluorescence of their haploid mitotic progeny using flow cytometry, and the two colonies most likely to carry the causative mutation were identified manually (see Figure S7). Segregant progeny deemed to show the mutant phenotype were pooled together, genomic DNA from this pool was extracted and subjected to Illumina sequencing, and allele frequencies were estimated for each variable site. G-tests were used to compare the observed mutation frequency at variable sites to a null model with a frequency of 0.5.

For YPW89, YPW94, and YPW102, the best candidate mutations identified with the BSA-seq approach were also highly significant with the tetrad dissection method (see Figure S8). Compared with the mass sporulation and BSA, however, tetrad dissection was tedious and less amenable to the analysis of a large number of mutants. The tetrad approach was also very sensitive to errors in phenotype assignment, which can be caused by environmental variation or stochastic noise. Indeed, several mutants failed to yield a significant candidate site when using this approach (data not shown). For such mutants, some tetrads showed a clear 2:2 segregation of the fluorescence phenotype (see Figure S7A), whereas others were harder to characterize (see Figure S7B). This might be explained by the fact that each of the four sister spores was grown in a separate vial prior to phenotyping with the tetrad dissection approach, but all spores were grown together in the same vial for BSA-seq, minimizing the influence of micro-environmental factors.

**Figure 6:**
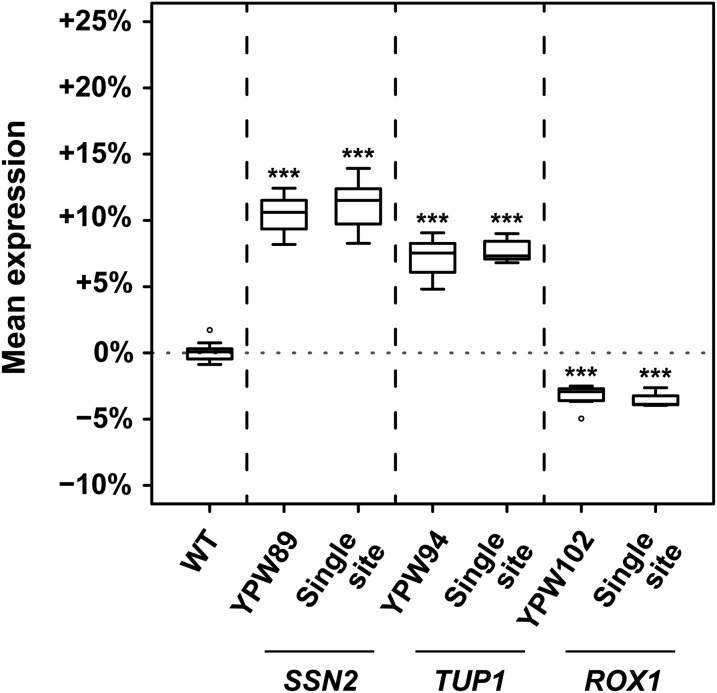
Single mutations identified by BSA-seq completely explain the mutant phenotypes. Mean expression level (measured as YFP fluorescence) for 8 replicates each of the wild-type genotype (WT); the YPW89, YPW94, and YPW102 mutants; and the three allele-replacement strains (“Single site”) for each mapped mutation (*SSN2(Q971Stop)*, *TUP1(G696D)*, and *ROX1(R12K)*) are shown. For each replicate, the median level of fluorescence of at least 5000 cells was quantified and expressed relative to mean fluorescence in the WT reference strain. Mutant genotypes and allele-replacement strains were compared to the WT strain using *t*-tests (***P < 0.001). In all three cases, the single site mutant was found to phenocopy the EMS mutant strain (*P* = 0.58 for *SSN2*, *P* = 0.23 for *TUP1*, and *P* = 0.44 for *ROX1*).

We also tested directly whether the variants identified by BSA-seq in YPW89, YPW94, and YPW102 were responsible for their mutant phenotypes by using site-directed mutagenesis to introduce each candidate mutation individually into the genetic background carrying the *P*_*TDH3*_*-YFP* transgene that was originally used for the EMS mutagenesis screen. In all three cases, the single site mutation completely recapitulated the fluorescence phenotype of the EMS-mutant from which it was identified (Figure 6). This result, combined with the absence of any other significant mutation unlinked to these causative sites (Figure 5), shows that each original mutant carried exactly one causative mutation and that this mutation could be unambiguously identified using BSA-seq despite its small phenotypic effect.

## Discussion

Using both simulated and empirical data, we describe the impact of innate properties of genetic variants and controllable experimental factors on the success of mapping single nucleotide variants using a BSA with high-throughput sequencing after a mutagenesis screen. We show how mapping success is affected by a mutation’s effect on the mean phenotype as well as its effects on phenotypic variance and fitness. By using simulations to determine optimal experimental conditions and new genetic tools to efficiently isolate large pools of informative segregants, we demonstrated the efficiency of the approach by identifying mutations in *SSN2*, *TUP1*, and *ROX1* with small effects on the expression of the *P*_*TDH3*_*-YFP* reporter gene in *S. cerevisiae*. In the sections to follow, we discuss (1) how the effects of a mutation on fitness affect mapping using BSA-seq; (2) how our findings can be applied to mapping other traits in other organisms; and (3) how our conclusions and methods can be used to study QTL underlying natural variation.

### Impact of fitness on mapping success

Previous statistical models of BSA-seq focused on the effect of segregating sites on the mean phenotype (Magwene *et al.* 2011; Edwards and Gifford 2012), but their effects on the standard deviation and relative fitness can also impact mapping success when using pooled segregant approaches in *S. cerevisiae* (Wilkening *et al.* 2013). Fitness effects could be an especially important source of discovery bias in BSA-seq data given the high proportion of random mutations showing detrimental effects on growth (Eyre-Walker and Keightley 2007; Wloch *et al.* 2001). We examined this issue computationally and found that starting with a large population of spores and selecting large pools of segregants (2 × 10^5^) was essential for achieving high mapping power when a mutation affected both the phenotype of interest and relative fitness. For example, our model predicted that selection of only 10^3^ segregants would be sufficient to map a mutation with a 3% effect on the mean and no effect on fitness, yet reducing the size of the segregant bulk in this study from 2 × 10^5^ to 10^3^ would have caused the *SSN2*(Q971*) mutation with a >10% effect on the mean to remain undetected because of its deleterious fitness effects. Because deleterious alleles tend to be purged from both bulks during growth, the power to detect a significant difference in allele frequencies between the two bulks is decreased. One solution to reduce the impact of fitness on mapping success would be to decrease the generations of growth after meiosis, but this is not always possible. For example, in our case, growth was needed for the cells to express the fluorescence phenotype as well as to increase the amount of genomic DNA available for sequencing. This latter growth phase could be shortened by using a protocol for preparing DNA libraries that requires less genomic DNA, but this usually increases noise and cost.

In the absence of fitness effects and competitive growth, alternative alleles for a site not affecting the trait of interest should be found in 50% of segregants in each bulk. Comparing the allele frequency in a single segregant bulk after phenotypic selection to a null frequency of 0.5 to detect causative mutations should be avoided, however, because any effects of a genetic variant on fitness can cause allele frequencies to deviate from this null model, increasing false positives. Rather, allele frequencies should be compared between bulks from the extremities of the phenotypic distribution. If only one tail of the distribution is amenable to phenotypic selection, for instance when selecting for drug resistance, cells that have not been subjected to phenotypic sorting but have otherwise undergone the same experimental steps as the segregant bulks should be used to define the null model (Ehrenreich *et al.* 2010; Parts *et al.* 2011).

### Applications for other traits and organisms

BSA is a powerful approach to mapping for species and traits in which large numbers of recombinant offspring can be analyzed and individuals with extreme phenotypes can be efficiently isolated. Selected bulks can now be genotyped *en masse* by high-throughput sequencing whenever a reference genome is available or can be obtained. When these conditions are met, BSA-seq can quickly identify mutations causing a mutant phenotype, even when the phenotypic effect of a mutation is very small. Our data show that the experimental design needed to most reliably and cost-effectively identify such mutations is different in each case. We encourage researchers to use the simulations and statistical models described in this study to identify experimental parameters that will maximize their own mapping success by tuning the parameters in the model to their specific system. These parameters include not only a mutation’s effect on the mean phenotype and fitness, but also its effect on the standard deviation of the phenotype. For example, we found that when the mutant phenotype has a standard deviation much larger or much smaller than the wild-type phenotype, mapping power decreases quickly with a wide range of BSA-seq experimental designs. Under these conditions, sequencing individuals from the two symmetric tails of the phenotypic distribution is not recommended and an alternative approach should be considered, such as selection of asymmetric bulks. Analysis of larger bulks should also help increase power in these cases.

When extrapolating our findings to mapping other species and traits, it is important to consider that we modeled a BSA-seq experiment in yeast including population growth between meiosis and phenotyping as well as between phenotyping and DNA sequencing. This competitive growth is not necessary when using BSA-seq in multicellular organisms such as fruit flies or nematodes. Therefore, mapping power should be much less affected by mutations that impact reproductive fitness, allowing smaller population sizes to be used. Still, the minimum effect size that can be mapped in these types of organisms will usually be larger than the minimum effect size that can be mapped in yeast, both because of the increased genome size and because of the smaller attainable bulk size. For example, if a single causative mutation is segregating in an F_2_ population, our model predicts that the power of BSA-seq to detect a mutation changing the phenotype by 5% relative to wild type is greater than 0.9 for a total population size of 10^4^, a 5% cutoff for phenotypic selection, and an average sequencing coverage of 25×. Although this is not a simple task, these parameter values can be achieved in *Drosophila melanogaster* and *Caenorhabditis elegans*, respectively, using tools such as fly cages to raise large populations of flies or worm sorters to automate phenotypic scoring and selection.

### Mapping phenotypic variation in natural populations

BSA-seq has been shown to be a powerful approach for mapping small effect QTL underlying natural variation in *S. cerevisiae* (Albert *et al.* 2014). Compared with the mutants characterized in our study, strains used for QTL mapping typically have more segregating sites and more causative loci. The large number of sites segregating in these strains leads to many linked polymorphisms, which reduces mapping resolution, but can improve the power to detect small effect QTL (Magwene *et al.* 2011; Edwards and Gifford 2012). However, the presence of multiple QTL acting in the same direction can decrease the power to detect a polymorphism of small effect compared with the case in which it segregates alone (Yang *et al.* 2013). Our work suggests that the effects of QTL on phenotypic noise and/or fitness should also be considered in future statistical models of QTL mapping via BSA-seq to avoid discovery biases.

The three genetic tools we used to increase the sensitivity of BSA-seq for finding novel mutations can also be used to study natural variation in yeast. Specifically, the dominant *RME1(ins-308A)* allele that we inserted into our mapping strain to increase meiosis rate (Deutschbauer and Davis 2005) can also be incorporated into other strains, allowing for the efficient recovery of large numbers of segregants. This is important because many strains of *S. cerevisiae*, including the commonly used S288c lab strain and its derivatives as well as some wild isolates, have low sporulation rates that limit the efficiency of BSA-seq (Gerke *et al.* 2009). The *FASTER MT* cassette (Chin *et al.* 2012) can also be inserted into other genotypes to allow for robust and efficient recovery of *MATα* spores, preventing mating among F_1_ segregants. Compared with the Yeast Magic Marker (Tong *et al.* 2001; Pan *et al.* 2004) used for a similar purpose in previous BSA studies (Albert *et al.* 2014; Wilkening *et al.* 2013; Ehrenreich *et al.* 2010), *FASTER MT* requires less genetic manipulation of the mapping strain(s), reduces biases caused by diploids in the sorted haploid cultures (Gerstein and Otto 2011; Wilkening *et al.* 2013), and limits the impact of competitive growth on mapping power by allowing *MATα* cells to be sorted immediately after spore isolation. Finally, if the phenotype of interest can be coupled to a fluorescent reporter gene, FACS can be used for high-throughput phenotyping and selection. Other easy-to-score phenotypes should also be well suited for genetic mapping using BSA-seq.

In conclusion, this study provides a methodological framework for efficiently mapping genetic variants with small effects, illustrates the importance of considering the fitness effects of causative variants when using BSA-seq in microorganisms such as yeast, and describes the use of experimental tools that can reduce the bias against detection of variants with small effects by allowing very large populations of phenotypically divergent individuals to be collected and analyzed.

## Supplementary Material

Supporting Information

## References

[bib1] Albert F. W. Treusch S. Shockley A. H. Bloom J. S. Kruglyak L., 2014 Genetics of single-cell protein abundance variation in large yeast populations. Nature 506: 494–497. 24402228 10.1038/nature12904 PMC4285441

[bib2] Bastide H. Betancourt A. Nolte V. Tobler R. Stöbe P., 2013 A genome-wide, fine-scale map of natural pigmentation variation in *Drosophila melanogaster*. PLoS Genet. 9: e1003534. 23754958 10.1371/journal.pgen.1003534 PMC3674992

[bib3] Birkeland S. R. Jin N. Ozdemir A. C. Lyons R. H. Weisman L. S., 2010 Discovery of mutations in *Saccharomyces cerevisiae* by pooled linkage analysis and whole-genome sequencing. Genetics 186: 1127–1137. 20923977 10.1534/genetics.110.123232 PMC2998298

[bib4] Brauer M. J. Christianson C. M. Pai D. A. Dunham M. J., 2006 Mapping novel traits by array-assisted bulk segregant analysis in *Saccharomyces cerevisiae*. Genetics 173: 1813–1816. 16624899 10.1534/genetics.106.057927 PMC1526703

[bib5] Breslow D. K. Cameron D. M. Collins S. R. Schuldiner M. Stewart-Ornstein J., 2008 A comprehensive strategy enabling high-resolution functional analysis of the yeast genome. Nat. Methods 5: 711–718. 18622397 10.1038/nmeth.1234 PMC2756093

[bib6] Chin B. L. Frizzell M. a. Timberlake W. E. Fink G. R., 2012 FASTER MT: isolation of pure populations of a and α ascospores from *Saccharomyces cerevisiae*. G3 (Bethesda) 2: 449–452. 22540036 10.1534/g3.111.001826 PMC3337473

[bib7] Deutschbauer A. M. Davis R. W., 2005 Quantitative trait loci mapped to single-nucleotide resolution in yeast. Nat. Genet. 37: 1333–1340. 16273108 10.1038/ng1674

[bib8] Deutschbauer A. M. Jaramillo D. F. Proctor M. Kumm J. Hillenmeyer M. E., 2005 Mechanisms of haploinsufficiency revealed by genome-wide profiling in yeast. Genetics 169: 1915–1925. 15716499 10.1534/genetics.104.036871 PMC1449596

[bib9] Edwards M. D. Gifford D. K., 2012 High-resolution genetic mapping with pooled sequencing. BMC Bioinformatics 13(Suppl 6): S8. 22537047 10.1186/1471-2105-13-S6-S8 PMC3358661

[bib10] Ehrenreich I. M. Torabi N. Jia Y. Kent J. Martis S., 2010 Dissection of genetically complex traits with extremely large pools of yeast segregants. Nature 464: 1039–1042. 20393561 10.1038/nature08923 PMC2862354

[bib11] Ehrenreich I. M. Bloom J. Torabi N. Wang X. Jia Y., 2012 Genetic architecture of highly complex chemical resistance traits across four yeast strains. PLoS Genet. 8: e1002570. 22438822 10.1371/journal.pgen.1002570 PMC3305394

[bib12] Eyre-Walker A. Keightley P. D., 2007 The distribution of fitness effects of new mutations. Nat. Rev. Genet. 8: 610–618. 17637733 10.1038/nrg2146

[bib13] Fellows I., 2012 Deducer: a data analysis GUI for R. J. Stat. Softw. 49: 1–15.

[bib14] Gerke J. Lorenz K. Cohen B., 2009 Genetic interactions between transcription factors cause natural variation in yeast. Science 323: 2007–2010. 10.1126/science.1166426 PMC4984536 19164747

[bib15] Gerstein A. C. Otto S. P., 2011 Cryptic fitness advantage: diploids invade haploid populations despite lacking any apparent advantage as measured by standard fitness assays. PLoS ONE 6: e26599. 22174734 10.1371/journal.pone.0026599 PMC3235103

[bib16] Granek J. A. Murray D. Kayikçi O. Magwene P. M., 2012 The genetic architecture of biofilm formation in a clinical isolate of *Saccharomyces cerevisiae*. Genetics 193: 587–600. 23172850 10.1534/genetics.112.142067 PMC3567746

[bib17] Gruber J. D. Vogel K. Kalay G. Wittkopp P. J., 2012 Contrasting properties of gene-specific regulatory, coding, and copy number mutations in *Saccharomyces cerevisiae*: frequency, effects, and dominance. PLoS Genet. 8: e1002497. 22346762 10.1371/journal.pgen.1002497 PMC3276545

[bib18] Hanlon S. E. Rizzo J. M. Tatomer D. C. Lieb J. D. Buck M. J., 2011 The stress response factors Yap6, Cin5, Phd1, and Skn7 direct targeting of the conserved co-repressor Tup1-Ssn6 in *S. cerevisiae*. PLoS ONE 6: e19060. 21552514 10.1371/journal.pone.0019060 PMC3084262

[bib19] Harbison C. T. Gordon D. B. Lee T. I. Rinaldi N. J. Macisaac K. D., 2004 Transcriptional regulatory code of a eukaryotic genome. Nature 431: 99–104. 15343339 10.1038/nature02800 PMC3006441

[bib20] Koboldt D. C. Chen K. Wylie T. Larson D. E. McLellan M. D., 2009 VarScan: variant detection in massively parallel sequencing of individual and pooled samples. Bioinformatics 25: 2283–2285. 19542151 10.1093/bioinformatics/btp373 PMC2734323

[bib21] Koboldt D. C. Zhang Q. Larson D. E. Shen D. McLellan M. D., 2012 VarScan 2: Somatic mutation and copy number alteration discovery in cancer by exome sequencing. Genome Res. 22: 568–576. 22300766 10.1101/gr.129684.111 PMC3290792

[bib22] Kofler R. Pandey R. V. Schlötterer C., 2011 PoPoolation2: identifying differentiation between populations using sequencing of pooled DNA samples (Pool-Seq). Bioinformatics 27: 3435–3436. 22025480 10.1093/bioinformatics/btr589 PMC3232374

[bib23] Kumar P. Henikoff S. Ng P. C., 2009 Predicting the effects of coding non-synonymous variants on protein function using the SIFT algorithm. Nat. Protoc. 4: 1073–1081. 19561590 10.1038/nprot.2009.86

[bib24] Langmead B. Salzberg S. L., 2012 Fast gapped-read alignment with Bowtie 2. Nat. Methods 9: 357–359. 22388286 10.1038/nmeth.1923 PMC3322381

[bib25] Larochelle M. Drouin S. Robert F. Turcotte B., 2006 Oxidative stress-activated zinc cluster protein Stb5 has dual activator/repressor functions required for pentose phosphate pathway regulation and NADPH production. Mol. Cell. Biol. 26: 6690–6701. 16914749 10.1128/MCB.02450-05 PMC1592823

[bib26] Li H. Handsaker B. Wysoker A. Fennell T. Ruan J., 2009 The sequence Alignment/Map format and SAMtools. Bioinformatics 25: 2078–2079. 19505943 10.1093/bioinformatics/btp352 PMC2723002

[bib27] Liti G. Louis E. J., 2012 Advances in quantitative trait analysis in yeast. PLoS Genet. 8: e1002912. 22916041 10.1371/journal.pgen.1002912 PMC3420948

[bib28] Magwene P. M. Willis J. H. Kelly J. K., 2011 The statistics of bulk segregant analysis using next generation sequencing. PLOS Comput. Biol. 7: e1002255. 22072954 10.1371/journal.pcbi.1002255 PMC3207950

[bib29] Mennella T. A. Klinkenberg L. G. Zitomer R. S., 2003 Recruitment of Tup1-Ssn6 by yeast hypoxic genes and chromatin-independent exclusion of TATA binding protein. Eukaryot. Cell 2: 1288–1303. 14665463 10.1128/EC.2.6.1288-1303.2003 PMC326644

[bib30] Michelmore R. W. Paran I. Kesseli R. V., 1991 Identification of markers linked to disease-resistance genes by bulked segregant analysis: a rapid method to detect markers in specific genomic regions by using segregating populations. Proc. Natl. Acad. Sci. USA 88: 9828–9832. 1682921 10.1073/pnas.88.21.9828 PMC52814

[bib31] Pan X. Yuan D. S. Xiang D. Wang X. Sookhai-Mahadeo S., 2004 A robust toolkit for functional profiling of the yeast genome. Mol. Cell 16: 487–496. 15525520 10.1016/j.molcel.2004.09.035

[bib32] Parts L. Cubillos F. A. Warringer J. Jain K. Salinas F., 2011 Revealing the genetic structure of a trait by sequencing a population under selection. Genome Res. 21: 1131–1138. 21422276 10.1101/gr.116731.110 PMC3129255

[bib33] Pomraning K. R. Smith K. M. Freitag M., 2011 Bulk segregant analysis followed by high-throughput sequencing reveals the *Neurospora* cell cycle gene, *ndc-1*, to be allelic with the gene for ornithine decarboxylase, *spe-1*. Eukaryot. Cell 10: 724–733. 21515825 10.1128/EC.00016-11 PMC3127673

[bib34] R Development Core Team, 2013 R: A Language and Environment for Statistical Computing. R Foundation for Statistical Computing, Vienna, Austria.

[bib35] Rohland N. Reich D., 2012 Cost-effective, high-throughput DNA sequencing libraries for multiplexed target capture. Genome Res. 22: 939–946. 22267522 10.1101/gr.128124.111 PMC3337438

[bib36] Song W. Treich I. Qian N. Kuchin S. Carlson M., 1996 *SSN* genes that affect transcriptional repression in *Saccharomyces cerevisiae* encode SIN4, ROX3, and SRB proteins associated with RNA polymerase II. Mol. Cell. Biol. 16: 115–120. 8524287 10.1128/mcb.16.1.115 PMC230984

[bib37] Storici F. Resnick M. A., 2006 The *delitto perfetto* approach to *in vivo* site-directed mutagenesis and chromosome rearrangements with synthetic oligonucleotides in yeast. Methods Enzymol. 409: 329–345. 16793410 10.1016/S0076-6879(05)09019-1

[bib38] Swinnen S. Schaerlaekens K. Pais T. Claesen J. Hubmann G., 2012 Identification of novel causative genes determining the complex trait of high ethanol tolerance in yeast using pooled-segregant whole-genome sequence analysis. Genome Res. 22: 975–984. 22399573 10.1101/gr.131698.111 PMC3337442

[bib39] Tong A. H. Evangelista M. Parsons A. B. Xu H. Bader G. D., 2001 Systematic genetic analysis with ordered arrays of yeast deletion mutants. Science 294: 2364–2368. 11743205 10.1126/science.1065810

[bib40] Van Leeuwen T. Demaeght P. Osborne E. J. Dermauw W. Gohlke S., 2012 Population bulk segregant mapping uncovers resistance mutations and the mode of action of a chitin synthesis inhibitor in arthropods. Proc. Natl. Acad. Sci. USA 109: 4407–4412. 22393009 10.1073/pnas.1200068109 PMC3311382

[bib41] Wenger J. W. Schwartz K. Sherlock G., 2010 Bulk segregant analysis by high-throughput sequencing reveals a novel xylose utilization gene from *Saccharomyces cerevisiae*. PLoS Genet. 6: e1000942. 20485559 10.1371/journal.pgen.1000942 PMC2869308

[bib42] Wicks S. R. Yeh R. T. Gish W. R. Waterston R. H. Plasterk R. H., 2001 Rapid gene mapping in *Caenorhabditis elegans* using a high density polymorphism map. Nat. Genet. 28: 160–164. 11381264 10.1038/88878

[bib43] Wilkening S. Lin G. Fritsch E. S. Tekkedil M. M. Anders S., 2014 An evaluation of high-throughput approaches to QTL mapping in *Saccharomyces cerevisiae*. Genetics 196: 853–865. 24374355 10.1534/genetics.113.160291 PMC3948811

[bib44] Wloch D. M. Szafraniec K. Borts R. H. Korona R., 2001 Direct estimate of the mutation rate and the distribution of fitness effects in the yeast *Saccharomyces cerevisiae*. Genetics 159: 441–452. 11606524 10.1093/genetics/159.2.441 PMC1461830

[bib45] Xia Y. Won S. Du X. Lin P. Ross C., 2010 Bulk segregation mapping of mutations in closely related strains of mice. Genetics 186: 1139–1146. 20923982 10.1534/genetics.110.121160 PMC2998299

[bib46] Yang Y. Foulquié-Moreno M. R. Clement L. Erdei E. Tanghe A., 2013 QTL analysis of high thermotolerance with superior and downgraded parental yeast strains reveals new minor QTLs and converges on novel causative alleles involved in RNA processing. PLoS Genet. 9: e1003693. 23966873 10.1371/journal.pgen.1003693 PMC3744412

