# Supplementary material for: Mapping Small Effect Mutations in Saccharomyces cerevisiae: Impacts of Experimental Design and Mutational Properties
Source: G3 (Bethesda). 2014 Apr 29;4(7):1205–16. doi: 10.1534/g3.114.011783 (PMC4455770; doi:10.1534/g3.114.011783)
Supplement: Supporting Information [file supp_4_7_1205__index.html]

Mapping Small Effect Mutations in Saccharomyces cerevisiae: Impacts of Experimental Design and Mutational Properties — Supporting Information 

# Mapping Small Effect Mutations in *Saccharomyces cerevisiae*: Impacts of Experimental Design and Mutational Properties

## Supporting Information for Duveau *et al.*, 2014

**Files in this Data Supplement:**

- Supporting Information - Figures S1-S10 and Files S1-S4 (PDF, 681 KB)
- Figure S1 - Example of phenotypic distribution after the deterministic phase of our simulation establishing the segregant pools. (PDF, 174 KB)
- Figure S2 - Phenotypic effects of the *trans*-regulatory mutants described in Gruber *et al.* (2012). (PDF, 168 KB)
- Figure S3 - Statistical power to detect a difference in the frequency of a neutral mutation (mean effect = 0%) between bulks depending on average depth of coverage and population size. (PDF, 149 KB)
- Figure S4 - Phenotypic distributions after the deterministic phase of the simulations. (PDF, 205 KB)
- Figure S5 - FACS gating used to collect low and high fluorescence pools. (PDF, 574 KB)
- Figure S6 - Measures of allele frequency derived from high-throughput sequencing were highly correlated with measures derived from pyrosequencing. (PDF, 177 KB)
- Figure S7 - Spore phenotypes assayed after tetrad dissection show phenotypic differences between spores with and without the causative site in some, but not all, cases. (PDF, 605 KB)
- Figure S8 - Tetrad-based mapping identifies the same candidate sites as BSA-seq. (PDF, 192 KB)
- Figure S9 - Sequencing coverage for each bulk shows two peaks. (PDF, 226 KB)
- Figure S10 - Poor mapping rather than fluctuations in sequencing coverage due to random sampling are responsible for most mapping blind spots. (PDF, 539 KB)
- File S1 - Extended Materials and Methods (PDF, 246 KB)
- File S4 - Robustness of the BSA-seq approach. (PDF, 164 KB)
- File S2 - R script for power analyses (.r, 49 KB)
- File S3 - Description of pyrosequencing assays for quantitative genotyping of seven genetic variants in the segregant bulks. (.xls, 28 KB)
