# Supplementary material for: Mapping Small Effect Mutations in Saccharomyces cerevisiae: Impacts of Experimental Design and Mutational Properties
Source: G3 (Bethesda). 2014 Apr 29;4(7):1205–16. doi: 10.1534/g3.114.011783 (PMC4455770; doi:10.1534/g3.114.011783)
Supplement: Supporting Information [file supp_g3.114.011783_011783SI.pdf]

G3·Genes | Genomes | Genetics

Mapping small effect mutations in *Saccharomyces cerevisiae*: impacts of experimental design and mutational properties

Fabien Duveau[*,1], Brian P.H. Metzger[*], Jonathan D. Gruber[*], Katya Mack[*], Natasha Sood[*], Tiffany E. Brooks[*], Patricia J. Wittkopp[*,§,†,2]

[*]Department of Ecology and Evolutionary Biology, University of Michigan, Ann Arbor, Michigan 48109-1048, [§]Department of Molecular, Cellular, & Developmental Biology, University of Michigan, Ann Arbor, Michigan 48109-1048, [†]Center for Computational Medicine and Bioinformatics, University of Michigan Medical School, Ann Arbor, Michigan 48109-2218

[1]Corresponding author: Fabien Duveau, Dept. of Ecology and Evolutionary Biology, 1061 Kraus Nat. Sci. Bldg., 830 North University, University of Michigan, Ann Arbor, Michigan 48109-1048, Tel: 734-647-5483, e-mail: fduveau@umich.edu

[2]Corresponding author: Patricia J. Wittkopp, Dept. of Ecology and Evolutionary Biology, 1059 Kraus Nat. Sci. Bldg., 830 North University, University of Michigan, Ann Arbor, Michigan 48109-1048, Tel: 734-763-1548, e-mail: wittkopp@umich.edu

DOI: 10.1534/g3.114.011783

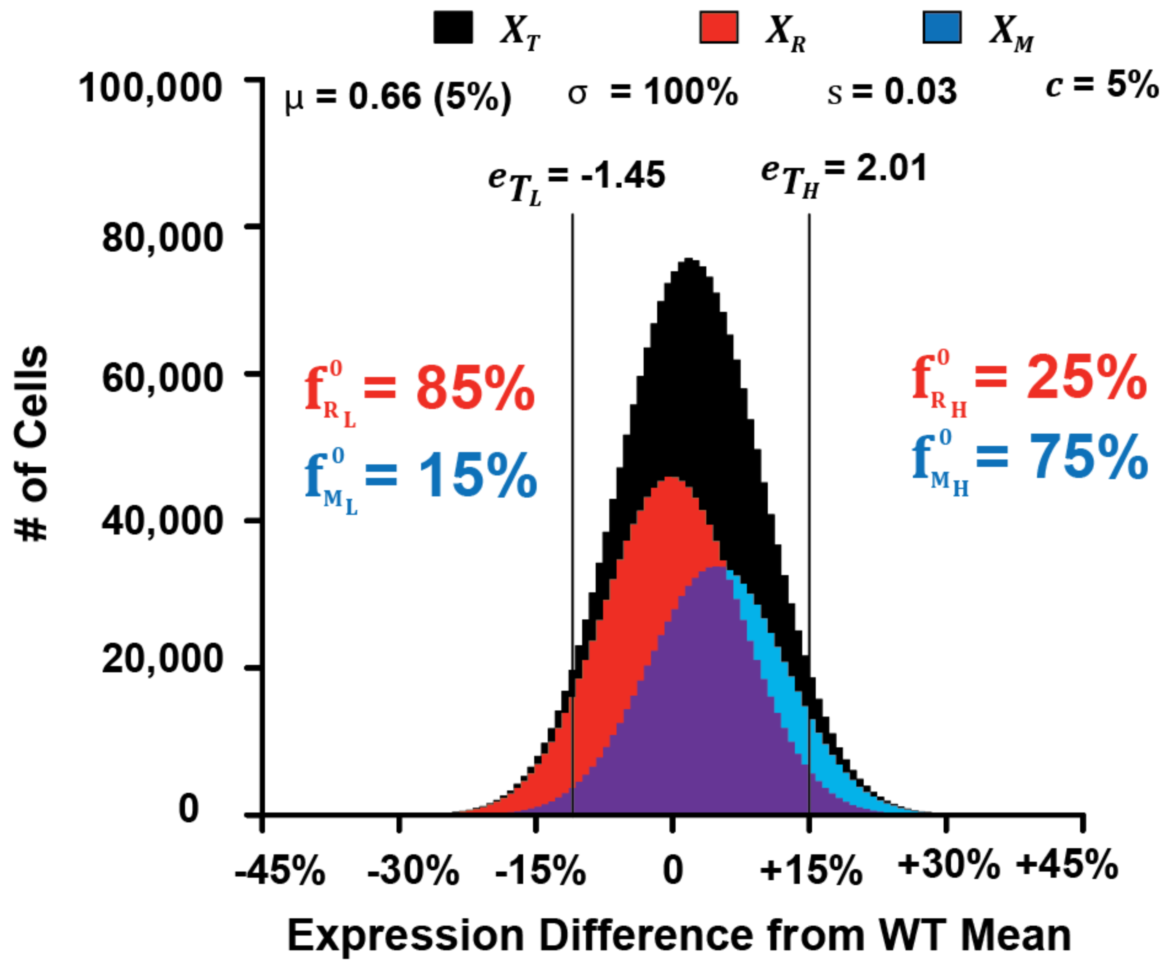

Figure S1 Example of phenotypic distribution after the deterministic phase of our simulation establishing the segregant pools. For a full description of the model used to generate these distributions, see File S1. The phenotypic distribution for all cells in the population (X_T) is shown in black, whereas the phenotypic distributions for cells carrying the reference (X_R) and mutant (X_M) alleles of the causative site are shown in red and blue, respectively. Black lines show the 5th and 95th percentiles of the phenotypic distribution for all cells, which correspond to the thresholds used for sorting with a 5% cutoff for the high and low bulks. The frequency of the reference allele (f_R) and the frequency of the mutant allele (f_M) are shown for both the low (L) and high (H) bulks. Results are shown for a causative mutation that changes the mean (μ) by 5%, has no effect on the phenotypic standard deviation (σ), and has a selection coefficient (s) of 0.03, when the selected bulks are obtained using a 5% cutoff (c).

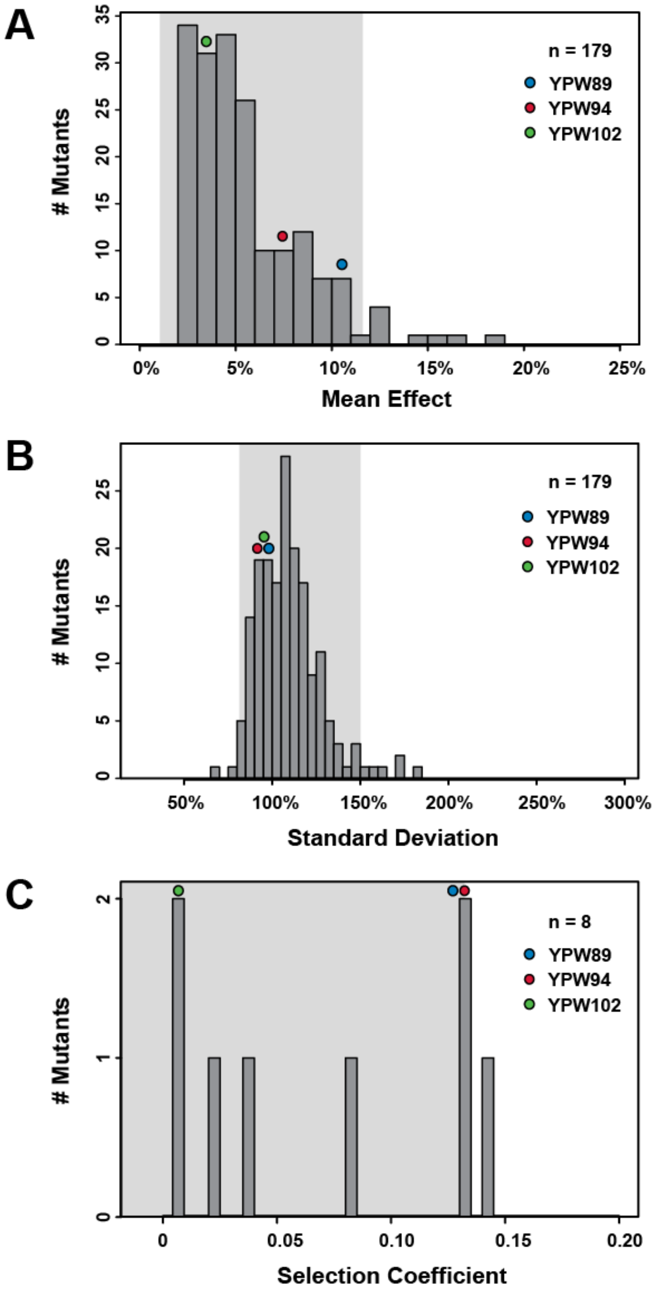

Figure S2 Phenotypic effects of the *trans*-regulatory mutants described in Gruber *et al.* (2012). Absolute values of effects on mean expression level (A) and standard deviation of fluorescence (B) relative to wild type are shown for the full set of 179 *trans*-regulatory mutants. (C) Selection coefficients for 8 randomly selected mutants, including the three mutants used for mapping in this study (YPW89, YPW94 and YPW102), are shown. Shaded regions show confidence intervals excluding the 10% most extreme mutants and correspond to the shaded regions in Figure 1 and Figure 2. Colored dots indicate the parameter values for the three mutants analyzed in this study.

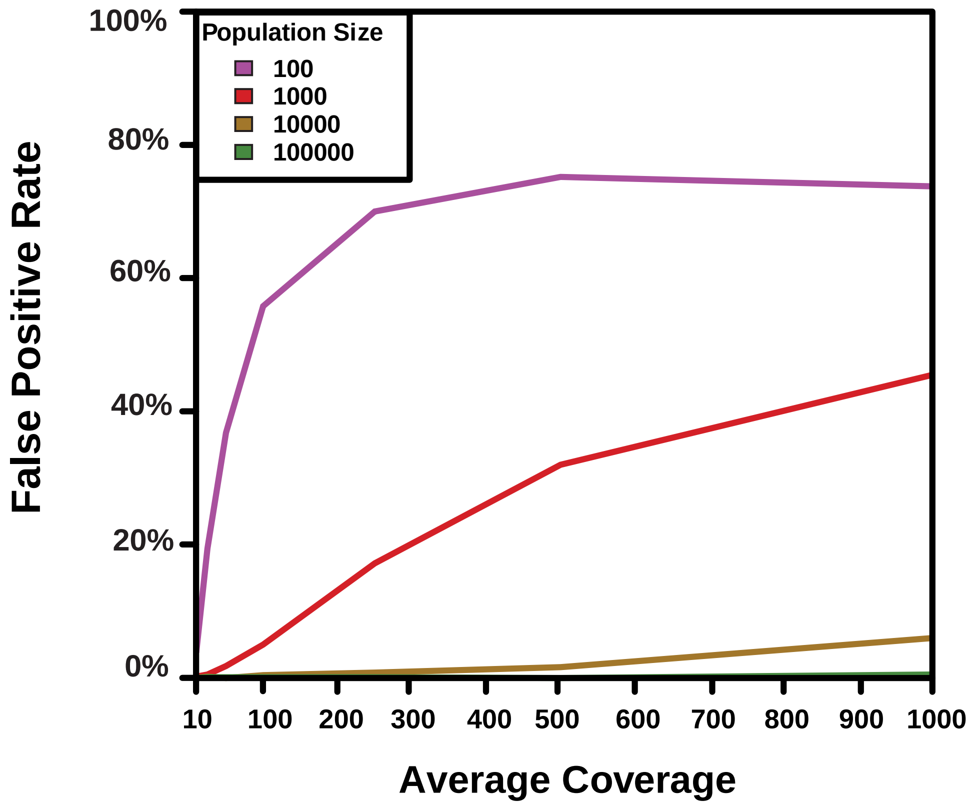

Figure S3 Statistical power to detect a difference in the frequency of a neutral mutation (mean effect = 0%) between bulks depending on average depth of coverage and population size. This power corresponds to the false discovery rate.

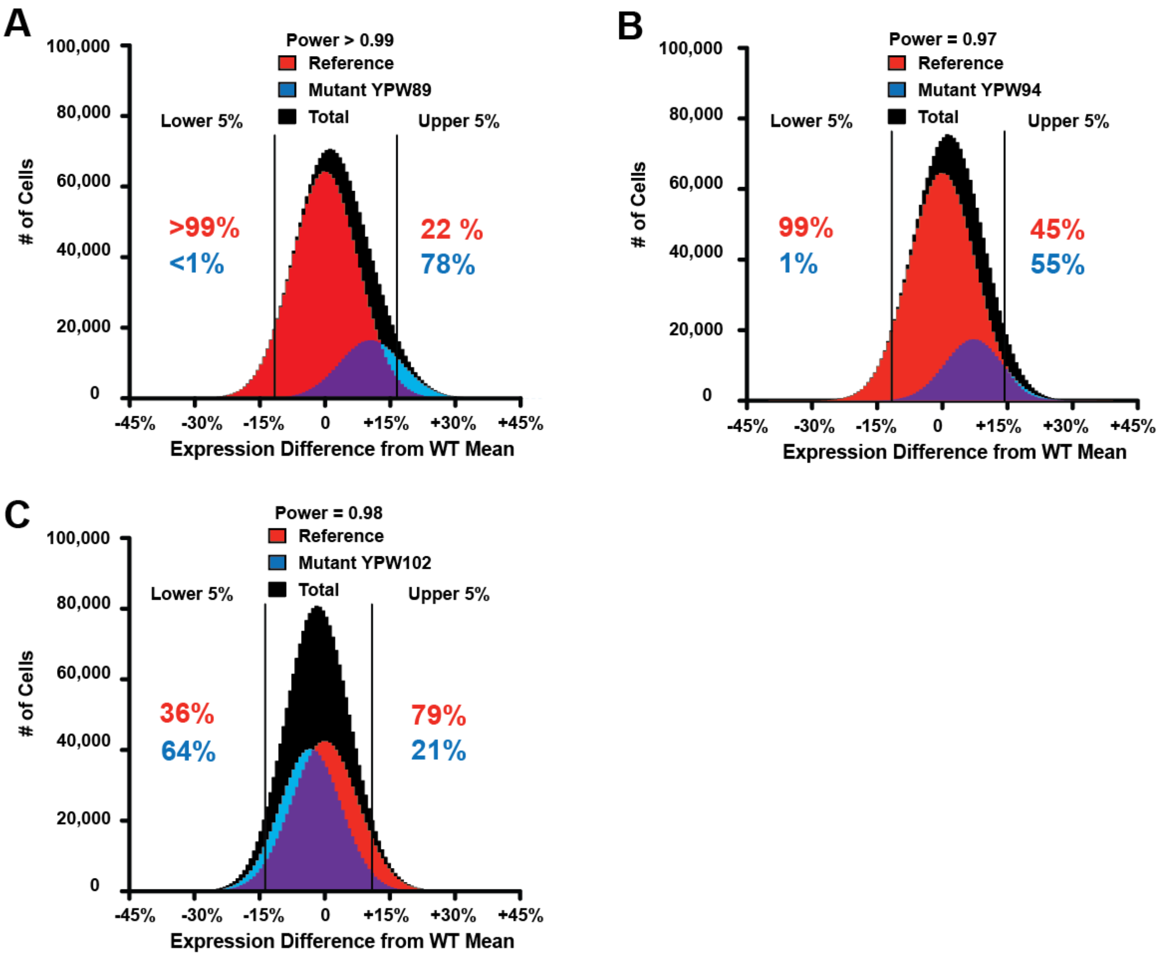

Figure S4 Phenotypic distributions after the deterministic phase of the simulations. Results are shown for mutant YPW89 (A), YPW94 (B), and YPW102 (C). Parameters used for the mean, standard deviation, and selection coefficient of the mutant causal allele were estimated from fluorescence and fitness phenotypes of the mutant strains (Table 1). Black: Total population distribution; Red: Reference allele containing population distribution; Blue: Mutant allele containing population distribution. Black lines show the 5% and 95% cutoffs on the total (black) distribution. Numbers in red indicate the frequency of the reference allele in the two bulks while numbers in blue indicate the frequency of the mutant allele in the two bulks. The power to detect a significant difference ($P < 0.001$) in mutation frequency between lower and higher tails in a G-test given an average sequencing coverage of 75 is shown above each plot.

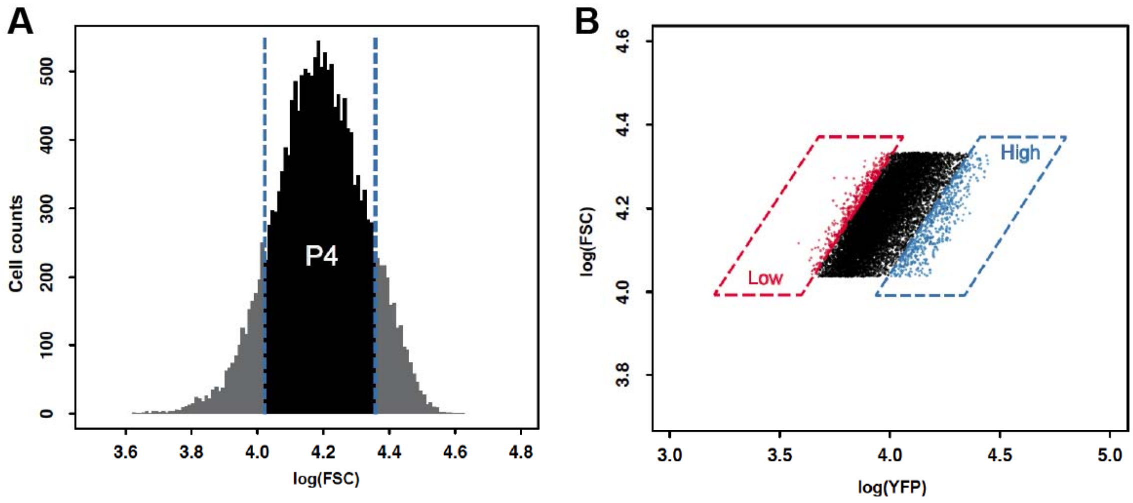

C

Population	#Events	%Parent	%Total	FSC Median	YFP Median
All Events	50,000	----	100.0	25,293	13,873
P4	12,155	86.5	24.3	14,916	9,006
Low Bulk	391	3.3	0.8	14,266	6,000
High Bulk	385	3.2	0.8	14,249	17,909

Figure S5 FACS gating used to collect low and high fluorescence pools. Data shown is from the analysis of mutant YPW102. Gating based on the relationship between FSC-A and FSC-H was used to remove cell doublets (not shown). (A) Gating based on FSC-A, which is a proxy for cell size, was then used to exclude the smallest ~8% and the largest ~8% of events. (B) Finally, low and high bulks were selected based on fluorescence level (log(YFP)) and cell size (log(FSC-A)). Careful attention was paid to select bulks with different fluorescence levels, but similar cell sizes. (C) Changes in event number (cells) resulting from the gates shown in panels (A) and (B).

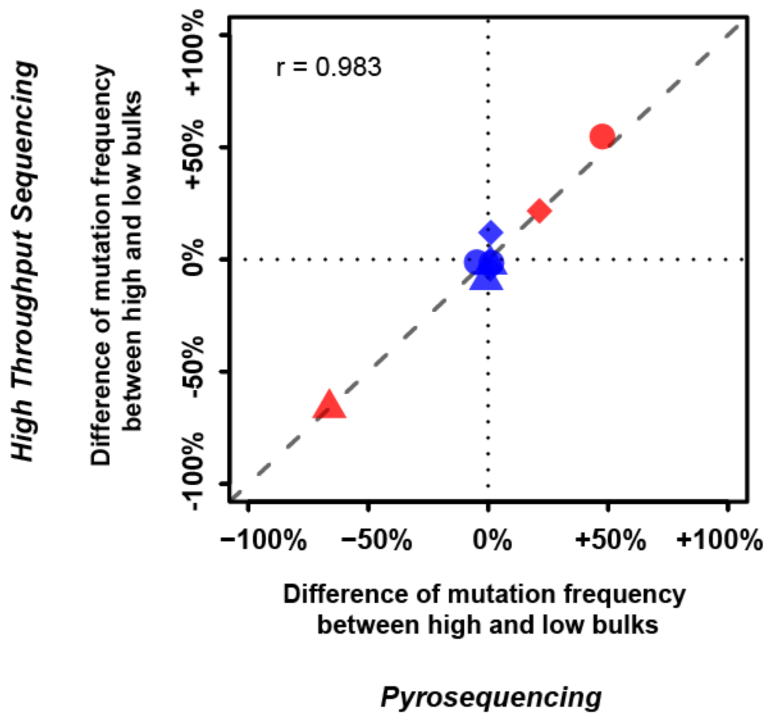

Figure S6 Measures of allele frequency derived from high-throughput sequencing were highly correlated with measures derived from pyrosequencing. For each mutant, pyrosequencing assays were developed for quantitative genotyping of two phenotypically neutral sites (blue) as well as for the site with the highest significance of association with the fluorescence phenotype (red). The plot shows the difference in mutant allele frequency between the high fluorescence and low fluorescence bulks for each site as determined by pyrosequencing (x-axis) or whole genome sequencing (y-axis). Different shapes represent sites analyzed in different mutants (diamond: YPW89, circle: YPW94, triangle: YPW102).

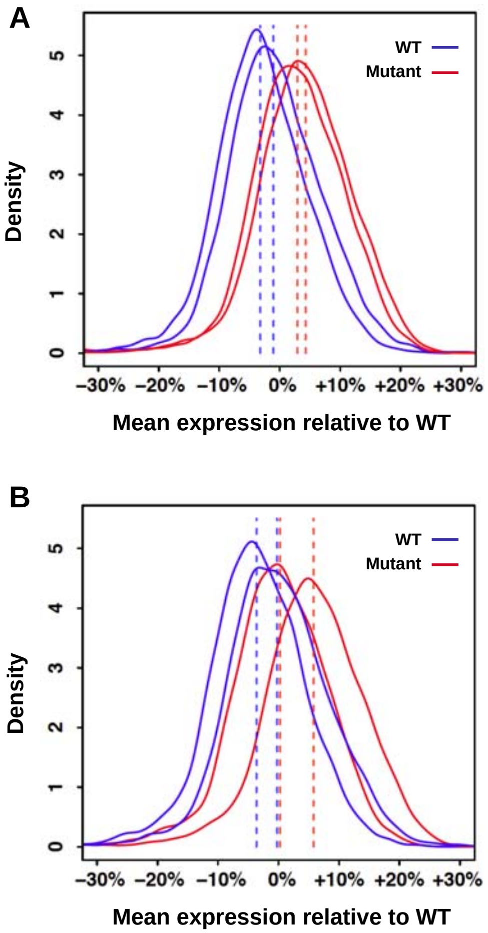

Figure S7 Spore phenotypes assayed after tetrad dissection show phenotypic differences between spores with and without the causative site in some, but not all, cases. Segregation of the YFP phenotype in two tetrads derived from mutant YPW54 are shown. (A) Tetrad showing a clear 2:2 segregation of fluorescence level. (B) Tetrad for which mutant and wild-type progeny are hard to distinguish based on fluorescence, potentially leading to incorrect assignment to a phenotypic pool when assembling mutant and reference pools for mapping. Blue and red solid lines show distributions of fluorescence for populations derived from spores assumed to harbor wild type and mutant alleles of the causative site, respectively. Dotted lines indicate the median fluorescence level for each of the wild-type (blue) and mutant (red) populations.

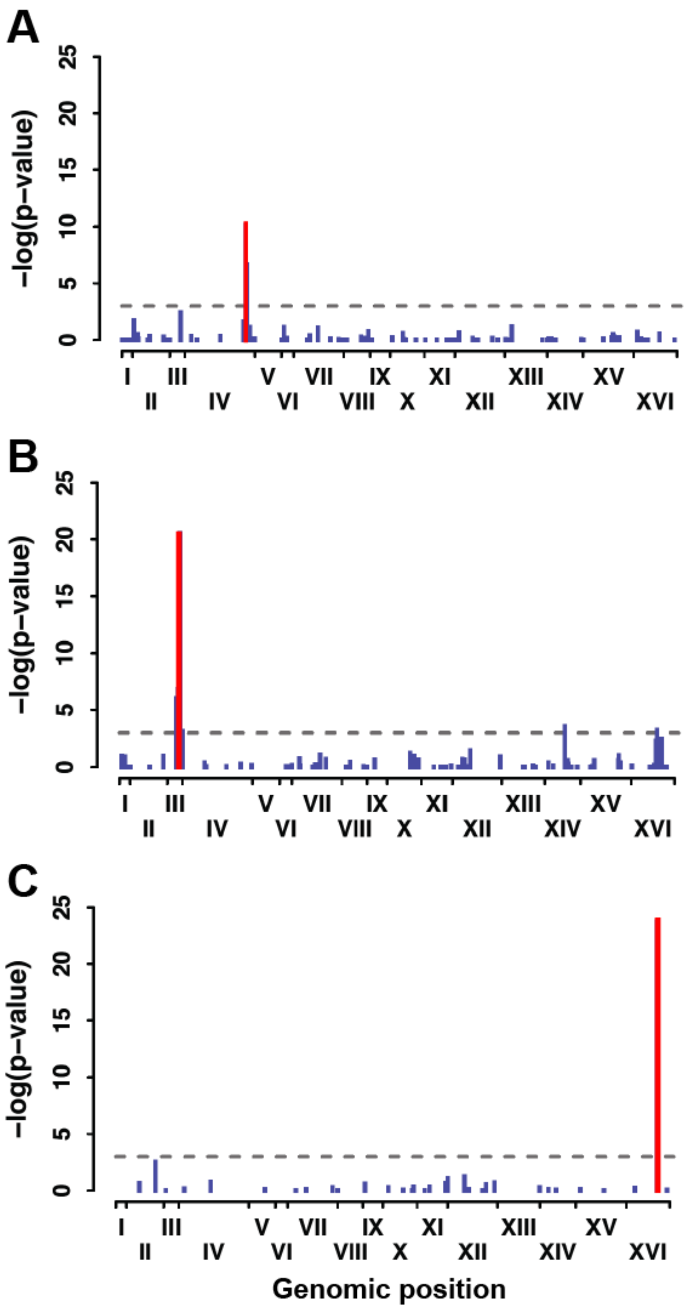

Figure S8 Tetrad-based mapping identifies the same candidate sites as BSA-seq. Mapping results from a more traditional mapping approach based on tetrad dissection are shown for mutant YPW89 (A), YPW94 (B) and YPW102 (C). Colored bars represent individual EMS-induced mutations with their genomic position represented on x-axis and significance of the difference in allele frequency between low fluorescence and high fluorescence bulks represented on y-axis (negative logarithm of *P*-value from G-test). For each mutant, the most significant site identified by BSA-seq is shown in red. Horizontal dotted lines represent a significance threshold of α=0.001.

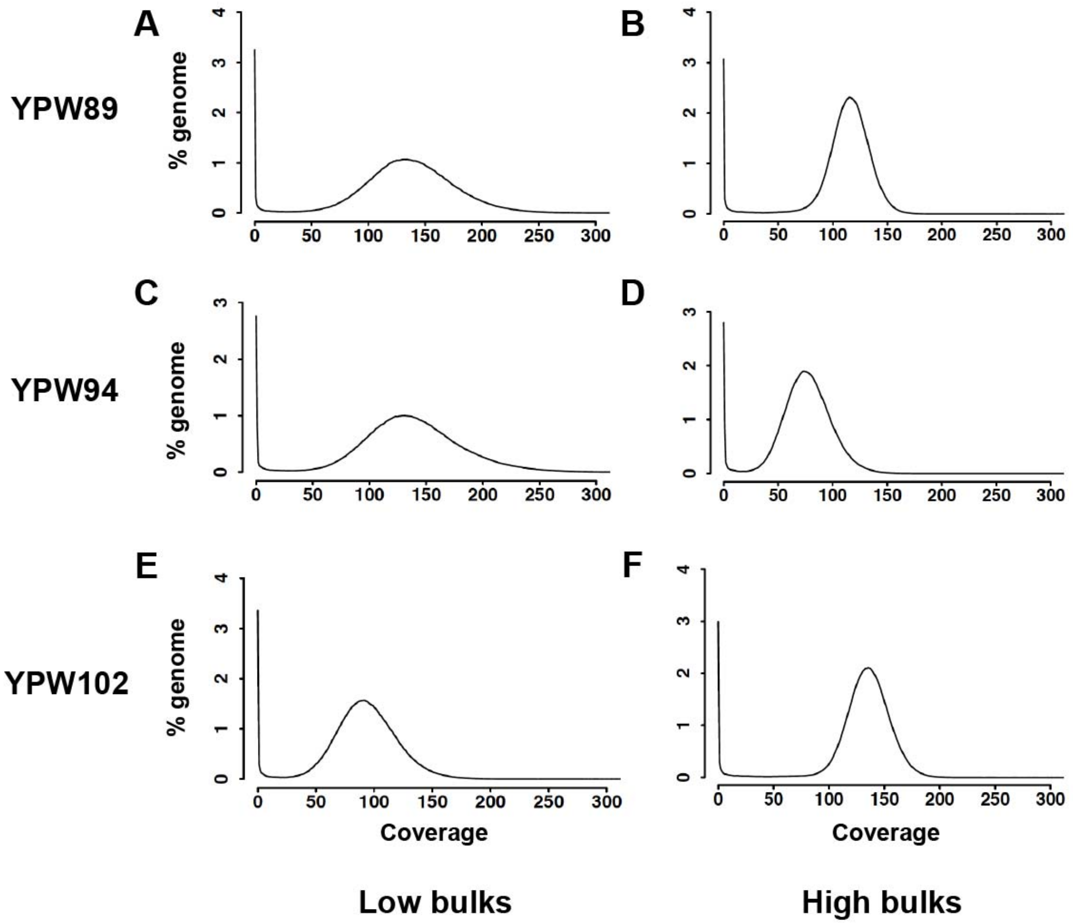

Figure S9 Sequencing coverage for each bulk shows two peaks. Distributions of sequencing coverage across reference genome are shown for low (A,C,E) and high (B,D,F) bulks obtained from mutants YPW89 (A,B), YPW94 (C,D) and YPW102 (E,F). Note the peak at 0, which indicates sites with no overlapping sequencing reads, in addition to the peak near the average coverage for each sample.

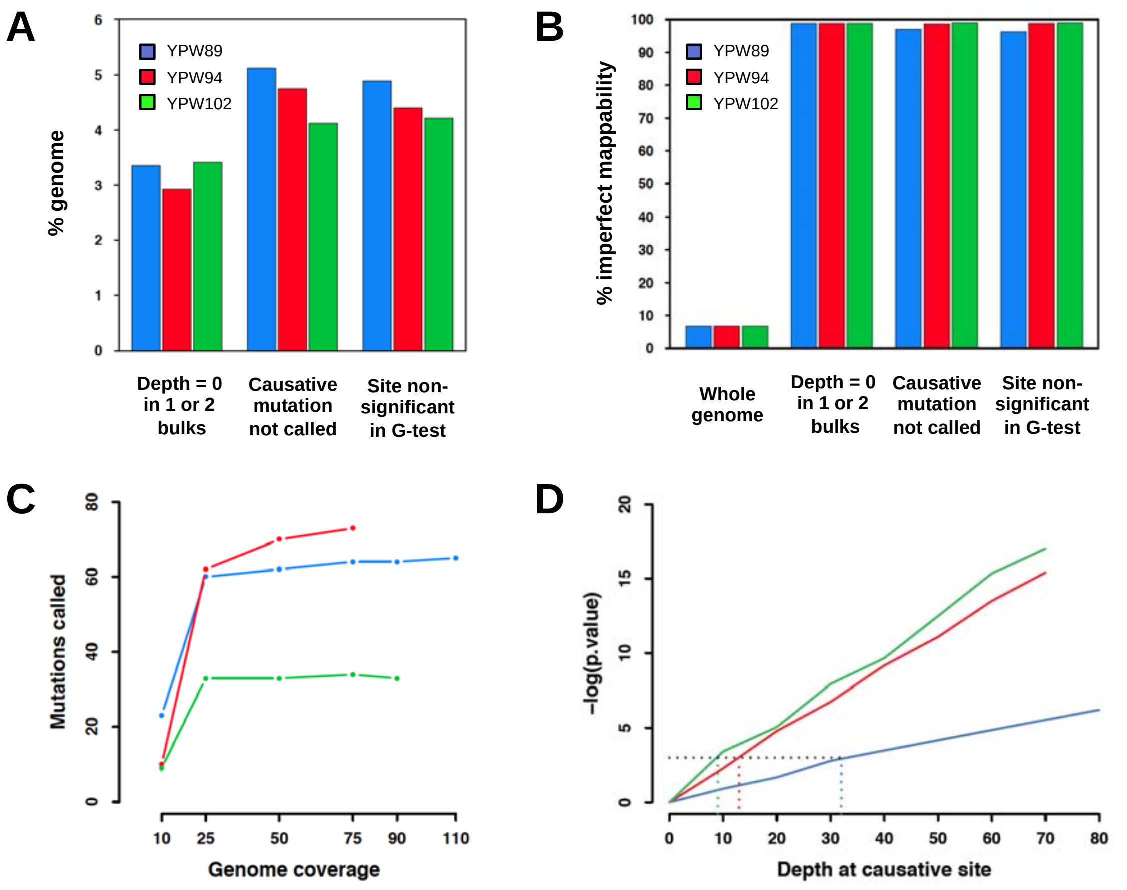

Figure S10 Poor mapping rather than fluctuations in sequencing coverage due to random sampling are responsible for most mapping blind spots. (A) For each mutant, the fraction of the genome with insufficient coverage to detect a putative causative mutation because zero reads were observed in one or both bulks (left), the causative mutation was not called as a sequence variant (middle), or the number of reads mapped was insufficient to generate a significant G-test (right) is shown. (B) Proportion of sites with imperfect mappability across the whole genome and for each genomic class considered in (A) are shown. The vast majority of sites for which a putative mutation could not be detected also showed poor mappability. (C) Robustness of the total number of mutations called to variation in sequencing depth is shown. For each mutant, SNPs were called after subsampling mapped reads to a sequencing depth of 90x, 75x, 50x, 25x and 10x in low and high bulks. Mutants are color coded as in panel (A). (D) Significance of the causative site depending on its coverage is shown. For a constant mutation frequency at the causative site, the total number of alleles was decreased from 90 to 0 (x-axis) and the *P*-value of the G-test was computed (y-axis). Mutants are color coded as in panel (A). Dotted lines highlight the threshold of coverage below which *P*-values were considered non-significant (*P*>0.001).

File S1

Extended Materials and Methods

Power analyses

Modeling exact allele frequencies in bulks: The goal of the deterministic step of the model was to calculate the frequency of mutant and reference alleles expected in each phenotypically divergent bulk of cells depending on total population size used for sorting (n), phenotypic selection cutoff used for isolating bulks (c), generations of growth after meiosis (g), mutation effect on mean expression (μ), mutation effect on standard deviation in expression (σ), and selection coefficient for the mutation (s). We modeled the total population distribution with respect to expression, X_T, as a mixture distribution of two populations, X_R and X_M, where X_R is the population carrying the reference allele at the causative locus and X_M is the population carrying the mutant allele at the causative locus, and tracked each population separately. Each population was assumed to follow a normal distribution with respect to expression:

(1) $X_R \sim N(0,1)$

(2) $X_M \sim N(\mu, \sigma^2)$

We represent the mean effect of a causal mutation, μ, relative to the standard deviation of the reference strain such that an increase of μ by 1 is equivalent to a shift in mean expression by one standard deviation (an approximately 7.5% change in expression in our data). Mutations were assumed not to influence sporulation efficiency or spore survival and X_R and X_M were started at equal frequencies. Populations were allowed to grow deterministically assuming a selection coefficient for the mutant causative allele of s. The reference allele was assumed to have fitness of 1 and after g generations the frequency of the mutant population in the population was:

(3) $f^0_{M_W} = \frac{(1-s)^g}{(1-s)^g+1}$

where W indicates the whole mutant or reference population prior to selection of phenotypic bulks (see figure S1 for diagram). The reference allele frequency was then the difference:

(4) $f^0_{R_W} = 1 - f^0_{M_W}$

After determining the frequencies of the mutant and reference populations, phenotypic selection using flow cytometry was modeled on the total population, X_T, at a predetermined population cutoff, c. The goal was to quantify the frequency of mutant and reference genotypes in each phenotypic bulk. Because X_T is a mixture distribution, the fractions of individuals with mutant and reference alleles present in each bulk were determined from

the reference and mutant phenotypic distributions X_R and X_M. For the high bulk, this required determining the quantiles q_{R_H} and q_{M_H} on X_R and X_M such that q_{R_H} and q_{M_H} equaled the same expression value e_{T_H} and c percent of the total population had higher expression than e_{T_H}.

(5) $$f_{R_W}^0 * q_{R_H} + f_{M_W}^0 * q_{M_H} = 1 - c$$

(6) $$e_{T_H} = \Phi^{-1}(q_{R_H}) = \mu + \sigma * \Phi^{-1}(q_{M_H})$$

Likewise, for the low bulk this required determining quantiles q_{R_L} and q_{M_L} on X_R and X_M such that q_{R_L} and q_{M_L} equaled the same expression value e_{T_L} and c percent of the total population had lower expression than e_{T_L}.

(7) $$f_{R_W}^0 * q_{R_L} + f_{M_W}^0 * q_{M_L} = c$$

(8) $$e_{T_L} = \Phi^{-1}(q_{R_L}) = \mu + \sigma * \Phi^{-1}(q_{M_L})$$

In both instances, $\Phi^{-1}(q)$ is the standard normal quantile function, H and L index the high and low bulks respectively, and e_{T_H} and e_{T_L} are the expression values for the high and low bulks relative to the entire population X_T. We solved the above equations numerically for q_{M_H} and q_{M_L} using *solnp* within Rsolnp (Ghalanos & Theussl 2006) by minimizing the following functions for the high and low bulks respectively:

(9) $$[f_{R_W}^0 * \Phi\left(\mu + \sigma * \Phi^{-1}(q_{M_H})\right) + f_{M_W}^0 * q_{M_H} + c - 1]^2$$

(10) $$[f_{R_W}^0 * \Phi\left(\mu + \sigma * \Phi^{-1}(q_{M_L})\right) + f_{M_W}^0 * q_{M_L} - c]^2$$

where Φ(y) is the cumulative distribution function for the standard normal distribution. From these quantiles, the frequencies of the mutant and reference alleles in the high and low bulks were calculated as the weighted proportion of mutant and reference alleles more extreme than the phenotypic cutoff:

(11) $$f_{M_H}^0 = \frac{f_{M_W}^0 * \Phi\left(\frac{e_{T_H} - \mu}{\sigma}\right)}{f_{R_W}^0 * \Phi(e_{T_H}) + f_{M_W}^0 * \Phi\left(\frac{e_{T_H} - \mu}{\sigma}\right)} = \frac{f_{M_W}^0 * q_{M_H}}{f_{R_W}^0 * q_{R_H} + f_{M_W}^0 * q_{M_H}} = \frac{f_{M_W}^0 * q_{M_H}}{1-c}$$

(12) $$f_{R_H}^0 = 1 - f_{M_H}^0$$

(13) $$f_{M_L}^0 = \frac{f_{M_W}^0 * \Phi\left(\frac{e_{T_L} - \mu}{\sigma}\right)}{f_{R_W}^0 * \Phi(e_{T_L}) + f_{M_W}^0 * \Phi\left(\frac{e_{T_L} - \mu}{\sigma}\right)} = \frac{f_{M_W}^0 * q_{M_L}}{f_{R_W}^0 * q_{R_L} + f_{M_W}^0 * q_{M_L}} = \frac{f_{M_W}^0 * q_{M_L}}{c}$$

(14) $$f_{R_L}^0 = 1 - f_{M_L}^0$$

To model the additional growth necessary to create libraries from the sorted bulks, each bulk was allowed to undergo another *g* generations of growth, assuming that the relative fitness (1-*s*) between genotypes with the mutant and reference alleles of the site affecting fluorescence was the same before and after bulk selection:

(15) $f^1_{M_H} = \frac{f^0_{M_H}(1-s)^g}{f^0_{M_H}(1-s)^g + f^0_{R_H}*1}$

(16) $f^1_{M_L} = \frac{f^0_{M_l}(1-s)^g}{f^0_{M_L}(1-s)^g + f^0_{R_L}*1}$

Simulation of allele frequency estimates from sequencing data: Using the deterministic allele frequencies described above, we simulated the library creation and sequencing processes by drawing the proportion of 'reads' containing the mutant allele from a binomial distribution in each bulk independently:

(17) $T_{M_H} \sim B(V_H, F_{M_H})$

(18) $T_{M_L} \sim B(V_L,\ F_{M_L})$

where V is the distribution of sequencing coverage and F the mutant allele frequency distribution. The sequencing coverage distribution was simulated as a negative binomial distribution (Robinson and Smyth 2007, 2008):

(19) $V \sim NB(\alpha, \beta)$ with mean $\frac{\alpha}{\beta}$ and variance $\frac{\alpha(\beta+1)}{\beta^2}$

To adjust coverage, we varied β (inverse scale) because our data suggested α (shape) was approximately 80 regardless of sequencing depth. Average coverage was set to reflect coverage after mapping and we did not explicitly model sequencing error. To account for sampling during library creation, the mutant allele frequencies were simulated from the deterministic frequencies assuming a binomial distribution:

(21) $F_{M_H} \sim \frac{B\left(n, f^1_{M_H}\right)}{n}$

(22) $F_{M_L} \sim \frac{B\left(n, f^1_{M_L}\right)}{n}$

Reference 'reads' were then assumed to make up the difference between the coverage and the number of mutant 'reads'

(23) $t_{R_H} \sim v_H - t_{M_H}$

(24) $t_{R_L} \sim v_L - t_{M_L}$

A G-test was performed on the counts t_{M_H}, t_{M_L}, t_{R_L} and t_{R_H} to determine significance. Power was calculated as the frequency of simulations where the *P*-value was below 0.001, representing a Bonferonni correction assuming 50 possible mutations.

Comparison between G-test and Fisher's exact test*:* The Fisher's exact test commonly used in the analysis of next generation sequencing data (Kofler *et al.* 2011) assumes that the row and column totals of the two-by-two

contingency table are fixed. This assumption is violated by sequencing data, however, because coverage for each allele results from sampling reads from an underlying distribution. When marginal totals are free to vary, the G-test is more appropriate than the Fisher's exact test. We analyzed our data using both tests and found that their results were very similar (although not identical) except when sequencing coverage was low (Figure S11).

DNA library preparation

Genomic DNA libraries were produced in parallel by modifying a low cost method developed for Illumina sequencing (Rohland and Reich 2012). Briefly, DNA was sheared, Illumina adapters were attached by blunt-end ligation and indexed using PCR. Between enzymatic reactions, DNA was cleaned using custom MagNA beads (Carboxyl-modified Sera-Mag Magnetic Speed-beads in a PEG/NaCl buffer) as a lower-cost substitute for AMPure XP kit. For each sample, 2 µg of genomic DNA (120 µl) was sheared to an average fragment size of 400 bp with a Covaris S220 instrument (Duty cycle: 10%, intensity: 4, cycles/burst: 200, time: 55 s). 1 µg (60 µl) of sheared DNA was purified in 96 µl (1.6x) of MagNA bead solution and resuspended in 20 µl of water. Blunt-end repair was performed using a NEB Quick Blunting Kit by mixing 19 µl of DNA with 2.5 µl of blunting buffer, 2.5 µl of 1 mM dNTP mix and 1 µl of blunt enzyme mix. This mix was incubated for 20 min at 12°C followed by 15 min at 37°C. DNA was then cleaned up in 2x MagNA beads and eluted in 25 µl of water. Next, adapters were ligated using a NEB Quick Ligation Kit. 23.8 µl of blunt DNA was mixed with 30 µl of ligation buffer, 4 µl of P5 + P7 adapter mix (100 µM each) and 1.2 µl of Quick T4 DNA ligase and incubated at 25°C for 20 min. DNA was then cleaned in 1.6x beads, eluted in 40 µl and nick-fill in was done using Bst DNA Polymerase Large Fragment from NEB. 39 µl of DNA sample was mixed with 5 µl of ThermoPol buffer, 4 µl of 25 mM dNTP mix and 2 µl of Bst DNA polymerase (2 U/µl). After 20 min at 37°C, samples were mixed with 1.6x MagNA beads and eluted in 30 µl water. KAPA HiFi PCR Kit was used for indexing PCR: 10 µl of template DNA was mixed with 5 µl of HiFi buffer, 0.75 µl of 10 mM dNTP mix, 0.75 µl primer IS4 (10 µM), 0.75 µl indexing primer (10 µM), 7.25 µl sterile H_2O and 0.5 µl KAPA HiFi polymerase (1 U/µl). PCRs were incubated at 95°C for 4 min followed by 12 cycles at 98°C for 20 s, 64°C for 15 s and 72°C for 20 s with a final extension at 72°C for 5 min. PCR products were then cleaned up in 1.6x MagNA beads and eluted in 40 µl of water. Samples were then processed at the UM Sequencing Core Facility. For each sample, DNA concentration was quantified through qPCR with primers targeting P5 and P7 adapters and using an Agilent 2100 Bioanalyzer. Equimolar amounts of each sample were pooled together for multiplexed sequencing before gel electrophoresis size selection of DNA fragments ranging from 350 bp to 850 bp on a 1% agarose gel. The 8 libraries produced for this project (high- and low-fluorescing bulks for each of the three mutants

plus the original non-mutagenized strain and the mapping strain, all of which were haploid) were combined with 16 libraries constructed for other projects and subjected to 100 bp paired-end sequencing in one lane on Illumina HiSeq2000 platform. Oligonucleotide sequences used for library preparation are listed in Table S1 and barcode sequences used for multiplexing in Table S2. Because average sequencing depth was lower than 75x for two of the samples (YPW89 low bulk and YPW102 low bulk), we decided to re-sequence the corresponding genomic libraries in an independent sequencing lane using the same procedure. All data from the two runs of sequencing were combined for analyses presented in this study.

Tetrad dissection-based approach for mapping

In addition to the high-sensitivity method described above, we mapped the causative mutation altering YFP expression in several mutants including YPW89, YPW94 and YPW102 using a tetrad dissection-based approach (Birkeland *et al.* 2010). First, mutants YPW89 and YPW94 were crossed to Y39 (*MATα leu2Δ0 ura3Δ0* P_{TDH3}*-YFP*) and YPW102 was crossed to Y85 (*MATα met17Δ0 ura3Δ0* P_{TDH3}*-YFP*). Resulting diploids were sporulated in KAc medium, several tetrads were dissected and individual spores were grown on YPD (11 tetrads for YPW89xY39, 8 tetrads for YPW94xY39 and 9 tetrads for YPW102xY85). The fluorescence level of the resulting colonies was quantified through flow cytometry. Each spore was grown in YPD to saturation, then diluted in SC-Arg medium and grown to log-phase at 30°C. Fluorescence (FL1-A) and forward scatter (FSC-A) of thousands of cells were recorded using a HyperCyt Autosampler (IntelliCyt Corp.) coupled to a BD Accuri C6 Flow Cytometer (533/30 nm optical filter used for YFP acquisition). Based on these data, a mutant phenotype was assigned for 2 of the 4 spore progeny from each tetrad. For tetrads derived from YPW89 and YPW94 (increased YFP expression), the two progeny with highest median of FL1-A/FSC-A were considered as mutants. For tetrads derived from YPW102 (decreased YFP expression), the two progeny with lowest median of FL1-A/FSC-A were considered as mutants. These mutant progeny were then cultured separately to saturation in YPD and mixed evenly to a final volume of 2.5 ml. 22 progeny were mixed together for YPW89, 16 for YPW94 and 18 for YPW102. For each pool, genomic DNA was extracted using a Gentra Puregene Yeast/Bacteria Kit from QIAGEN. Next, 2 µg of DNA was sheared with a Covaris S220 instrument and genomic libraries were prepared using NEBNext E6040 kit. An in-line barcoding strategy was adopted for multiplexing. Briefly, 3' A overhang was added to end-repaired DNA fragments. Then, barcoded adapters were ligated to dA-tailed DNA, creating Y-shaped products whose extremities are single-stranded. PCR using standard Illumina primers allowed the addition of adapter sequences attaching to Illumina flow cells. PCR products ranging from 400bp to 800bp were size

selected on an agarose gel. Barcodes, adapters and PCR primer sequences are listed in Table S3 and Table S4. 22 libraries were pooled together and 100 bp paired-end reads were sequenced on a single lane of HiSeq2000 flow cell at the University of Michigan Sequencing Core. Sequencing data were analyzed through the same pipeline as described above, except that only mutant segregant pools were sequenced in this case. G-tests were performed by comparing observed mutation frequency in the mutant pool to a null expectation of 0.5.

Quantification of allele frequencies through pyrosequencing

To assess the accuracy of allele frequency estimates obtained through Illumina sequencing, quantitative genotyping of the low and high fluorescence bulks was performed for three variable sites in each mutant using pyrosequencing. These included the site with strongest allele frequency difference between bulks as well as two sites showing no significant difference in allele frequency. Pyrosequencing assays (see File S3) were designed following manufacturer instructions (PyroMark Assay Design software from QIAGEN), except that a universal biotinylated primer was used to reduce the cost. For each variant assessed, PCR reactions were performed as previously described (Aydin *et al.* 2006) on 5 different genomic DNA templates from the original haploid mutant, the haploid mapping strain, the F1 diploid hybrid and the low and high fluorescence haploid segregants. Quantitative genotyping was performed on a PyroMark ID instrument following the protocol described in Wittkopp (2011). Data from parental strains and the hybrid were used to correct for potential PCR or sequencing biases. Knowing that true allele frequencies are 1, 0 and 0.5 in the mutant, mapping strain, and hybrid, a 2nd degree polynomial regression model was fitted to the observed data and used to correct allele frequencies in the segregant bulks.

Literature cited

Aydin, A., M. R. Toliat, S. Bähring, C. Becker, and P. Nürnberg, 2006 New universal primers facilitate Pyrosequencing. Electrophoresis 27: 394–397.

Birkeland, S. R., N. Jin, A. C. Ozdemir, R. H. Lyons, L. S. Weisman *et al.*, 2010 Discovery of mutations in *Saccharomyces cerevisiae* by pooled linkage analysis and whole-genome sequencing. Genetics 186: 1127–1137.

Ghalanos, A., & Theussl, S. (2012). Rsolnp: General Non-linear Optimization Using Augmented Lagrange Multiplier Method. R package version 1.14.

Kofler, R., R. V. Pandey, and C. Schlötterer, 2011 PoPoolation2: identifying differentiation between populations using sequencing of pooled DNA samples (Pool-Seq). Bioinformatics (Oxford, England) 27: 3435–3436.

Robinson, M. D., and G. K. Smyth, 2007 Moderated statistical tests for assessing differences in tag abundance. Bioinformatics (Oxford, England) 23: 2881–2887.

Robinson, M. D., and G. K. Smyth, 2008 Small-sample estimation of negative binomial dispersion, with applications to SAGE data. Biostatistics (Oxford, England) 9: 321–332.

Rohland, N., and D. Reich, 2012 Cost-effective, high-throughput DNA sequencing libraries for multiplexed target capture. Genome research 22: 939–946.

Wittkopp, P. J., 2011 Using pyrosequencing to measure allele-specific mRNA abundance and infer the effects of *cis*- and *trans*-regulatory differences. Molecular Methods for Evolutionary Genetics 772: 297–317.

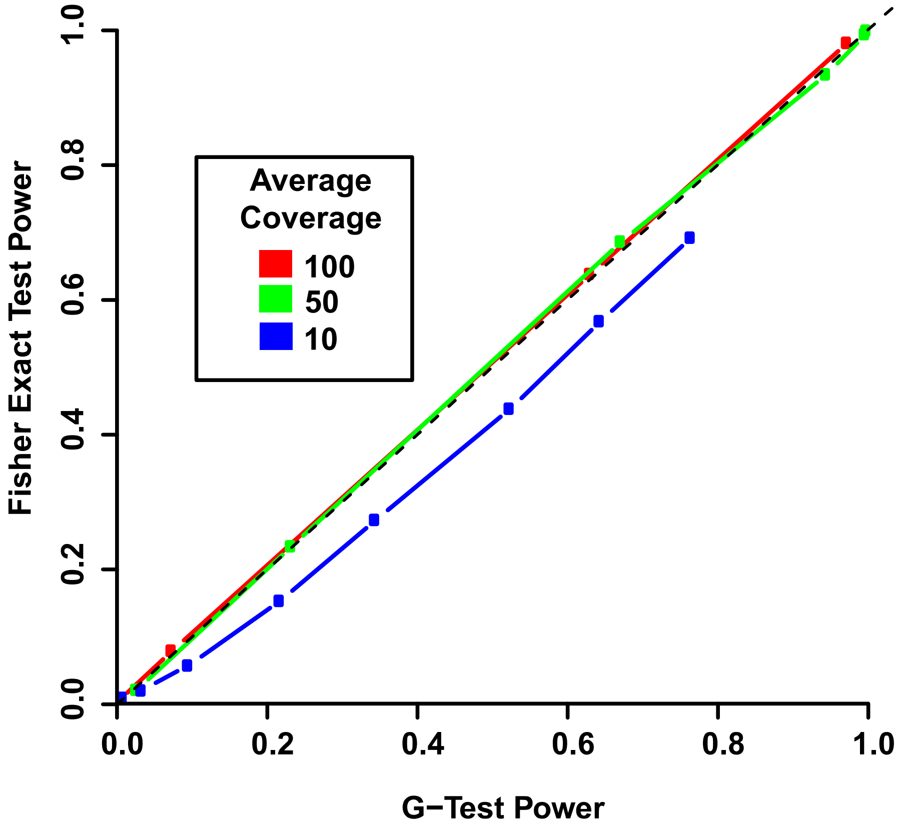

Figure S11 Comparison of statistical power using Fisher's exact test and G-test. Power to detect a significant difference in allele frequency between bulks for different mutation effect sizes and sequencing depths is shown. Dots on each line represent different mutation effects ranging from 0% to +25% (bottom left to top right) relative to WT mean expression. Fixed parameter values were: Standard Deviation = 100%, Selection Coefficient = 0.03, Population Size = 10^7, Cutoff Percent = 5%, Generations = 20.

Table S1 Sequences of oligonucleotide adapters used for library preparation in the FACS-based mapping approach.

Oligo ID	Oligo Sequence 5'-3' (* indicates Phosphorothioate bound)
IS1_adapter.P5	A*C*A*CTCTTTCCCTACACGACGCTCTTCCGA*T*C*T
IS2_adapter.P7	G*T*G*ACTGGAGTTCAGACGTGTGCTCTTCCGA*T*C*T
IS3_adapter.P5+P7	A*G*A*TCGGAAG*A*G*C
IS4_indPCR.P5	AATGATACGGCGACCACCGAGATCTACACTCTTTCCCTACACGACGCTCTT

Table S2 Indexing oligos and barcodes used for library preparation in the FACS-based mapping approach.

Oligo ID	Oligo Sequence 5'-3' (Lowercase: Index barcode)	Barcode	Sample
indexing4	CAAGCAGAAGACGGCATACGAGATttgatccGTGACTGGAGTTCAGACGTGT	GGATCAA	YPW89.low
indexing5	CAAGCAGAAGACGGCATACGAGATatcttgcGTGACTGGAGTTCAGACGTGT	GCAAGAT	YPW94.low
indexing6	CAAGCAGAAGACGGCATACGAGATtctccatGTGACTGGAGTTCAGACGTGT	ATGGAGA	YPW102.low
indexing12	CAAGCAGAAGACGGCATACGAGATacttcaaGTGACTGGAGTTCAGACGTGT	TTGAAGT	YPW89.high
indexing13	CAAGCAGAAGACGGCATACGAGATtgatagtGTGACTGGAGTTCAGACGTGT	ACTATCA	YPW94.high
indexing14	CAAGCAGAAGACGGCATACGAGATgatccaaGTGACTGGAGTTCAGACGTGT	TTGGATC	YPW102.high
indexing19	CAAGCAGAAGACGGCATACGAGATgagattcGTGACTGGAGTTCAGACGTGT	GAATCTC	WT
indexing20	CAAGCAGAAGACGGCATACGAGATgagcatgGTGACTGGAGTTCAGACGTGT	CATGCTC	Mapping.Strain

Only eight samples used in this study are shown. These eight samples were multiplexed with 16 other samples using the following barcodes: 1-TCGCAGG, 2-CTCTGCA, 3-CCTAGGT, 4-GGATCAA, 5-GCAAGAT, 6-ATGGAGA, 7-CTCGATG, 8-GCTCGAA, 9-ACCAACT, 10-CCGGTAC, 11-AACTCCG, 12-TTGAAGT, 13-ACTATCA, 14-TTGGATC, 15-CGACCTG, 16-TAATGCG, 17-AGGTACC, 18-TGCGTCC, 19-GAATCTC, 20-CATGCTC, 21-ACGCAAC, 22-GCATTGG, 23-GATCTCG, 24-CAATATG.

Table S3 Sequences of oligonucleotide adapters used for library preparation in the tetrad-based mapping approach.

Oligo ID	Oligo Sequence 5'-3'
Indexed adapter 1	ACACTCTTTCCCTACACGACGCTCTTCCGATCTNNNNNNT
Indexed adapter 2	NNNNNNAGATCGGAAGAGCGGTTCAGCAGGAATGCCGAG
PCR primer 1	AATGATACGGCGACCACCGAGATCTACACTCTTTCCCTACACGACGCTCTTCCGATCT
PCR primer 2	CAAGCAGAAGACGGCATACGAGCTCTTCCGATCT

Underlined: barcode. Color: Same color shows complementary regions where annealing occurs during PCR.

Table S4 Barcodes used for library preparation in the tetrad dissection-based mapping approach.

Barcode	Sample
ACCAGG	Y1
AAGGCC	Y39
TATTCG	Y54 x Y85
CGGAAC	Y85
ATACCT	Y89 x Y39
ACACGA	Y94 x Y39
CACATA	Y102 x Y85

Only seven samples used in this study are presented. These seven samples were multiplexed with 14 other samples using the following barcodes: 1-ACCAGG, 2-AAGGCC, 3-TCTGAT, 4-CAAGTG, 5-TACGTT, 6-TATTCG, 7-CGGAAC, 8-ATACCT, 9-GTGCTG, 10-GGCGTA, 11-TGCACG, 12-CTACGC, 13-ACACGA, 14-CCGTAG, 15-GTAACA, 16-GTGTAT, 17-AGGTTC, 18-CACATA, 19-AGTTGG, 20-GCTCAA, 21-TTGACT, 22-TCTCGG.

File S2

R script for power analyses

File S2 is available for download as an R source file at

http://www.g3journal.org/lookup/suppl/doi:10.1534/g3.114.011783/-/DC1

File S3

Description of pyrosequencing assays for quantitative genotyping of seven genetic variants in the segregant bulks

File S3 is available for download as an Excel file at

http://www.g3journal.org/lookup/suppl/doi:10.1534/g3.114.011783/-/DC1

File S4

Robustness of the BSA-seq approach

Impact of genomic position on mapping success: To determine the limits of our bulk segregant mapping protocol, we tested whether the three causal mutations we identified would have been successfully mapped if they had been located somewhere else in the genome. This might not be the case if the power to map a mutation of a given effect size was uneven across the genome, either because of random fluctuation in sequencing depth or because of reads failing to align uniquely to the genome. To examine this possibility, we first computed for each bulk sample the sequencing depth at every genomic position using *genomecov* tool in BEDTools v2.17.0 (Quinlan and Hall 2010). We then inferred, for each genomic position in each segregant bulk, the number of mutant and wild type alleles we would have observed if the site was causative given the coverage of the position and the mutation frequency at the actual causative site. We then calculated the fraction of genomic positions for which a mutation with the same effect as the actual causative mutation would have been detected and called significant using the analysis pipeline described in Figure 4.

Depending on the mutant considered, we found that 2.9% to 3.4% of genomic positions were not covered by any sequencing reads in at least one sample (Figure S10A, left bars), making it impossible to test for a significant association. Additionally, 4.1% to 5.1% of genomic positions failed to meet the minimum cutoff of 10 reads in the merged bulks that we required for the site to be called as a high confidence SNP (Figure S10A, middle bars). These sites were thus not tested for a significant association with the fluorescence phenotype and the causative mutation would have remained undetected if located at one of these positions. Finally, we found that 4.2% to 4.9% of sites had insufficient sequencing coverage to yield a significant phenotypic association in a G-test (Figure S10A, right bars), most of which also failed to meet the 10 read minimum criterion to be called a SNP. Low sequence read coverage at these sites could be caused by random fluctuations in sequencing depth or problems aligning sequence reads that contain these sites.

To determine how often sites with low coverage resulted from poor alignment of sequence reads, we assessed mappability for each position in the reference genome using software from the GEM library (Derrien *et al.* 2012). A genomic site was considered to have perfect mappability if and only if every possible read overlapping that site aligned uniquely to the correct genomic position (Stevenson *et al.* 2013). Aligning 100 bp sequences to the reference genome while allowing up to five mismatches showed imperfect mappability for 6.8% of the *S. cerevisiae* genome (Figure S10B). More than 97% of these sites were included in at least one of the three groups of problematic

sites described above (Figure S10B), indicating that the inability to uniquely map sequence reads, rather than random variation in sequencing depth, was responsible for the vast majority of sites with low coverage in our dataset. This interpretation is further supported by the genome-wide distributions of sequencing coverage showing two peaks -- one centered at the mean coverage for each sample and the other at 0 (see Figure S9).

If a causative mutation occurs in a low mappability region, it would remain undetected, but linked mutations could still yield a significant association of the phenotype to a broader genomic region. However, such mapping by linkage is likely to occur only if the average distance between mutations is smaller than the extent of genetic linkage. Linkage extends approximately 50 kb after a single generation of meiosis in *S. cerevisae* (Mortimer *et al.* 1991). Given the number of mutations in each mutant isolated in Gruber *et al.* (2012), an average of one mutation is expected every 255 kb, making linkage unlikely for most pairs of sites. Assuming all of these mutations are indeed unlinked, we conclude that a small portion of the genome (~4% on Figure S10A, middle bars) is unsuitable to mapping in these mutants using short-read data regardless of sequencing depth.

Impact of decreased sequencing depth on mapping success: To determine how variant calling might have affected our results, we assessed the total number of mutations called for each mutant using the bulk sequencing data when reads from the SAM files were randomly subsampled to a genome coverage ranging from 10x to 110x using the Picard (v1.97) command-line tool *DownsampleSam* (http://picard.sourceforge.net). For all three mutants, a steep drop was observed in the total number of mutations called at 10x coverage relative to 25x coverage (Figure S10C). As expected, sites with the lowest read counts for mutant alleles were the first to be missed when sequencing depth was decreased. Interestingly, the only mutation missed in YPW89 mutant when sequencing coverage was reduced to 75x was the causative mutation. This was because this mutation also strongly reduced fitness (Table 3), causing the number of mutant alleles in both bulks to be very low. With decreased coverage, the number of sequencing reads overlapping this site quickly fell below the minimum required for detection as a high confidence SNP.

Finally, we determined how the significance of G-tests used to identify associated sites varied with sequencing depth. The read number for reference and mutant alleles at the causative site were divided by the same values, so that the average sequencing depth between low and high bulks at the site was 80, 70, 60, 50, 40, 30, 20 and 10. We found that the statistical significance of associations between causal sites and YFP fluorescence decreased linearly with sequencing depth, but at different rates for different mutants (Figure S10D). For YPW102, as few as 10 reads overlapping the causal site were required to detect a significant association, whereas 15 and 41 reads were

required in YPW94 and YPW89, respectively. YPW89 was again found to be the most sensitive to a decrease in sequencing depth despite having the strongest effects on mean fluorescence because its effects on fitness decreased its frequency in both bulks (Figure S10C-D).

Literature cited

Derrien, T., J. Estellé, S. Marco Sola, D. G. Knowles, E. Raineri *et al.*, 2012 Fast Computation and Applications of Genome Mappability. PLoS ONE 7: e30377.

Gruber, J. D., K. Vogel, G. Kalay, and P. J. Wittkopp, 2012 Contrasting Properties of Gene-Specific Regulatory, Coding, and Copy Number Mutations in *Saccharomyces cerevisiae*: Frequency, Effects, and Dominance. PLoS genetics 8: e1002497.

Mortimer, R. K., D. Schild, C. R. Contopoulou, and J. A. Kans, 1991 [57] Genetic and physical maps of *Saccharomyces cerevisiae*. Methods in Enzymology 194: 827–863.

Quinlan, A. R., and I. M. Hall, 2010 BEDTools: a flexible suite of utilities for comparing genomic features. Bioinformatics (Oxford, England) 26: 841–842.

Stevenson, K. R., J. D. Coolon, and P. J. Wittkopp, 2013 Sources of bias in measures of allele-specific expression derived from RNA-seq data aligned to a single reference genome. BMC genomics 14: 536.
