# Supplementary material for: Mapping Small Effect Mutations in Saccharomyces cerevisiae: Impacts of Experimental Design and Mutational Properties
Source: G3 (Bethesda). 2014 Apr 29;4(7):1205–16. doi: 10.1534/g3.114.011783 (PMC4455770; doi:10.1534/g3.114.011783)
Supplement: Supporting Information [file supp_g3.114.011783_FigureS1.pdf]

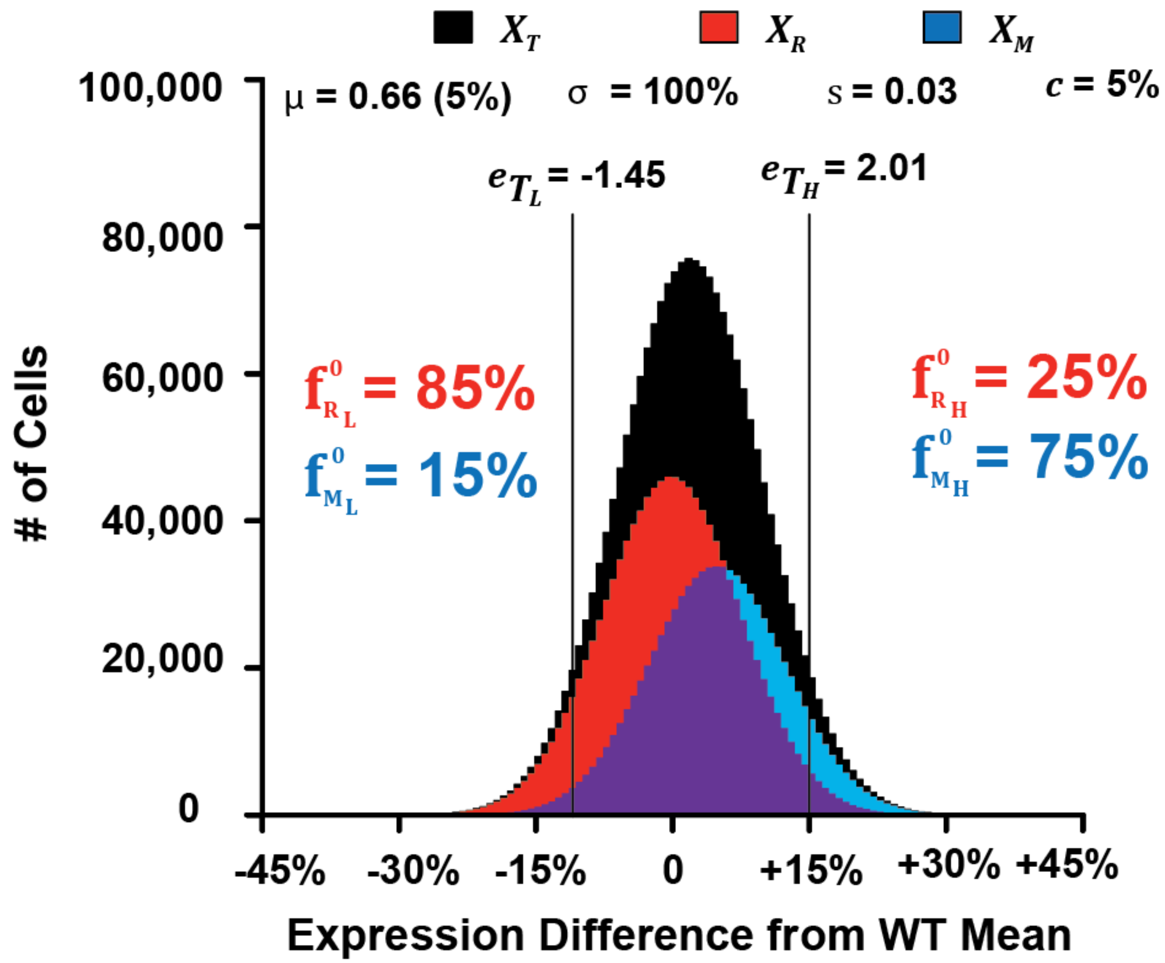

Figure S1 Example of phenotypic distribution after the deterministic phase of our simulation establishing the segregant pools. For a full description of the model used to generate these distributions, see File S1. The phenotypic distribution for all cells in the population (X_T) is shown in black, whereas the phenotypic distributions for cells carrying the reference (X_R) and mutant (X_M) alleles of the causative site are shown in red and blue, respectively. Black lines show the 5th and 95th percentiles of the phenotypic distribution for all cells, which correspond to the thresholds used for sorting with a 5% cutoff for the high and low bulks. The frequency of the reference allele (f_R) and the frequency of the mutant allele (f_M) are shown for both the low (L) and high (H) bulks. Results are shown for a causative mutation that changes the mean (μ) by 5%, has no effect on the phenotypic standard deviation (σ), and has a selection coefficient (s) of 0.03, when the selected bulks are obtained using a 5% cutoff (c).
