# Supplementary material for: Mapping Small Effect Mutations in Saccharomyces cerevisiae: Impacts of Experimental Design and Mutational Properties
Source: G3 (Bethesda). 2014 Apr 29;4(7):1205–16. doi: 10.1534/g3.114.011783 (PMC4455770; doi:10.1534/g3.114.011783)
Supplement: Supporting Information [file supp_g3.114.011783_FigureS2.pdf]

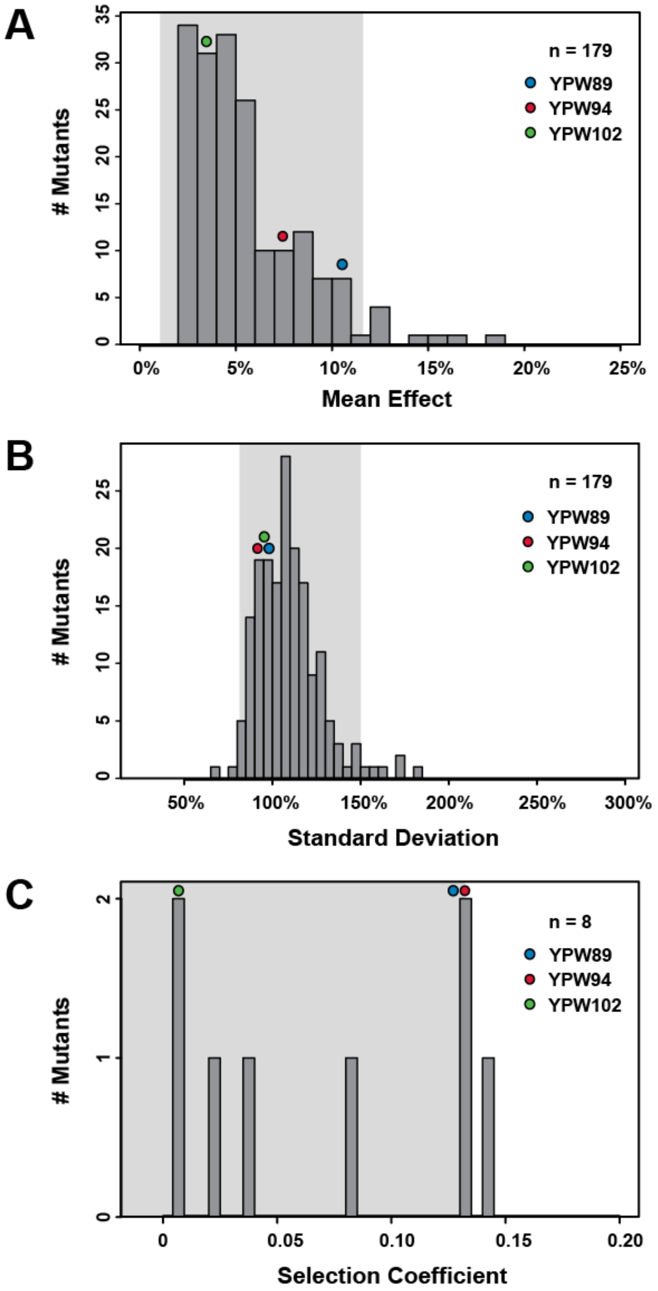

Figure S2 Phenotypic effects of the *trans*-regulatory mutants described in Gruber *et al.* (2012). Absolute values of effects on mean expression level (A) and standard deviation of fluorescence (B) relative to wild type are shown for the full set of 179 *trans*-regulatory mutants. (C) Selection coefficients for 8 randomly selected mutants, including the three mutants used for mapping in this study (YPW89, YPW94 and YPW102), are shown. Shaded regions show confidence intervals excluding the 10% most extreme mutants and correspond to the shaded regions in Figure 1 and Figure 2. Colored dots indicate the parameter values for the three mutants analyzed in this study.
