# Supplementary material for: Mapping Small Effect Mutations in Saccharomyces cerevisiae: Impacts of Experimental Design and Mutational Properties
Source: G3 (Bethesda). 2014 Apr 29;4(7):1205–16. doi: 10.1534/g3.114.011783 (PMC4455770; doi:10.1534/g3.114.011783)
Supplement: Supporting Information [file supp_g3.114.011783_FigureS3.pdf]

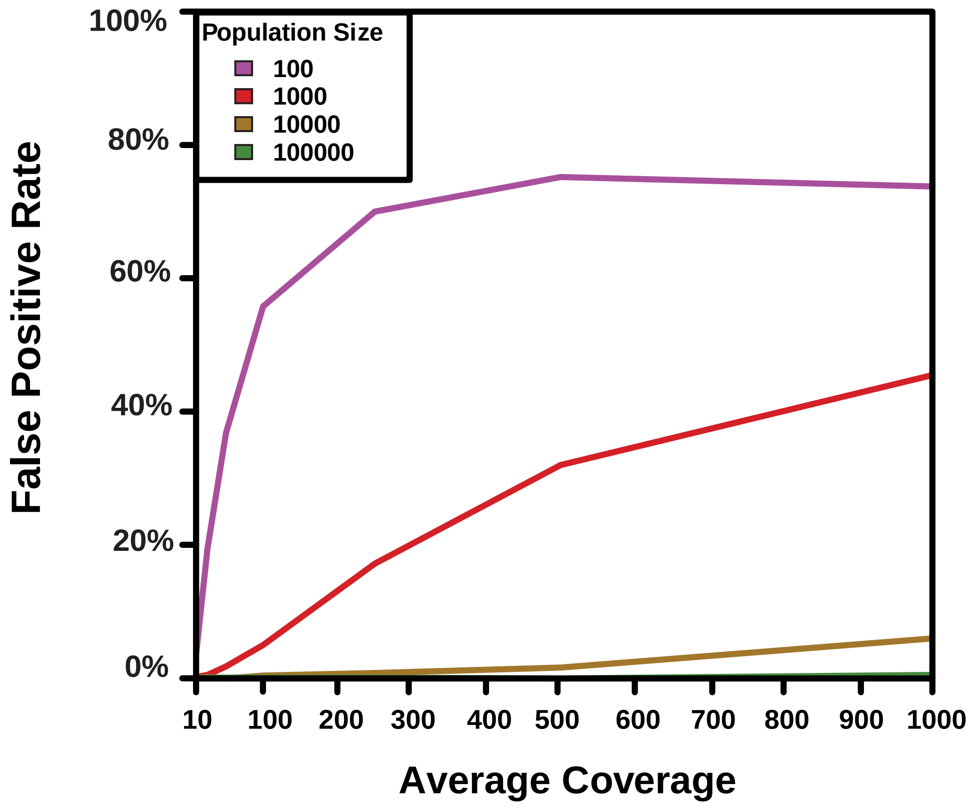

Figure S3 Statistical power to detect a difference in the frequency of a neutral mutation (mean effect = 0%) between bulks depending on average depth of coverage and population size. This power corresponds to the false discovery rate.
