# Supplementary material for: Mapping Small Effect Mutations in Saccharomyces cerevisiae: Impacts of Experimental Design and Mutational Properties
Source: G3 (Bethesda). 2014 Apr 29;4(7):1205–16. doi: 10.1534/g3.114.011783 (PMC4455770; doi:10.1534/g3.114.011783)
Supplement: Supporting Information [file supp_g3.114.011783_FigureS4.pdf]

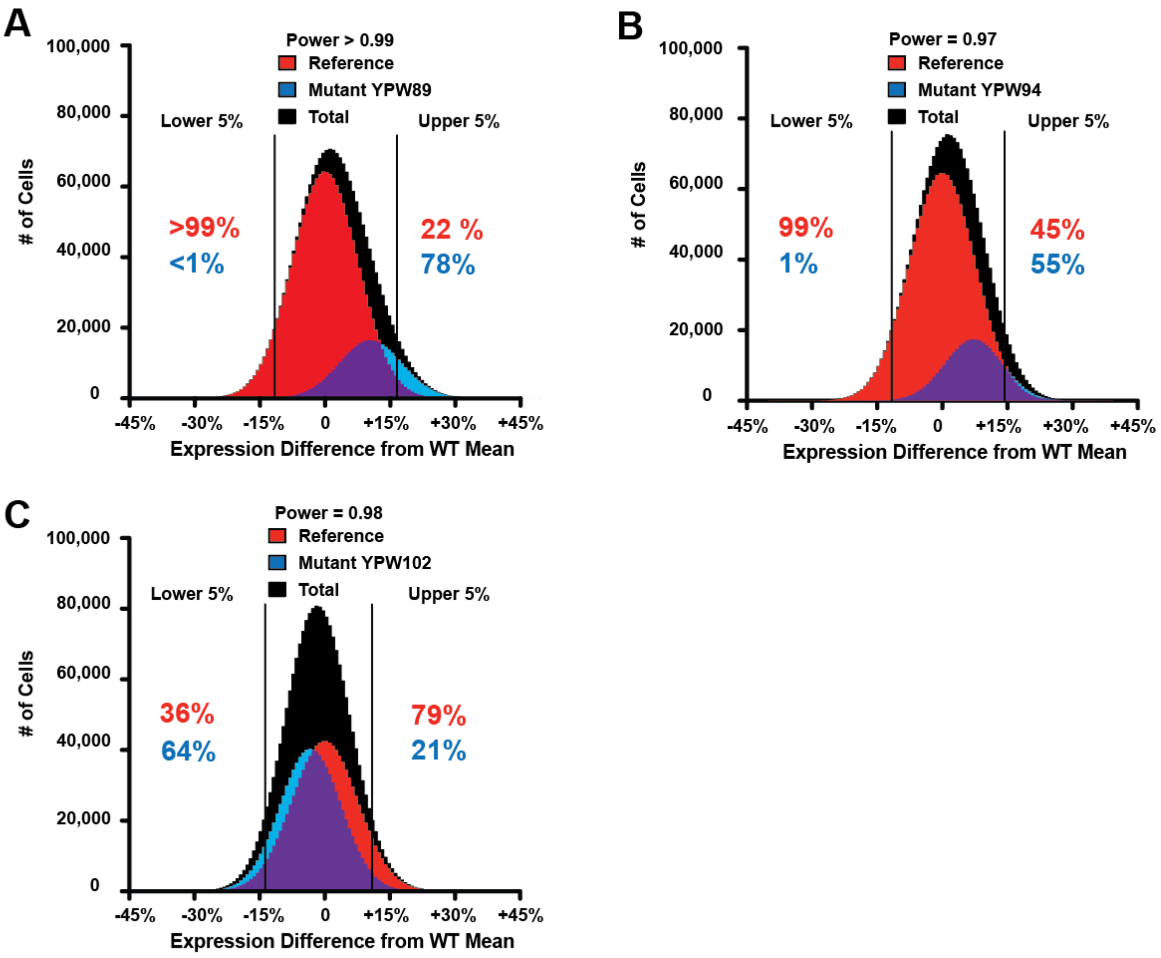

Figure S4 Phenotypic distributions after the deterministic phase of the simulations. Results are shown for mutant YPW89 (A), YPW94 (B), and YPW102 (C). Parameters used for the mean, standard deviation, and selection coefficient of the mutant causal allele were estimated from fluorescence and fitness phenotypes of the mutant strains (Table 1). Black: Total population distribution; Red: Reference allele containing population distribution; Blue: Mutant allele containing population distribution. Black lines show the 5% and 95% cutoffs on the total (black) distribution. Numbers in red indicate the frequency of the reference allele in the two bulks while numbers in blue indicate the frequency of the mutant allele in the two bulks. The power to detect a significant difference ($P < 0.001$) in mutation frequency between lower and higher tails in a G-test given an average sequencing coverage of 75 is shown above each plot.
