# Supplementary material for: Mapping Small Effect Mutations in Saccharomyces cerevisiae: Impacts of Experimental Design and Mutational Properties
Source: G3 (Bethesda). 2014 Apr 29;4(7):1205–16. doi: 10.1534/g3.114.011783 (PMC4455770; doi:10.1534/g3.114.011783)
Supplement: Supporting Information [file supp_g3.114.011783_FigureS5.pdf]

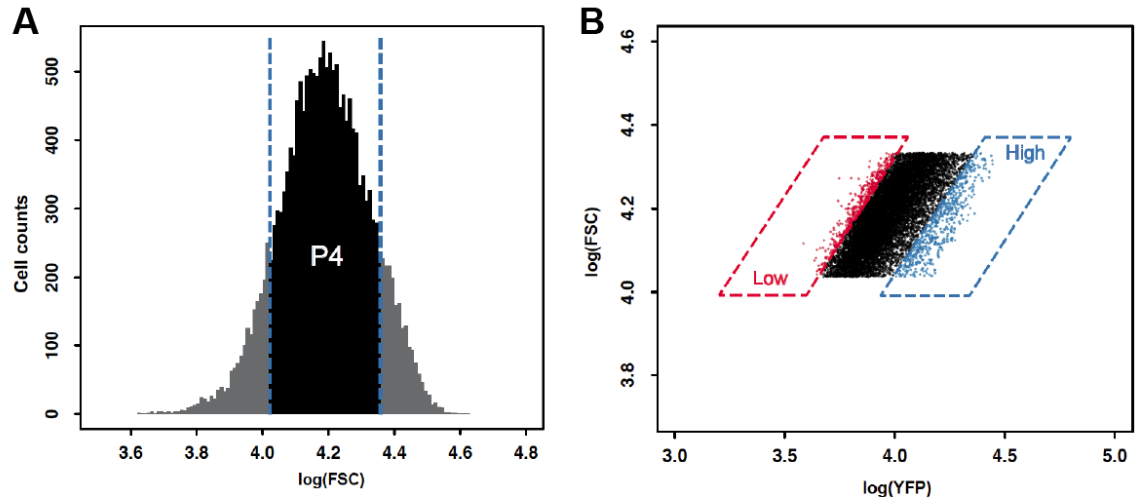

C

Population	#Events	%Parent	%Total	FSC Median	YFP Median
All Events	50,000	----	100.0	25,293	13,873
P4	12,155	86.5	24.3	14,916	9,006
Low Bulk	391	3.3	0.8	14,266	6,000
High Bulk	385	3.2	0.8	14,249	17,909

Figure S5 FACS gating used to collect low and high fluorescence pools. Data shown is from the analysis of mutant YPW102. Gating based on the relationship between FSC-A and FSC-H was used to remove cell doublets (not shown). (A) Gating based on FSC-A, which is a proxy for cell size, was then used to exclude the smallest ~8% and the largest ~8% of events. (B) Finally, low and high bulks were selected based on fluorescence level (log(YFP)) and cell size (log(FSC-A)). Careful attention was paid to select bulks with different fluorescence levels, but similar cell sizes. (C) Changes in event number (cells) resulting from the gates shown in panels (A) and (B).
