# Supplementary material for: Mapping Small Effect Mutations in Saccharomyces cerevisiae: Impacts of Experimental Design and Mutational Properties
Source: G3 (Bethesda). 2014 Apr 29;4(7):1205–16. doi: 10.1534/g3.114.011783 (PMC4455770; doi:10.1534/g3.114.011783)
Supplement: Supporting Information [file supp_g3.114.011783_FigureS6.pdf]

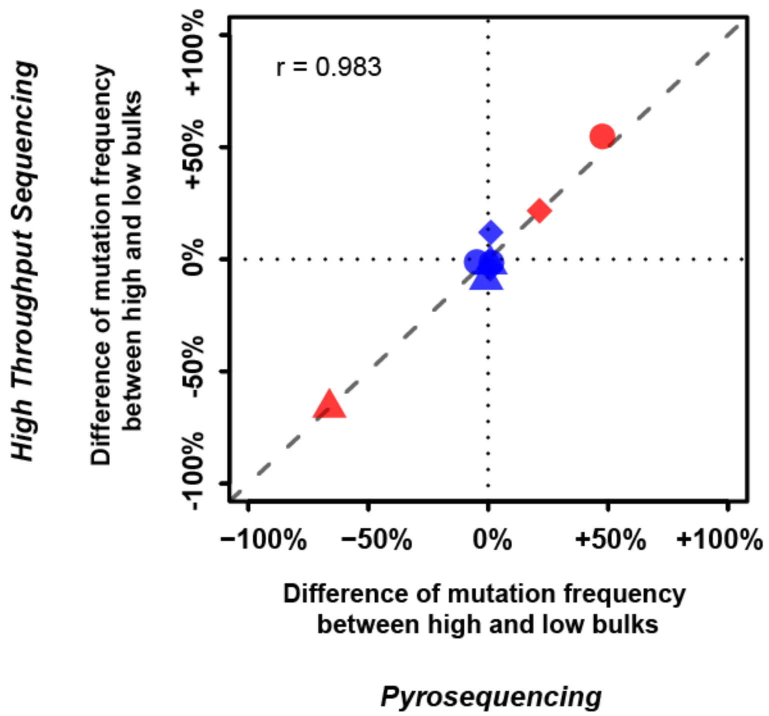

Figure S6 Measures of allele frequency derived from high-throughput sequencing were highly correlated with measures derived from pyrosequencing. For each mutant, pyrosequencing assays were developed for quantitative genotyping of two phenotypically neutral sites (blue) as well as for the site with the highest significance of association with the fluorescence phenotype (red). The plot shows the difference in mutant allele frequency between the high fluorescence and low fluorescence bulks for each site as determined by pyrosequencing (x-axis) or whole genome sequencing (y-axis). Different shapes represent sites analyzed in different mutants (diamond: YPW89, circle: YPW94, triangle: YPW102).
