# Supplementary material for: Mapping Small Effect Mutations in Saccharomyces cerevisiae: Impacts of Experimental Design and Mutational Properties
Source: G3 (Bethesda). 2014 Apr 29;4(7):1205–16. doi: 10.1534/g3.114.011783 (PMC4455770; doi:10.1534/g3.114.011783)
Supplement: Supporting Information [file supp_g3.114.011783_FigureS7.pdf]

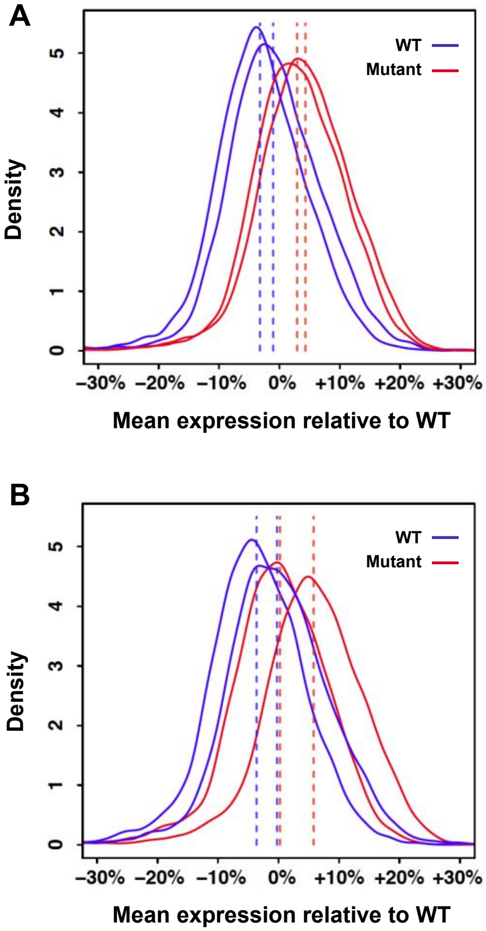

Figure S7 Spore phenotypes assayed after tetrad dissection show phenotypic differences between spores with and without the causative site in some, but not all, cases. Segregation of the YFP phenotype in two tetrads derived from mutant YPW54 are shown. (A) Tetrad showing a clear 2:2 segregation of fluorescence level. (B) Tetrad for which mutant and wild-type progeny are hard to distinguish based on fluorescence, potentially leading to incorrect assignment to a phenotypic pool when assembling mutant and reference pools for mapping. Blue and red solid lines show distributions of fluorescence for populations derived from spores assumed to harbor wild type and mutant alleles of the causative site, respectively. Dotted lines indicate the median fluorescence level for each of the wild-type (blue) and mutant (red) populations.
