# Supplementary material for: Mapping Small Effect Mutations in Saccharomyces cerevisiae: Impacts of Experimental Design and Mutational Properties
Source: G3 (Bethesda). 2014 Apr 29;4(7):1205–16. doi: 10.1534/g3.114.011783 (PMC4455770; doi:10.1534/g3.114.011783)
Supplement: Supporting Information [file supp_g3.114.011783_FigureS8.pdf]

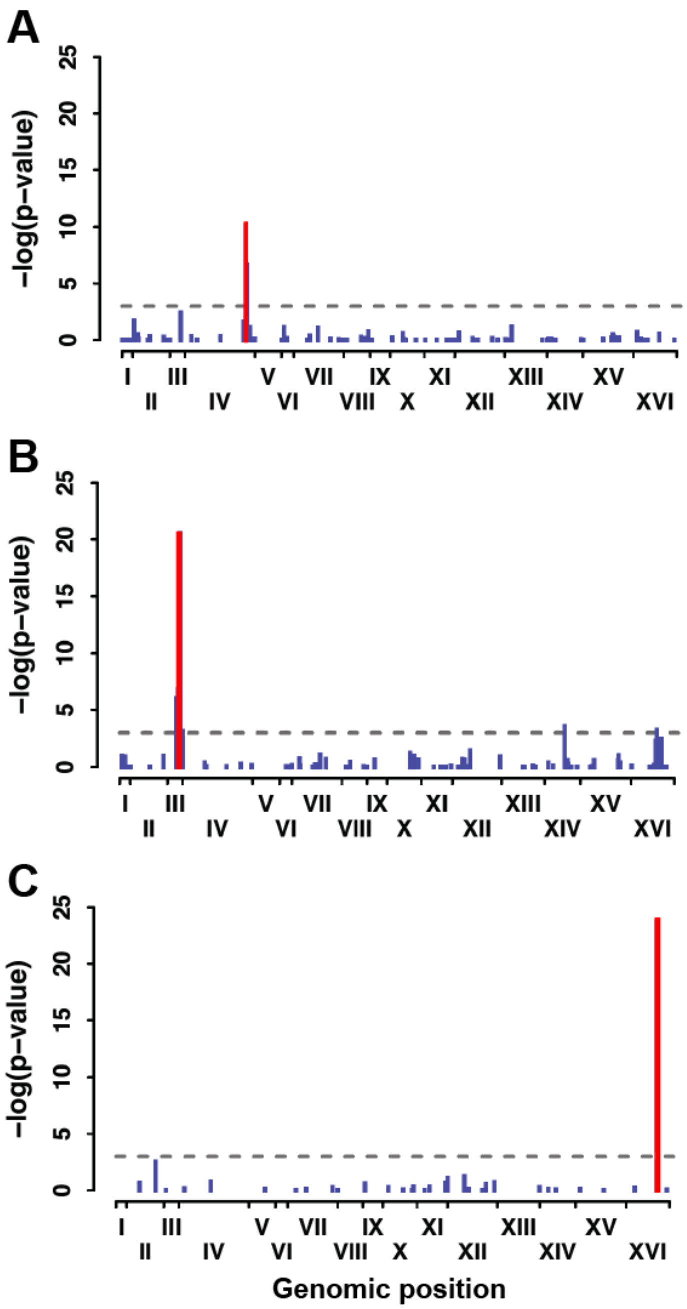

Figure S8 Tetrad-based mapping identifies the same candidate sites as BSA-seq. Mapping results from a more traditional mapping approach based on tetrad dissection are shown for mutant YPW89 (A), YPW94 (B) and YPW102 (C). Colored bars represent individual EMS-induced mutations with their genomic position represented on x-axis and significance of the difference in allele frequency between low fluorescence and high fluorescence bulks represented on y-axis (negative logarithm of *P*-value from G-test). For each mutant, the most significant site identified by BSA-seq is shown in red. Horizontal dotted lines represent a significance threshold of α=0.001.
