# Supplementary material for: Mapping Small Effect Mutations in Saccharomyces cerevisiae: Impacts of Experimental Design and Mutational Properties
Source: G3 (Bethesda). 2014 Apr 29;4(7):1205–16. doi: 10.1534/g3.114.011783 (PMC4455770; doi:10.1534/g3.114.011783)
Supplement: Supporting Information [file supp_g3.114.011783_FigureS9.pdf]

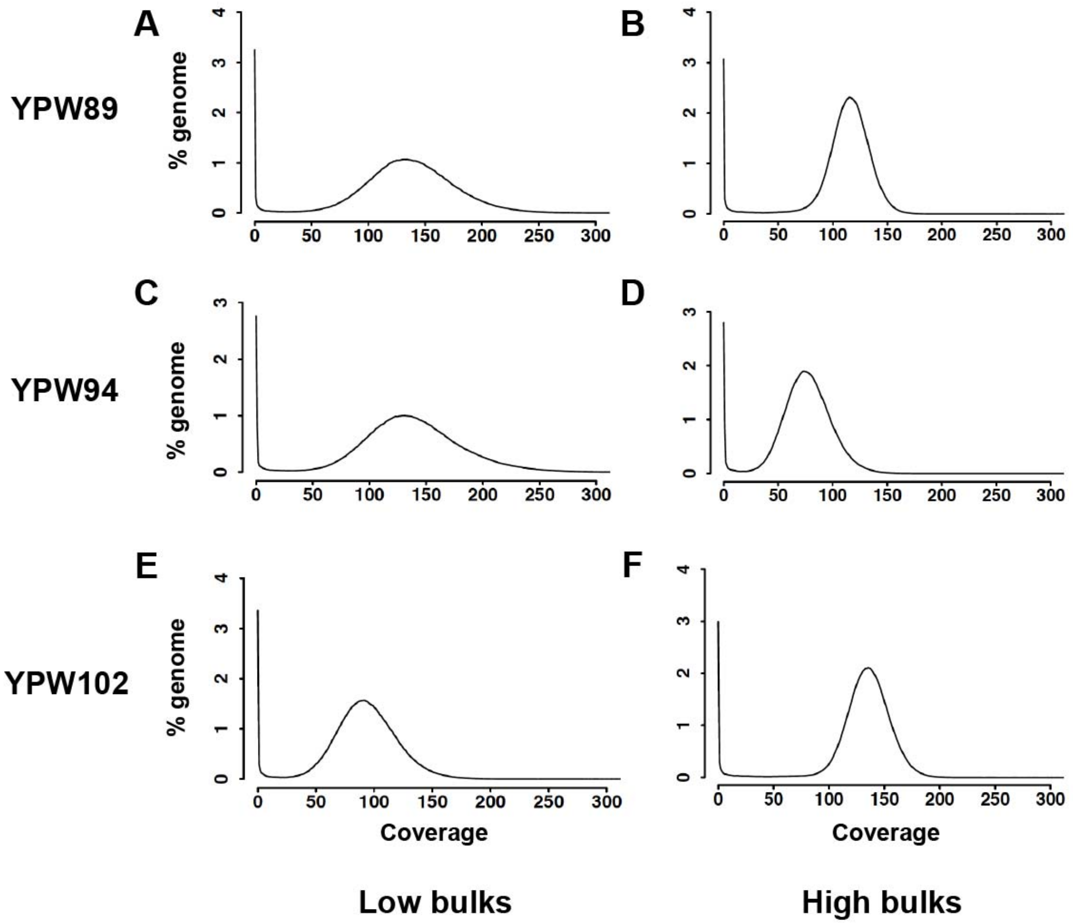

Figure S9 Sequencing coverage for each bulk shows two peaks. Distributions of sequencing coverage across reference genome are shown for low (A,C,E) and high (B,D,F) bulks obtained from mutants YPW89 (A,B), YPW94 (C,D) and YPW102 (E,F). Note the peak at 0, which indicates sites with no overlapping sequencing reads, in addition to the peak near the average coverage for each sample.
