# Supplementary material for: Mapping Small Effect Mutations in Saccharomyces cerevisiae: Impacts of Experimental Design and Mutational Properties
Source: G3 (Bethesda). 2014 Apr 29;4(7):1205–16. doi: 10.1534/g3.114.011783 (PMC4455770; doi:10.1534/g3.114.011783)
Supplement: Supporting Information [file supp_g3.114.011783_FigureS10.pdf]

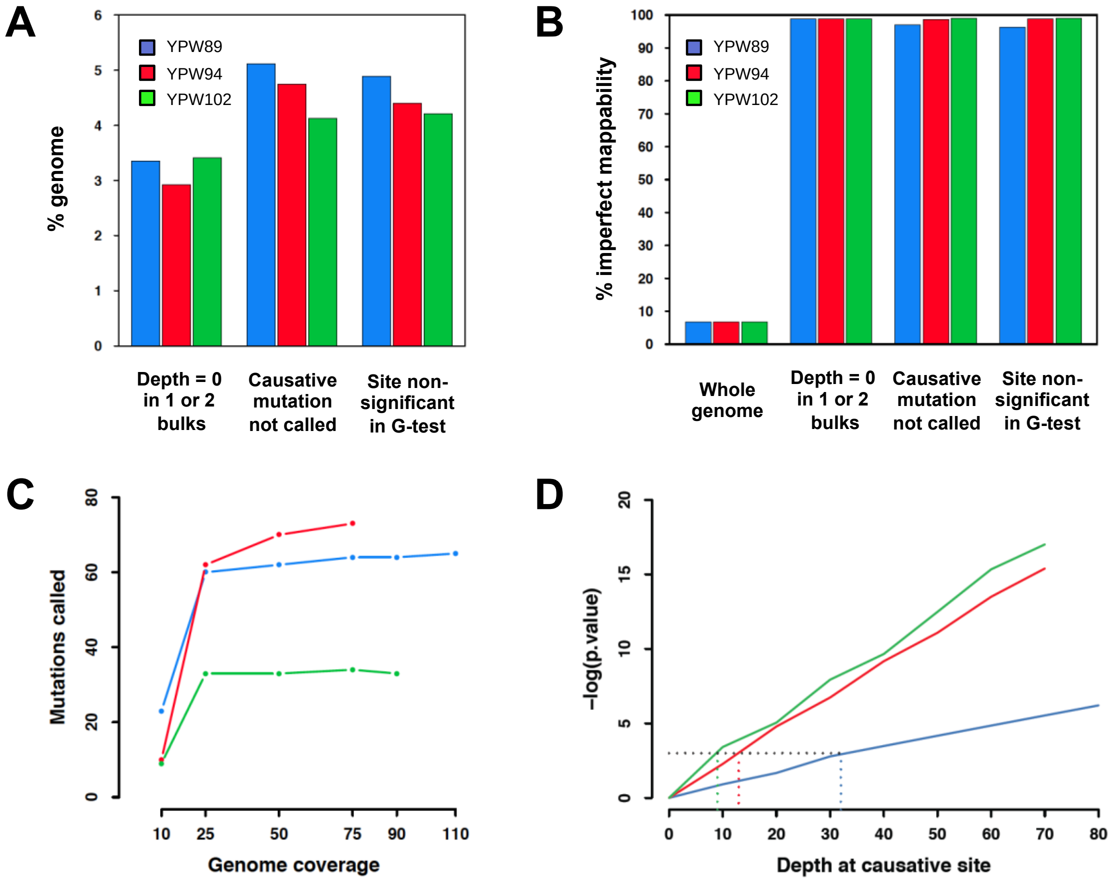

Figure S10 Poor mapping rather than fluctuations in sequencing coverage due to random sampling are responsible for most mapping blind spots. (A) For each mutant, the fraction of the genome with insufficient coverage to detect a putative causative mutation because zero reads were observed in one or both bulks (left), the causative mutation was not called as a sequence variant (middle), or the number of reads mapped was insufficient to generate a significant G-test (right) is shown. (B) Proportion of sites with imperfect mappability across the whole genome and for each genomic class considered in (A) are shown. The vast majority of sites for which a putative mutation could not be detected also showed poor mappability. (C) Robustness of the total number of mutations called to variation in sequencing depth is shown. For each mutant, SNPs were called after subsampling mapped reads to a sequencing depth of 90x, 75x, 50x, 25x and 10x in low and high bulks. Mutants are color coded as in panel (A). (D) Significance of the causative site depending on its coverage is shown. For a constant mutation frequency at the causative site, the total number of alleles was decreased from 90 to 0 (x-axis) and the *P*-value of the G-test was computed (y-axis). Mutants are color coded as in panel (A). Dotted lines highlight the threshold of coverage below which *P*-values were considered non-significant (*P*>0.001).
