# Supplementary material for: Mapping Small Effect Mutations in Saccharomyces cerevisiae: Impacts of Experimental Design and Mutational Properties
Source: G3 (Bethesda). 2014 Apr 29;4(7):1205–16. doi: 10.1534/g3.114.011783 (PMC4455770; doi:10.1534/g3.114.011783)
Supplement: Supporting Information [file supp_g3.114.011783_FileS1.pdf]

File S1

Extended Materials and Methods

Power analyses

Modeling exact allele frequencies in bulks: The goal of the deterministic step of the model was to calculate the frequency of mutant and reference alleles expected in each phenotypically divergent bulk of cells depending on total population size used for sorting (n), phenotypic selection cutoff used for isolating bulks (c), generations of growth after meiosis (g), mutation effect on mean expression (μ), mutation effect on standard deviation in expression (σ), and selection coefficient for the mutation (s). We modeled the total population distribution with respect to expression, X_T, as a mixture distribution of two populations, X_R and X_M, where X_R is the population carrying the reference allele at the causative locus and X_M is the population carrying the mutant allele at the causative locus, and tracked each population separately. Each population was assumed to follow a normal distribution with respect to expression:

(1) $X_R \sim N(0,1)$

(2) $X_M \sim N(\mu, \sigma^2)$

We represent the mean effect of a causal mutation, μ, relative to the standard deviation of the reference strain such that an increase of μ by 1 is equivalent to a shift in mean expression by one standard deviation (an approximately 7.5% change in expression in our data). Mutations were assumed not to influence sporulation efficiency or spore survival and X_R and X_M were started at equal frequencies. Populations were allowed to grow deterministically assuming a selection coefficient for the mutant causative allele of s. The reference allele was assumed to have fitness of 1 and after g generations the frequency of the mutant population in the population was:

(3) $f^0_{M_W} = \frac{(1-s)^g}{(1-s)^g+1}$

where W indicates the whole mutant or reference population prior to selection of phenotypic bulks (see figure S1 for diagram). The reference allele frequency was then the difference:

(4) $f^0_{R_W} = 1 - f^0_{M_W}$

After determining the frequencies of the mutant and reference populations, phenotypic selection using flow cytometry was modeled on the total population, X_T, at a predetermined population cutoff, c. The goal was to quantify the frequency of mutant and reference genotypes in each phenotypic bulk. Because X_T is a mixture distribution, the fractions of individuals with mutant and reference alleles present in each bulk were determined from

the reference and mutant phenotypic distributions X_R and X_M. For the high bulk, this required determining the quantiles q_{R_H} and q_{M_H} on X_R and X_M such that q_{R_H} and q_{M_H} equaled the same expression value $e_{T_H}$and c percent of the total population had higher expression than e_{T_H}.

(5) $$f_{R_W}^0 * q_{R_H} + f_{M_W}^0 * q_{M_H} = 1 - c$$

(6) $$e_{T_H} = \Phi^{-1}(q_{R_H}) = \mu + \sigma * \Phi^{-1}(q_{M_H})$$

Likewise, for the low bulk this required determining quantiles q_{R_L} and q_{M_L} on X_R and X_M such that q_{R_L} and q_{M_L} equaled the same expression value e_{T_L} and c percent of the total population had lower expression than e_{T_L}.

(7) $$f_{R_W}^0 * q_{R_L} + f_{M_W}^0 * q_{M_L} = c$$

(8) $$e_{T_L} = \Phi^{-1}(q_{R_L}) = \mu + \sigma * \Phi^{-1}(q_{M_L})$$

In both instances, $\Phi^{-1}(q)$ is the standard normal quantile function, H and L index the high and low bulks respectively, and e_{T_H} and $e_{T_L}$are the expression values for the high and low bulks relative to the entire population X_T. We solved the above equations numerically for q_{M_H} and q_{M_L} using *solnp* within Rsolnp (Ghalanos & Theussl 2006) by minimizing the following functions for the high and low bulks respectively:

(9) $$[f_{R_W}^0 * \Phi\left(\mu + \sigma * \Phi^{-1}(q_{M_H})\right) + f_{M_W}^0 * q_{M_H} + c - 1]^2$$

(10) $$[f_{R_W}^0 * \Phi\left(\mu + \sigma * \Phi^{-1}(q_{M_L})\right) + f_{M_W}^0 * q_{M_L} - c]^2$$

where Φ(y) is the cumulative distribution function for the standard normal distribution. From these quantiles, the frequencies of the mutant and reference alleles in the high and low bulks were calculated as the weighted proportion of mutant and reference alleles more extreme than the phenotypic cutoff:

(11) $$f_{M_H}^0 = \frac{f_{M_W}^0 * \Phi\left(\frac{e_{T_H} - \mu}{\sigma}\right)}{f_{R_W}^0 * \Phi(e_{T_H}) + f_{M_W}^0 * \Phi\left(\frac{e_{T_H} - \mu}{\sigma}\right)} = \frac{f_{M_W}^0 * q_{M_H}}{f_{R_W}^0 * q_{R_H} + f_{M_W}^0 * q_{M_H}} = \frac{f_{M_W}^0 * q_{M_H}}{1-c}$$

(12) $$f_{R_H}^0 = 1 - f_{M_H}^0$$

(13) $$f_{M_L}^0 = \frac{f_{M_W}^0 * \Phi\left(\frac{e_{T_L} - \mu}{\sigma}\right)}{f_{R_W}^0 * \Phi(e_{T_L}) + f_{M_W}^0 * \Phi\left(\frac{e_{T_L} - \mu}{\sigma}\right)} = \frac{f_{M_W}^0 * q_{M_L}}{f_{R_W}^0 * q_{R_L} + f_{M_W}^0 * q_{M_L}} = \frac{f_{M_W}^0 * q_{M_L}}{c}$$

(14) $$f_{R_L}^0 = 1 - f_{M_L}^0$$

To model the additional growth necessary to create libraries from the sorted bulks, each bulk was allowed to undergo another g generations of growth, assuming that the relative fitness (1-*s*) between genotypes with the mutant and reference alleles of the site affecting fluorescence was the same before and after bulk selection:

(15) $f_{M_H}^1 = \frac{f_{M_H}^0 (1-s)^g}{f_{M_H}^0 (1-s)^g + f_{R_H}^0 * 1}$

(16) $f_{M_L}^1 = \frac{f_{M_l}^0 (1-s)^g}{f_{M_L}^0 (1-s)^g + f_{R_L}^0 * 1}$

Simulation of allele frequency estimates from sequencing data: Using the deterministic allele frequencies described above, we simulated the library creation and sequencing processes by drawing the proportion of 'reads' containing the mutant allele from a binomial distribution in each bulk independently:

(17) $T_{M_H} \sim B(V_H, F_{M_H})$

(18) $T_{M_L} \sim B(V_L,\ F_{M_L})$

where V is the distribution of sequencing coverage and F the mutant allele frequency distribution. The sequencing coverage distribution was simulated as a negative binomial distribution (Robinson and Smyth 2007, 2008):

(19) $V \sim NB(\alpha, \beta)$ with mean $\frac{\alpha}{\beta}$ and variance $\frac{\alpha(\beta+1)}{\beta^2}$

To adjust coverage, we varied β (inverse scale) because our data suggested α (shape) was approximately 80 regardless of sequencing depth. Average coverage was set to reflect coverage after mapping and we did not explicitly model sequencing error. To account for sampling during library creation, the mutant allele frequencies were simulated from the deterministic frequencies assuming a binomial distribution:

(21) $F_{M_H} \sim \frac{B\left(n, f_{M_H}^1\right)}{n}$

(22) $F_{M_L} \sim \frac{B\left(n, f_{M_L}^1\right)}{n}$

Reference 'reads' were then assumed to make up the difference between the coverage and the number of mutant 'reads'

(23) $t_{R_H} \sim v_H - t_{M_H}$

(24) $t_{R_L} \sim v_L - t_{M_L}$

A G-test was performed on the counts t_{M_H}, t_{M_L}, t_{R_L} and t_{R_H} to determine significance. Power was calculated as the frequency of simulations where the *P*-value was below 0.001, representing a Bonferonni correction assuming 50 possible mutations.

Comparison between G-test and Fisher's exact test*:* The Fisher's exact test commonly used in the analysis of next generation sequencing data (Kofler *et al.* 2011) assumes that the row and column totals of the two-by-two

contingency table are fixed. This assumption is violated by sequencing data, however, because coverage for each allele results from sampling reads from an underlying distribution. When marginal totals are free to vary, the G-test is more appropriate than the Fisher's exact test. We analyzed our data using both tests and found that their results were very similar (although not identical) except when sequencing coverage was low (Figure S11).

DNA library preparation

Genomic DNA libraries were produced in parallel by modifying a low cost method developed for Illumina sequencing (Rohland and Reich 2012). Briefly, DNA was sheared, Illumina adapters were attached by blunt-end ligation and indexed using PCR. Between enzymatic reactions, DNA was cleaned using custom MagNA beads (Carboxyl-modified Sera-Mag Magnetic Speed-beads in a PEG/NaCl buffer) as a lower-cost substitute for AMPure XP kit. For each sample, 2 µg of genomic DNA (120 µl) was sheared to an average fragment size of 400 bp with a Covaris S220 instrument (Duty cycle: 10%, intensity: 4, cycles/burst: 200, time: 55 s). 1 µg (60 µl) of sheared DNA was purified in 96 µl (1.6x) of MagNA bead solution and resuspended in 20 µl of water. Blunt-end repair was performed using a NEB Quick Blunting Kit by mixing 19 µl of DNA with 2.5 µl of blunting buffer, 2.5 µl of 1 mM dNTP mix and 1 µl of blunt enzyme mix. This mix was incubated for 20 min at 12°C followed by 15 min at 37°C. DNA was then cleaned up in 2x MagNA beads and eluted in 25 µl of water. Next, adapters were ligated using a NEB Quick Ligation Kit. 23.8 µl of blunt DNA was mixed with 30 µl of ligation buffer, 4 µl of P5 + P7 adapter mix (100 µM each) and 1.2 µl of Quick T4 DNA ligase and incubated at 25°C for 20 min. DNA was then cleaned in 1.6x beads, eluted in 40 µl and nick-fill in was done using Bst DNA Polymerase Large Fragment from NEB. 39 µl of DNA sample was mixed with 5 µl of ThermoPol buffer, 4 µl of 25 mM dNTP mix and 2 µl of Bst DNA polymerase (2 U/µl). After 20 min at 37°C, samples were mixed with 1.6x MagNA beads and eluted in 30 µl water. KAPA HiFi PCR Kit was used for indexing PCR: 10 µl of template DNA was mixed with 5 µl of HiFi buffer, 0.75 µl of 10 mM dNTP mix, 0.75 µl primer IS4 (10 µM), 0.75 µl indexing primer (10 µM), 7.25 µl sterile H_2O and 0.5 µl KAPA HiFi polymerase (1 U/µl). PCRs were incubated at 95°C for 4 min followed by 12 cycles at 98°C for 20 s, 64°C for 15 s and 72°C for 20 s with a final extension at 72°C for 5 min. PCR products were then cleaned up in 1.6x MagNA beads and eluted in 40 µl of water. Samples were then processed at the UM Sequencing Core Facility. For each sample, DNA concentration was quantified through qPCR with primers targeting P5 and P7 adapters and using an Agilent 2100 Bioanalyzer. Equimolar amounts of each sample were pooled together for multiplexed sequencing before gel electrophoresis size selection of DNA fragments ranging from 350 bp to 850 bp on a 1% agarose gel. The 8 libraries produced for this project (high- and low-fluorescing bulks for each of the three mutants

plus the original non-mutagenized strain and the mapping strain, all of which were haploid) were combined with 16 libraries constructed for other projects and subjected to 100 bp paired-end sequencing in one lane on Illumina HiSeq2000 platform. Oligonucleotide sequences used for library preparation are listed in Table S1 and barcode sequences used for multiplexing in Table S2. Because average sequencing depth was lower than 75x for two of the samples (YPW89 low bulk and YPW102 low bulk), we decided to re-sequence the corresponding genomic libraries in an independent sequencing lane using the same procedure. All data from the two runs of sequencing were combined for analyses presented in this study.

Tetrad dissection-based approach for mapping

In addition to the high-sensitivity method described above, we mapped the causative mutation altering YFP expression in several mutants including YPW89, YPW94 and YPW102 using a tetrad dissection-based approach (Birkeland *et al.* 2010). First, mutants YPW89 and YPW94 were crossed to Y39 (*MATα leu2Δ0 ura3Δ0* P_{TDH3}*-YFP*) and YPW102 was crossed to Y85 (*MATα met17Δ0 ura3Δ0* P_{TDH3}*-YFP*). Resulting diploids were sporulated in KAc medium, several tetrads were dissected and individual spores were grown on YPD (11 tetrads for YPW89xY39, 8 tetrads for YPW94xY39 and 9 tetrads for YPW102xY85). The fluorescence level of the resulting colonies was quantified through flow cytometry. Each spore was grown in YPD to saturation, then diluted in SC-Arg medium and grown to log-phase at 30°C. Fluorescence (FL1-A) and forward scatter (FSC-A) of thousands of cells were recorded using a HyperCyt Autosampler (IntelliCyt Corp.) coupled to a BD Accuri C6 Flow Cytometer (533/30 nm optical filter used for YFP acquisition). Based on these data, a mutant phenotype was assigned for 2 of the 4 spore progeny from each tetrad. For tetrads derived from YPW89 and YPW94 (increased YFP expression), the two progeny with highest median of FL1-A/FSC-A were considered as mutants. For tetrads derived from YPW102 (decreased YFP expression), the two progeny with lowest median of FL1-A/FSC-A were considered as mutants. These mutant progeny were then cultured separately to saturation in YPD and mixed evenly to a final volume of 2.5 ml. 22 progeny were mixed together for YPW89, 16 for YPW94 and 18 for YPW102. For each pool, genomic DNA was extracted using a Gentra Puregene Yeast/Bacteria Kit from QIAGEN. Next, 2 µg of DNA was sheared with a Covaris S220 instrument and genomic libraries were prepared using NEBNext E6040 kit. An in-line barcoding strategy was adopted for multiplexing. Briefly, 3' A overhang was added to end-repaired DNA fragments. Then, barcoded adapters were ligated to dA-tailed DNA, creating Y-shaped products whose extremities are single-stranded. PCR using standard Illumina primers allowed the addition of adapter sequences attaching to Illumina flow cells. PCR products ranging from 400bp to 800bp were size

selected on an agarose gel. Barcodes, adapters and PCR primer sequences are listed in Table S3 and Table S4. 22 libraries were pooled together and 100 bp paired-end reads were sequenced on a single lane of HiSeq2000 flow cell at the University of Michigan Sequencing Core. Sequencing data were analyzed through the same pipeline as described above, except that only mutant segregant pools were sequenced in this case. G-tests were performed by comparing observed mutation frequency in the mutant pool to a null expectation of 0.5.

Quantification of allele frequencies through pyrosequencing

To assess the accuracy of allele frequency estimates obtained through Illumina sequencing, quantitative genotyping of the low and high fluorescence bulks was performed for three variable sites in each mutant using pyrosequencing. These included the site with strongest allele frequency difference between bulks as well as two sites showing no significant difference in allele frequency. Pyrosequencing assays (see File S3) were designed following manufacturer instructions (PyroMark Assay Design software from QIAGEN), except that a universal biotinylated primer was used to reduce the cost. For each variant assessed, PCR reactions were performed as previously described (Aydin *et al.* 2006) on 5 different genomic DNA templates from the original haploid mutant, the haploid mapping strain, the F1 diploid hybrid and the low and high fluorescence haploid segregants. Quantitative genotyping was performed on a PyroMark ID instrument following the protocol described in Wittkopp (2011). Data from parental strains and the hybrid were used to correct for potential PCR or sequencing biases. Knowing that true allele frequencies are 1, 0 and 0.5 in the mutant, mapping strain, and hybrid, a 2nd degree polynomial regression model was fitted to the observed data and used to correct allele frequencies in the segregant bulks.

Literature cited

Aydin, A., M. R. Toliat, S. Bähring, C. Becker, and P. Nürnberg, 2006 New universal primers facilitate Pyrosequencing. Electrophoresis 27: 394–397.

Birkeland, S. R., N. Jin, A. C. Ozdemir, R. H. Lyons, L. S. Weisman *et al.*, 2010 Discovery of mutations in *Saccharomyces cerevisiae* by pooled linkage analysis and whole-genome sequencing. Genetics 186: 1127–1137.

Ghalanos, A., & Theussl, S. (2012). Rsolnp: General Non-linear Optimization Using Augmented Lagrange Multiplier Method. R package version 1.14.

Kofler, R., R. V. Pandey, and C. Schlötterer, 2011 PoPoolation2: identifying differentiation between populations using sequencing of pooled DNA samples (Pool-Seq). Bioinformatics (Oxford, England) 27: 3435–3436.

Robinson, M. D., and G. K. Smyth, 2007 Moderated statistical tests for assessing differences in tag abundance. Bioinformatics (Oxford, England) 23: 2881–2887.

Robinson, M. D., and G. K. Smyth, 2008 Small-sample estimation of negative binomial dispersion, with applications to SAGE data. Biostatistics (Oxford, England) 9: 321–332.

Rohland, N., and D. Reich, 2012 Cost-effective, high-throughput DNA sequencing libraries for multiplexed target capture. Genome research 22: 939–946.

Wittkopp, P. J., 2011 Using pyrosequencing to measure allele-specific mRNA abundance and infer the effects of *cis*- and *trans*-regulatory differences. Molecular Methods for Evolutionary Genetics 772: 297–317.

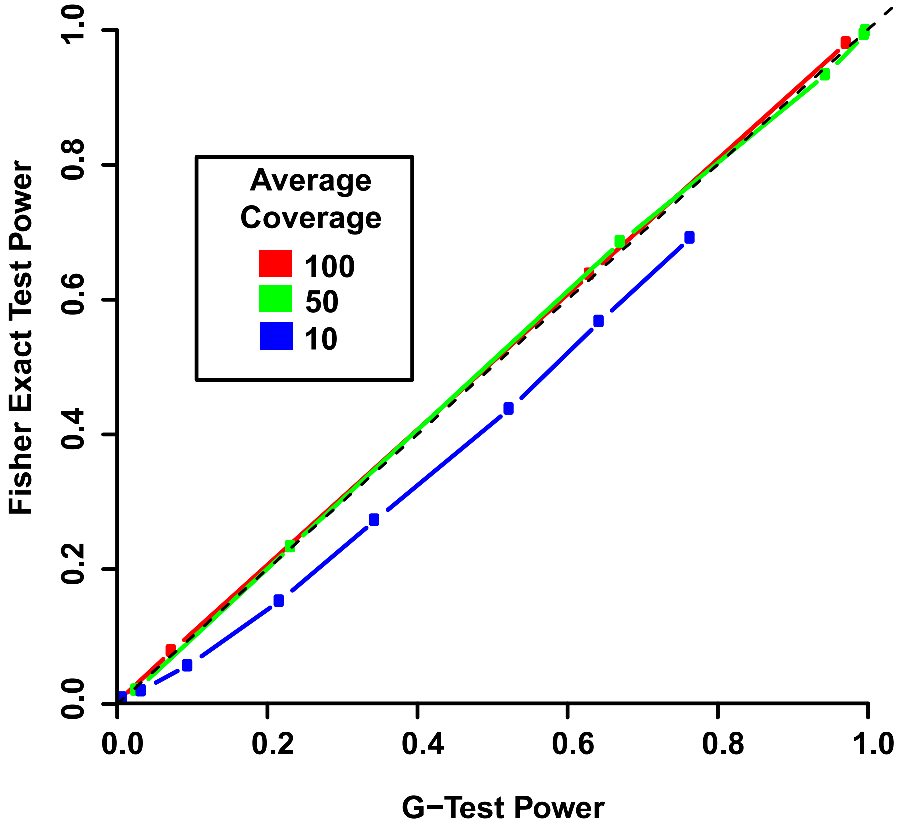

Figure S11 Comparison of statistical power using Fisher's exact test and G-test. Power to detect a significant difference in allele frequency between bulks for different mutation effect sizes and sequencing depths is shown. Dots on each line represent different mutation effects ranging from 0% to +25% (bottom left to top right) relative to WT mean expression. Fixed parameter values were: Standard Deviation = 100%, Selection Coefficient = 0.03, Population Size = 10^7, Cutoff Percent = 5%, Generations = 20.

Table S1 Sequences of oligonucleotide adapters used for library preparation in the FACS-based mapping approach.

Oligo ID	Oligo Sequence 5'-3' (* indicates Phosphorothioate bound)
IS1_adapter.P5	A*C*A*CTCTTTCCCTACACGACGCTCTTCCGA*T*C*T
IS2_adapter.P7	G*T*G*ACTGGAGTTCAGACGTGTGCTCTTCCGA*T*C*T
IS3_adapter.P5+P7	A*G*A*TCGGAAG*A*G*C
IS4_indPCR.P5	AATGATACGGCGACCACCGAGATCTACACTCTTTCCCTACACGACGCTCTT

Table S2 Indexing oligos and barcodes used for library preparation in the FACS-based mapping approach.

Oligo ID	Oligo Sequence 5'-3' (Lowercase: Index barcode)	Barcode	Sample
indexing4	CAAGCAGAAGACGGCATACGAGATttgatccGTGACTGGAGTTCAGACGTGT	GGATCAA	YPW89.low
indexing5	CAAGCAGAAGACGGCATACGAGATatcttgcGTGACTGGAGTTCAGACGTGT	GCAAGAT	YPW94.low
indexing6	CAAGCAGAAGACGGCATACGAGATtctccatGTGACTGGAGTTCAGACGTGT	ATGGAGA	YPW102.low
indexing12	CAAGCAGAAGACGGCATACGAGATacttcaaGTGACTGGAGTTCAGACGTGT	TTGAAGT	YPW89.high
indexing13	CAAGCAGAAGACGGCATACGAGATtgatagtGTGACTGGAGTTCAGACGTGT	ACTATCA	YPW94.high
indexing14	CAAGCAGAAGACGGCATACGAGATgatccaaGTGACTGGAGTTCAGACGTGT	TTGGATC	YPW102.high
indexing19	CAAGCAGAAGACGGCATACGAGATgagattcGTGACTGGAGTTCAGACGTGT	GAATCTC	WT
indexing20	CAAGCAGAAGACGGCATACGAGATgagcatgGTGACTGGAGTTCAGACGTGT	CATGCTC	Mapping.Strain

Only eight samples used in this study are shown. These eight samples were multiplexed with 16 other samples using the following barcodes: 1-TCGCAGG, 2-CTCTGCA, 3-CCTAGGT, 4-GGATCAA, 5-GCAAGAT, 6-ATGGAGA, 7-CTCGATG, 8-GCTCGAA, 9-ACCAACT, 10-CCGGTAC, 11-AACTCCG, 12-TTGAAGT, 13-ACTATCA, 14-TTGGATC, 15-CGACCTG, 16-TAATGCG, 17-AGGTACC, 18-TGCGTCC, 19-GAATCTC, 20-CATGCTC, 21-ACGCAAC, 22-GCATTGG, 23-GATCTCG, 24-CAATATG.

Table S3 Sequences of oligonucleotide adapters used for library preparation in the tetrad-based mapping approach.

Oligo ID	Oligo Sequence 5'-3'
Indexed adapter 1	ACACTCTTTCCCTACACGACGCTCTTCCGATCTNNNNNNT
Indexed adapter 2	NNNNNNAGATCGGAAGAGCGGTTCAGCAGGAATGCCGAG
PCR primer 1	AATGATACGGCGACCACCGAGATCTACACTCTTTCCCTACACGACGCTCTTCCGATCT
PCR primer 2	CAAGCAGAAGACGGCATACGAGCTCTTCCGATCT

Underlined: barcode. Color: Same color shows complementary regions where annealing occurs during PCR.

Table S4 Barcodes used for library preparation in the tetrad dissection-based mapping approach.

Barcode	Sample
ACCAGG	Y1
AAGGCC	Y39
TATTCG	Y54 x Y85
CGGAAC	Y85
ATACCT	Y89 x Y39
ACACGA	Y94 x Y39
CACATA	Y102 x Y85

Only seven samples used in this study are presented. These seven samples were multiplexed with 14 other samples using the following barcodes: 1-ACCAGG, 2-AAGGCC, 3-TCTGAT, 4-CAAGTG, 5-TACGTT, 6-TATTCG, 7-CGGAAC, 8-ATACCT, 9-GTGCTG, 10-GGCGTA, 11-TGCACG, 12-CTACGC, 13-ACACGA, 14-CCGTAG, 15-GTAACA, 16-GTGTAT, 17-AGGTTC, 18-CACATA, 19-AGTTGG, 20-GCTCAA, 21-TTGACT, 22-TCTCGG.
