# Supplementary material for: Mapping Small Effect Mutations in Saccharomyces cerevisiae: Impacts of Experimental Design and Mutational Properties
Source: G3 (Bethesda). 2014 Apr 29;4(7):1205–16. doi: 10.1534/g3.114.011783 (PMC4455770; doi:10.1534/g3.114.011783)
Supplement: Supporting Information [file supp_g3.114.011783_FileS4.pdf]

File S4

Robustness of the BSA-seq approach

Impact of genomic position on mapping success: To determine the limits of our bulk segregant mapping protocol, we tested whether the three causal mutations we identified would have been successfully mapped if they had been located somewhere else in the genome. This might not be the case if the power to map a mutation of a given effect size was uneven across the genome, either because of random fluctuation in sequencing depth or because of reads failing to align uniquely to the genome. To examine this possibility, we first computed for each bulk sample the sequencing depth at every genomic position using *genomecov* tool in BEDTools v2.17.0 (Quinlan and Hall 2010). We then inferred, for each genomic position in each segregant bulk, the number of mutant and wild type alleles we would have observed if the site was causative given the coverage of the position and the mutation frequency at the actual causative site. We then calculated the fraction of genomic positions for which a mutation with the same effect as the actual causative mutation would have been detected and called significant using the analysis pipeline described in Figure 4.

Depending on the mutant considered, we found that 2.9% to 3.4% of genomic positions were not covered by any sequencing reads in at least one sample (Figure S10A, left bars), making it impossible to test for a significant association. Additionally, 4.1% to 5.1% of genomic positions failed to meet the minimum cutoff of 10 reads in the merged bulks that we required for the site to be called as a high confidence SNP (Figure S10A, middle bars). These sites were thus not tested for a significant association with the fluorescence phenotype and the causative mutation would have remained undetected if located at one of these positions. Finally, we found that 4.2% to 4.9% of sites had insufficient sequencing coverage to yield a significant phenotypic association in a G-test (Figure S10A, right bars), most of which also failed to meet the 10 read minimum criterion to be called a SNP. Low sequence read coverage at these sites could be caused by random fluctuations in sequencing depth or problems aligning sequence reads that contain these sites.

To determine how often sites with low coverage resulted from poor alignment of sequence reads, we assessed mappability for each position in the reference genome using software from the GEM library (Derrien *et al.* 2012). A genomic site was considered to have perfect mappability if and only if every possible read overlapping that site aligned uniquely to the correct genomic position (Stevenson *et al.* 2013). Aligning 100 bp sequences to the reference genome while allowing up to five mismatches showed imperfect mappability for 6.8% of the *S. cerevisiae* genome (Figure S10B). More than 97% of these sites were included in at least one of the three groups of problematic

sites described above (Figure S10B), indicating that the inability to uniquely map sequence reads, rather than random variation in sequencing depth, was responsible for the vast majority of sites with low coverage in our dataset. This interpretation is further supported by the genome-wide distributions of sequencing coverage showing two peaks -- one centered at the mean coverage for each sample and the other at 0 (see Figure S9).

If a causative mutation occurs in a low mappability region, it would remain undetected, but linked mutations could still yield a significant association of the phenotype to a broader genomic region. However, such mapping by linkage is likely to occur only if the average distance between mutations is smaller than the extent of genetic linkage. Linkage extends approximately 50 kb after a single generation of meiosis in *S. cerevisae* (Mortimer *et al.* 1991). Given the number of mutations in each mutant isolated in Gruber *et al.* (2012), an average of one mutation is expected every 255 kb, making linkage unlikely for most pairs of sites. Assuming all of these mutations are indeed unlinked, we conclude that a small portion of the genome (~4% on Figure S10A, middle bars) is unsuitable to mapping in these mutants using short-read data regardless of sequencing depth.

Impact of decreased sequencing depth on mapping success: To determine how variant calling might have affected our results, we assessed the total number of mutations called for each mutant using the bulk sequencing data when reads from the SAM files were randomly subsampled to a genome coverage ranging from 10x to 110x using the Picard (v1.97) command-line tool *DownsampleSam* (http://picard.sourceforge.net). For all three mutants, a steep drop was observed in the total number of mutations called at 10x coverage relative to 25x coverage (Figure S10C). As expected, sites with the lowest read counts for mutant alleles were the first to be missed when sequencing depth was decreased. Interestingly, the only mutation missed in YPW89 mutant when sequencing coverage was reduced to 75x was the causative mutation. This was because this mutation also strongly reduced fitness (Table 3), causing the number of mutant alleles in both bulks to be very low. With decreased coverage, the number of sequencing reads overlapping this site quickly fell below the minimum required for detection as a high confidence SNP.

Finally, we determined how the significance of G-tests used to identify associated sites varied with sequencing depth. The read number for reference and mutant alleles at the causative site were divided by the same values, so that the average sequencing depth between low and high bulks at the site was 80, 70, 60, 50, 40, 30, 20 and 10. We found that the statistical significance of associations between causal sites and YFP fluorescence decreased linearly with sequencing depth, but at different rates for different mutants (Figure S10D). For YPW102, as few as 10 reads overlapping the causal site were required to detect a significant association, whereas 15 and 41 reads were

required in YPW94 and YPW89, respectively. YPW89 was again found to be the most sensitive to a decrease in sequencing depth despite having the strongest effects on mean fluorescence because its effects on fitness decreased its frequency in both bulks (Figure S10C-D).

Literature cited

Derrien, T., J. Estellé, S. Marco Sola, D. G. Knowles, E. Raineri *et al.*, 2012 Fast Computation and Applications of Genome Mappability. PLoS ONE 7: e30377.

Gruber, J. D., K. Vogel, G. Kalay, and P. J. Wittkopp, 2012 Contrasting Properties of Gene-Specific Regulatory, Coding, and Copy Number Mutations in *Saccharomyces cerevisiae*: Frequency, Effects, and Dominance. PLoS genetics 8: e1002497.

Mortimer, R. K., D. Schild, C. R. Contopoulou, and J. A. Kans, 1991 [57] Genetic and physical maps of *Saccharomyces cerevisiae*. Methods in Enzymology 194: 827–863.

Quinlan, A. R., and I. M. Hall, 2010 BEDTools: a flexible suite of utilities for comparing genomic features. Bioinformatics (Oxford, England) 26: 841–842.

Stevenson, K. R., J. D. Coolon, and P. J. Wittkopp, 2013 Sources of bias in measures of allele-specific expression derived from RNA-seq data aligned to a single reference genome. BMC genomics 14: 536.
